# Supplementary material for: Design, Synthesis and Biological Evaluation of Novel Pleuromutilin Derivatives Containing 6-Chloro-1-R-1H-pyrazolo[3,4-d]pyrimidine-4-amino Side Chain
Source: Molecules. 2023 May 8;28(9):3975. doi: 10.3390/molecules28093975 (PMC10180054; doi:10.3390/molecules28093975)
Supplement: Supplementary file 1 [file molecules-28-03975-s001.zip › molecules-2328605-supplementary.pdf]

Design, synthesis and biological evaluation of novel pleuromutilin derivatives containing 6-chloro-1-R-1*H*-pyrazolo[3,4-*d*]pyrimidine-4-amino side chain

Jun Wang[1], Yu-Han Hu[1], Ke-Xin Zhou[1], Wei Wang[1], Fei Li[1], Ke Li[1], Guang-Yu Zhang[1], You-Zhi Tang[1,2*]

[1]*Guangdong Provincial Key Laboratory of Veterinary Pharmaceutics Development and Safety Evaluation, College of Veterinary Medicine, South China Agricultural University, Guangzhou 510642, China*

[2]*Guangdong Laboratory for Lingnan Modern Agriculture, Guangzhou, 510642, China*

Correspondence should be addressed:

College of Veterinary Medicine, South China Agricultural University, No. 483 Wushan Road, Tianhe District, Guangzhou 510642, China

You-Zhi Tang,

Table S1 Structures of corresponding intermediates compounds **12a ~ 28a**, and the MIC of compounds **12a ~ 28a** against MRSA.

Compound No	Structure	MIC against MRSA
	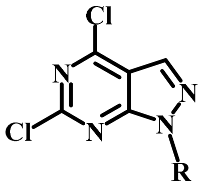	
12a	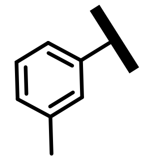	> 64
13a	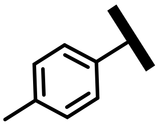	> 64
14a	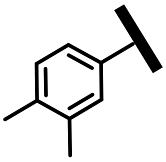	> 64
15a	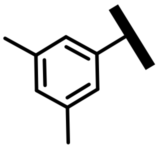	> 64
16a	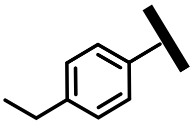	> 64
17a	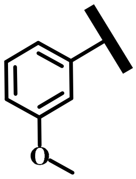	> 64
18a	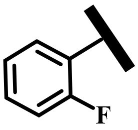	> 64
19a	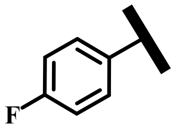	> 64

Compound No	Structure	MIC against MRSA
20a	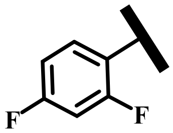	> 64
21a	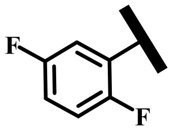	> 64
22a	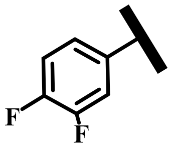	> 64
23a	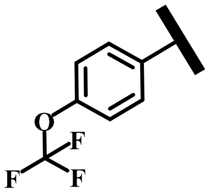	> 64
24a	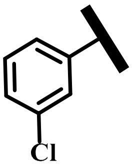	> 64
25a	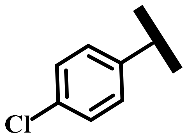	> 64
26a	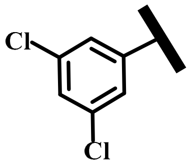	> 64
27a	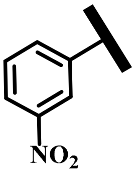	> 64
28a	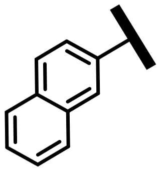	> 64

Table S2 MIC and MBC (μg/mL) values of compounds **12b ~ 28b** against *S. Typhimurium* ATCC 14028, *K. Pneumonia* ATCC 70063, *E. Coli* ATCC 25922 and *E. faecalis* ATCC 29212

Compound No	R	MIC/MBC(μg/mL)			
		S.Typhimurium ATCC 14028	*K. Pneumonia* ATCC 70063	*E. Coli* ATCC 25922	*E. Faecalis* ATCC 29212
		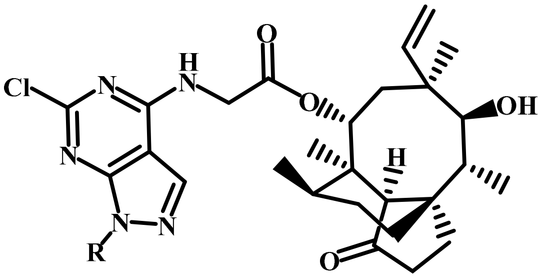			
12b	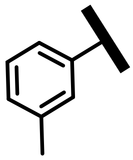	> 64	> 64	> 64	> 64
13b	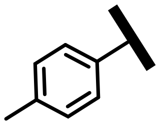	> 64	> 64	> 64	> 64
14b	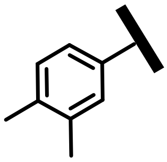	> 64	> 64	> 64	> 64
15b	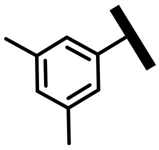	> 64	> 64	> 64	> 64
16b	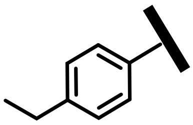	> 64	> 64	> 64	> 64

Compound No	R	MIC/MBC(μg/mL)			
		S.Typhimurium ATCC 14028	*K. Pneumonia* ATCC 70063	*E. Coli* ATCC 25922	*E. Faecalis* ATCC 29212
17b	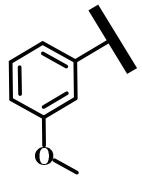	> 64	> 64	> 64	> 64
18b	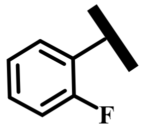	> 64	> 64	> 64	> 64
19b	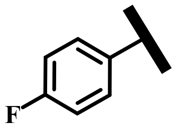	> 64	> 64	> 64	> 64
20b	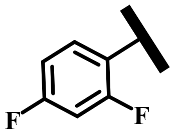	> 64	> 64	> 64	> 64
21b	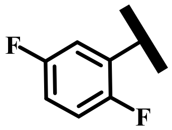	> 64	> 64	> 64	> 64
22b	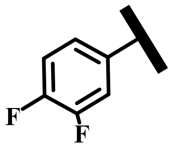	> 64	> 64	> 64	> 64
23b	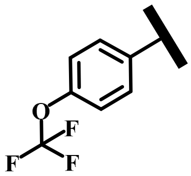	> 64	> 64	> 64	> 64
24b	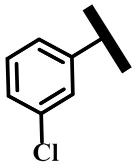	> 64	> 64	> 64	> 64

Compound No	R	MIC/MBC(μg/mL)			
		S.Typhimurium ATCC 14028	*K. Pneumonia* ATCC 70063	*E. Coli* ATCC 25922	*E. Faecalis* ATCC 29212
25b	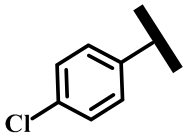	> 64	> 64	> 64	> 64
26b	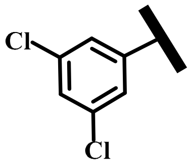	> 64	> 64	> 64	> 64
27b	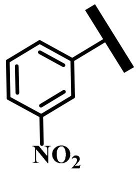	> 64	> 64	> 64	> 64
28b	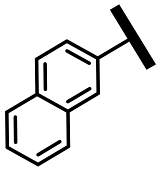	> 64	> 64	> 64	> 64
Tiamulin		> 64	> 64	> 64	> 64

Table S3 MIC and MBC (μg/mL) values of compounds **12c ~ 28c** against *S. Typhimurium* ATCC 14028, *K. Pneumonia* ATCC 70063, *E. Coli* ATCC 25922 and *E. faecalis* ATCC 29212

Compound No	R	MIC/MBC(μg/mL)			
		S. Typhimurium ATCC 14028	*K. Pneumonia* ATCC 70063	*E. Coli* ATCC 25922	*E. faecalis* ATCC 29212
	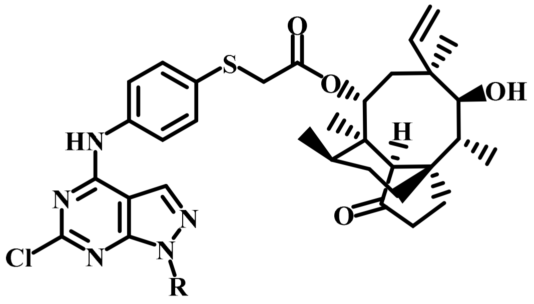				
12c	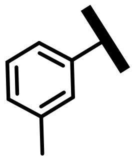	> 64	> 64	> 64	> 64
13c	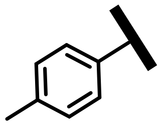	> 64	> 64	> 64	> 64
14c	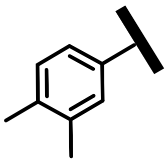	> 64	> 64	> 64	> 64
15c	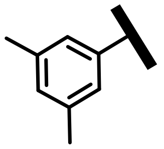	> 64	> 64	> 64	> 64
16c	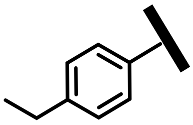	> 64	> 64	> 64	> 64

Compound No	R	MIC/MBC(μg/mL)			
		S. Typhimurium ATCC 14028	*K. Pneumonia* ATCC 70063	*E. Coli* ATCC 25922	*E. faecalis* ATCC 29212
17c	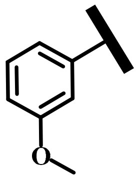	> 64	> 64	> 64	> 64
18c	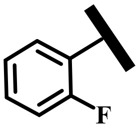	> 64	> 64	> 64	> 64
19c	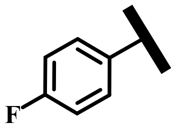	> 64	> 64	> 64	> 64
20c	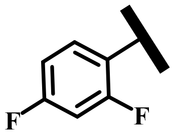	> 64	> 64	> 64	> 64
21c	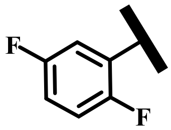	> 64	> 64	> 64	> 64
22c	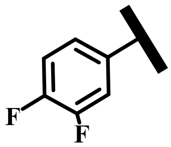	> 64	> 64	> 64	> 64
23c	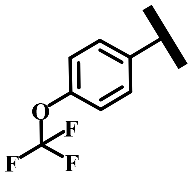	> 64	> 64	> 64	> 64
24c	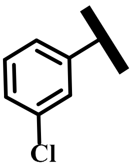	> 64	> 64	> 64	> 64

Compound No	R	MIC/MBC(μg/mL)			
		S. Typhimurium ATCC 14028	*K. Pneumonia* ATCC 70063	*E. Coli* ATCC 25922	*E. faecalis* ATCC 29212
25c	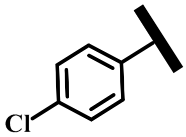	> 64	> 64	> 64	> 64
26c	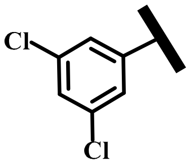	> 64	> 64	> 64	> 64
27c	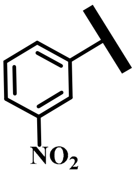	> 64	> 64	> 64	> 64
28c	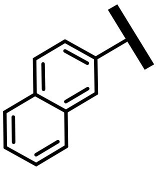	> 64	> 64	> 64	> 64
Tiamulin		> 64	> 64	> 64	> 64

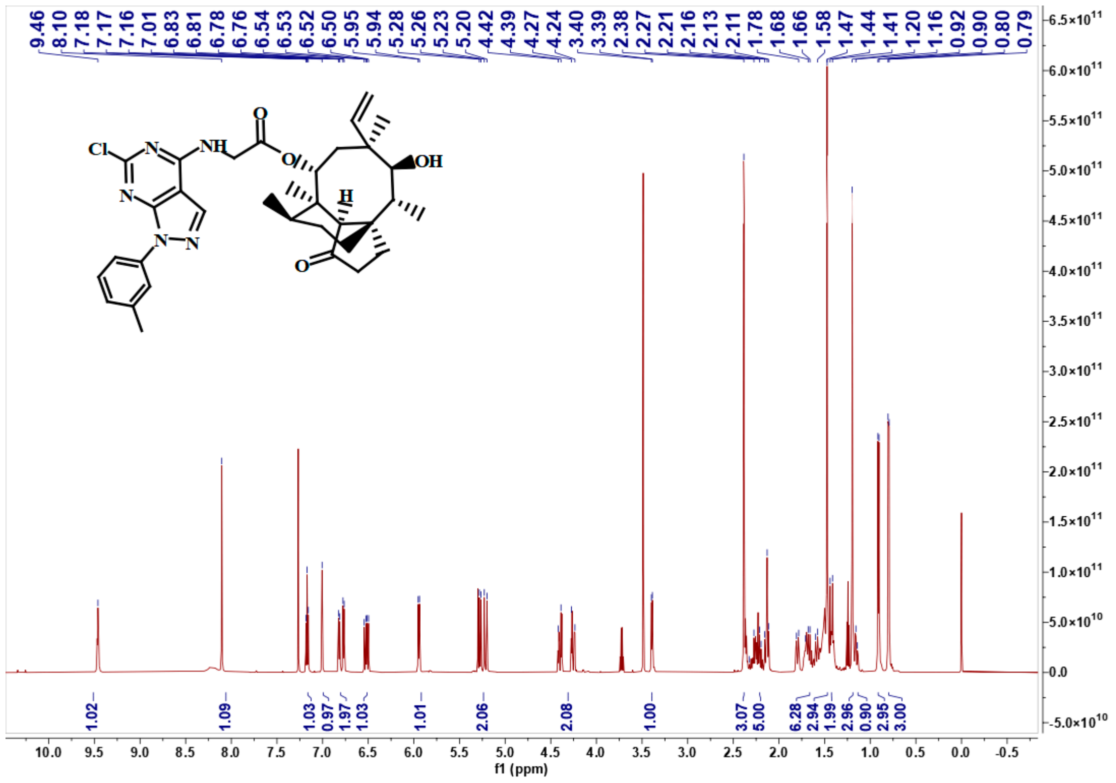

Figure S1.^{1}H-NMR spectrum (CDCl3,600MHz) of compound **12b**.

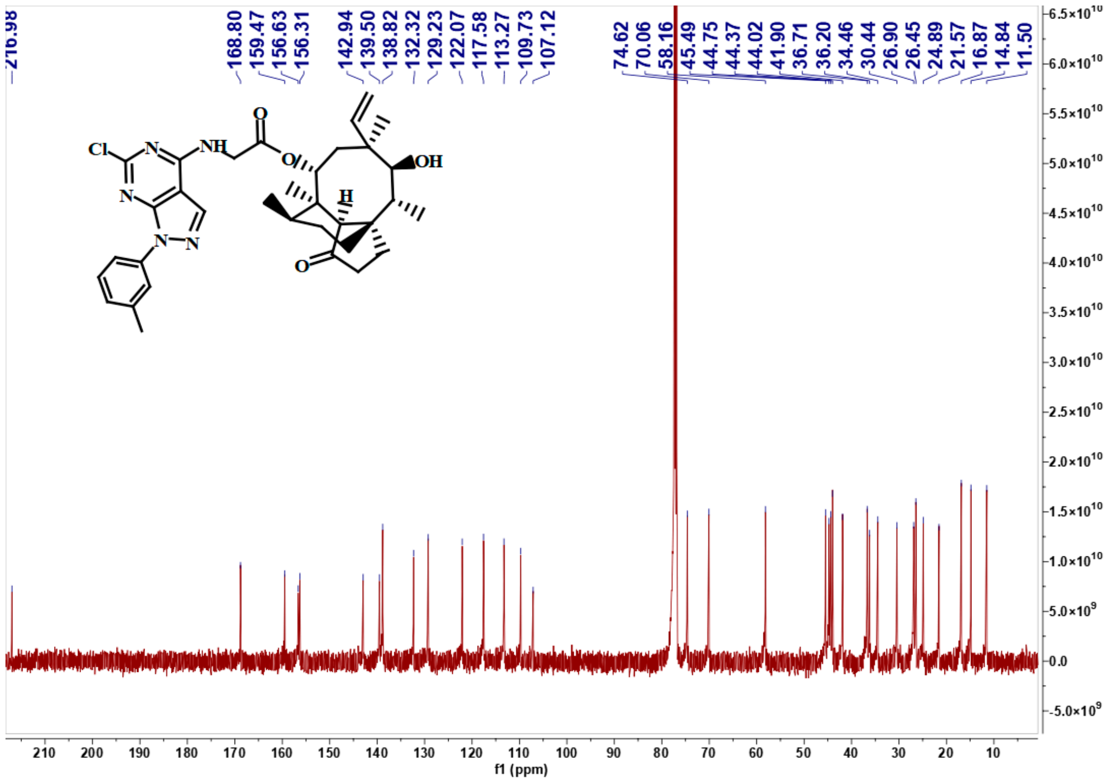

Figure S2.^{13}C-NMR spectrum (CDCl3, 151MHz) of compound **12b**.

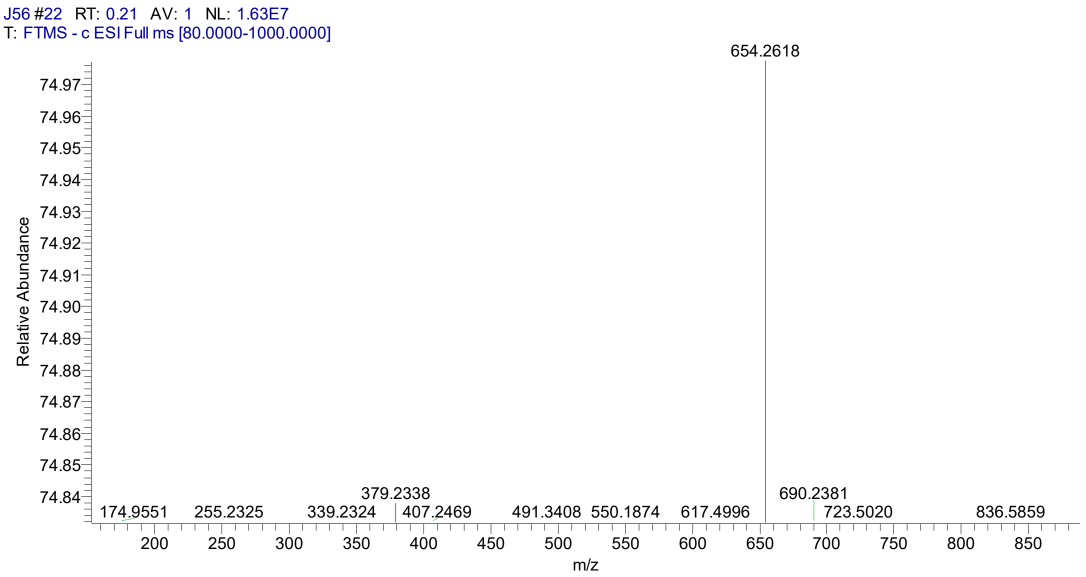

Figure S3.HR Mass spectrum (ESI) of compound **12b**.

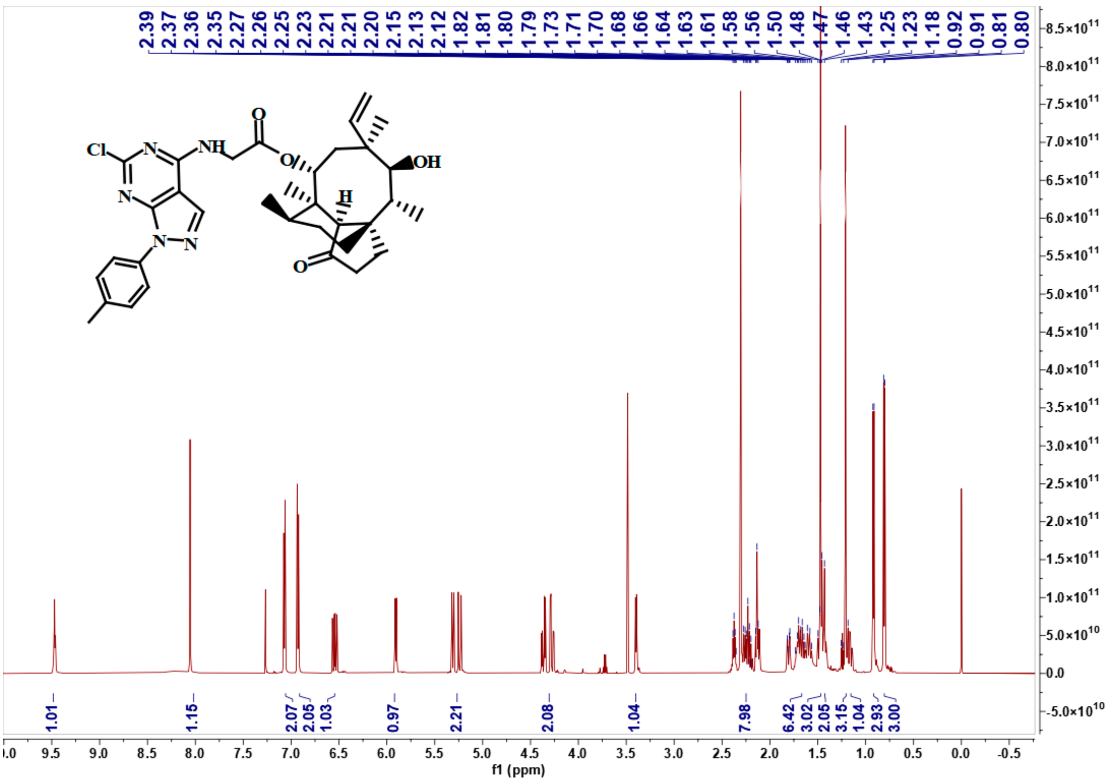

Figure S4.^{1}H-NMR spectrum ($CDCl_3$, 600MHz) of compound **13b**.

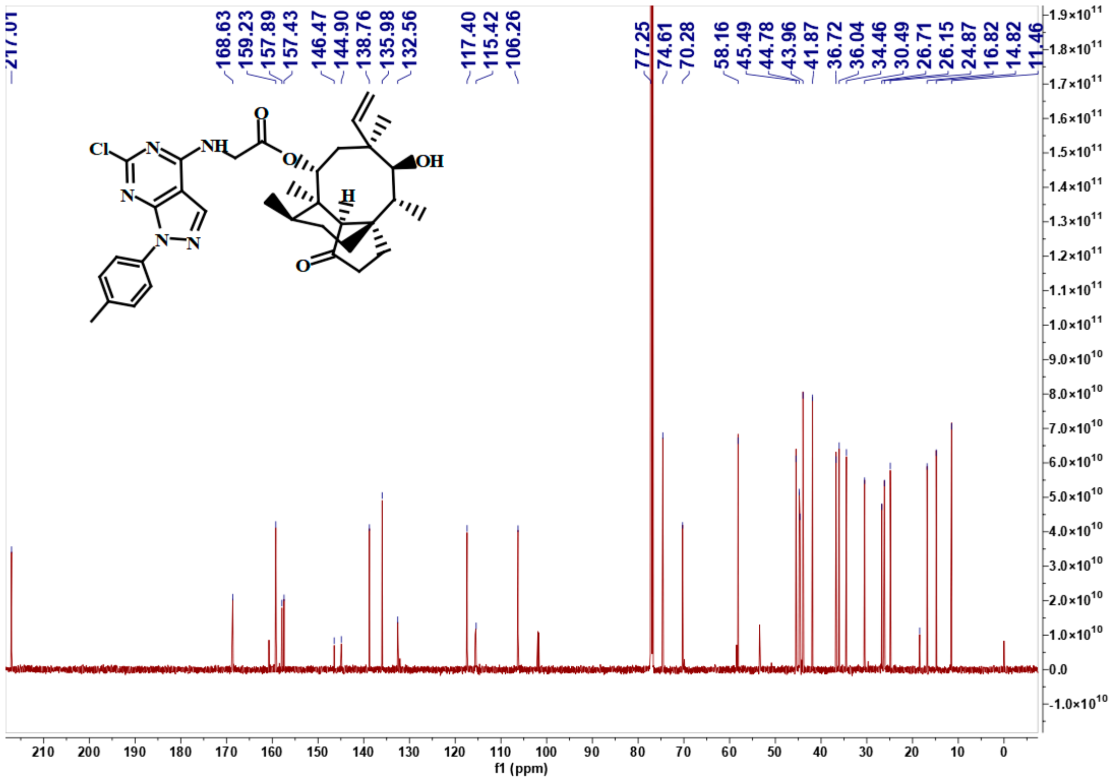

Figure S5.^{13}C-NMR spectrum ($CDCl_3$, 151MHz) of compound **13b**.

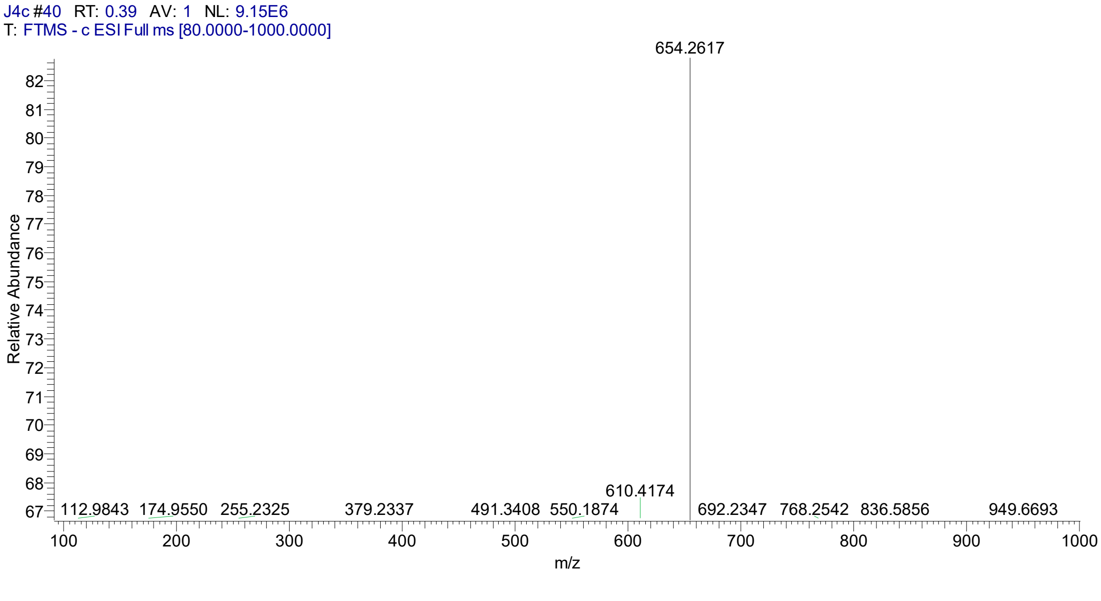

Figure S6.HR Mass spectrum (ESI) of compound **13b**.

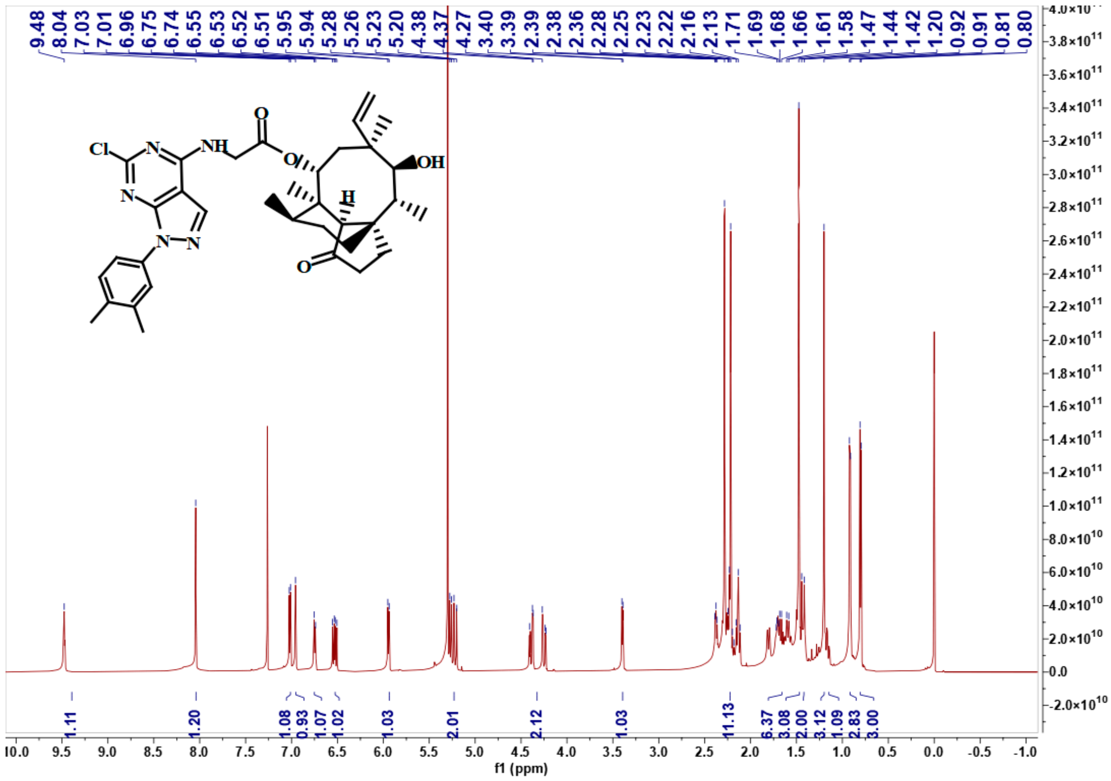

Figure S7.^{1}H-NMR spectrum (CDCl3, 600MHz) of compound **14b**.

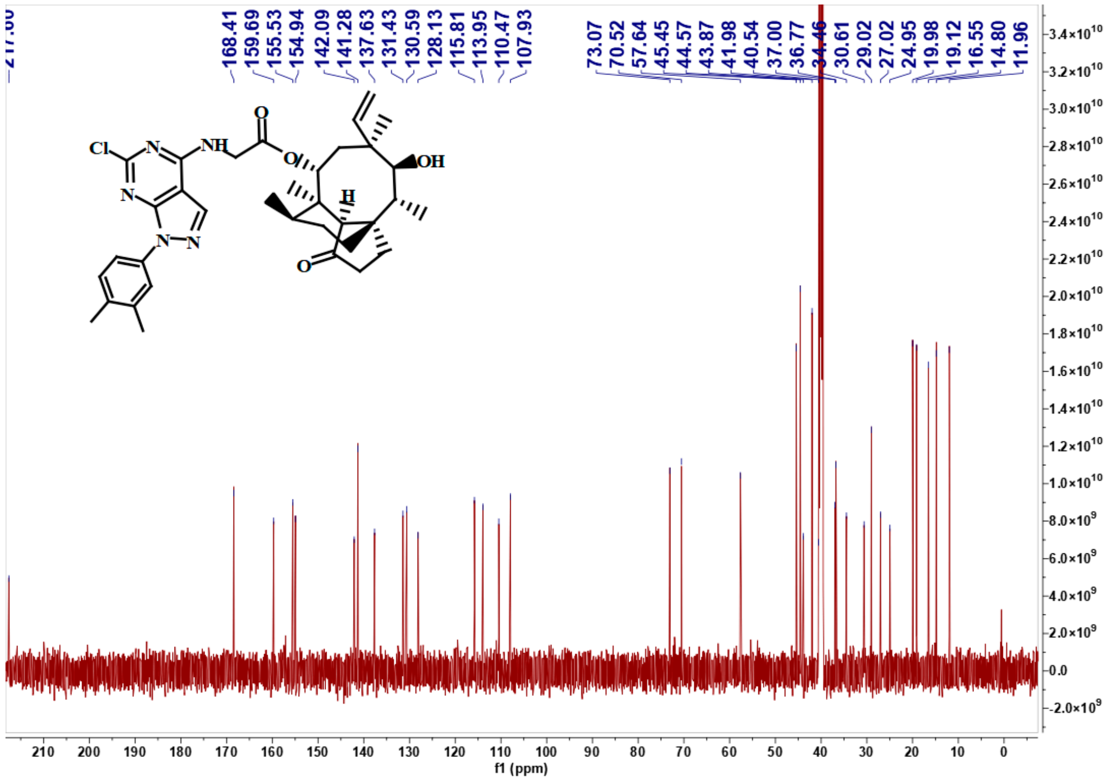

Figure S8.^{13}C-NMR spectrum (DMSO, 151MHz) of compound **14b**.

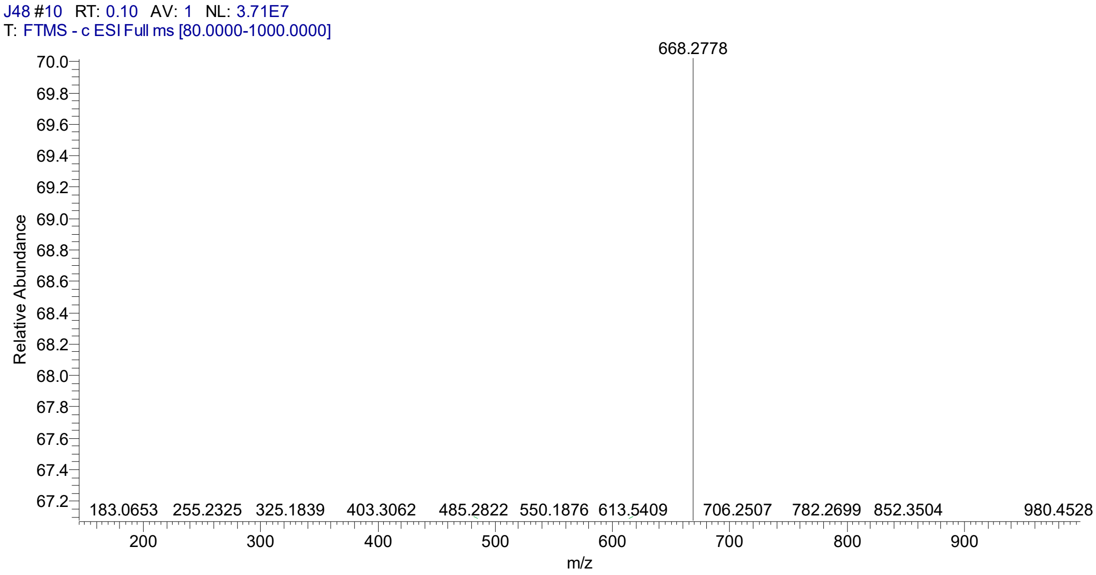

Figure S9.HR Mass spectrum (ESI) of compound **14b**.

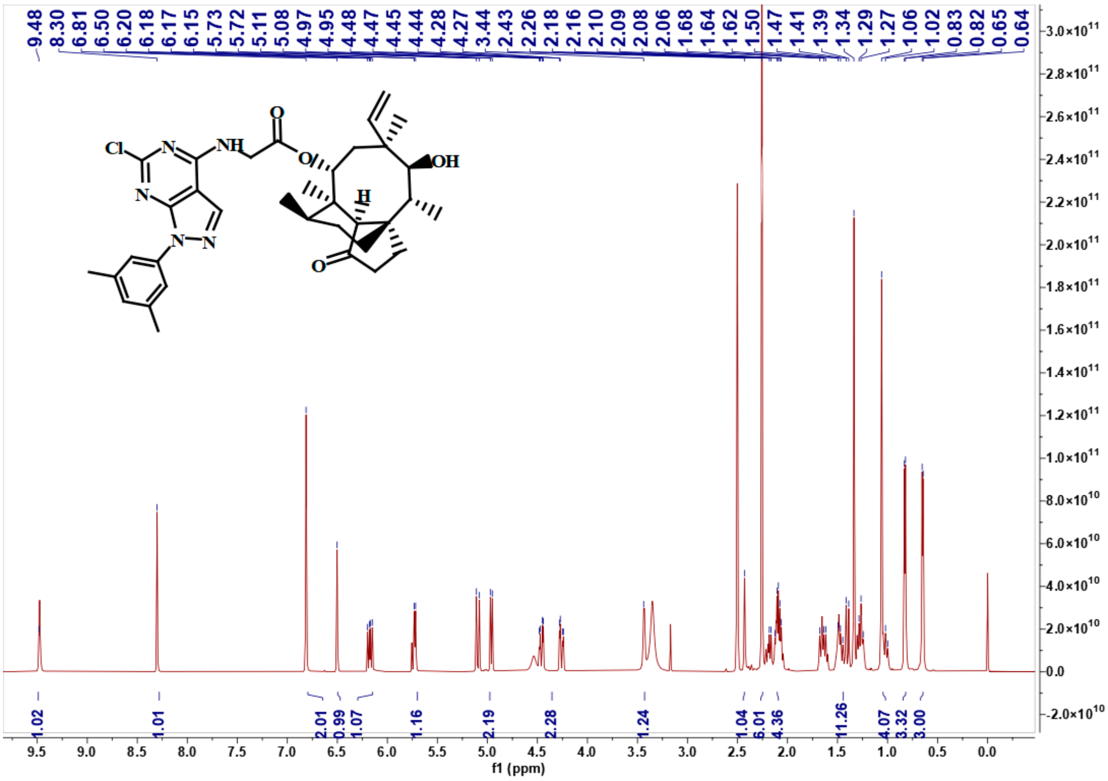

Figure S10.^{1}H-NMR spectrum ($CDCl_3$, 600MHz) of compound **15b**.

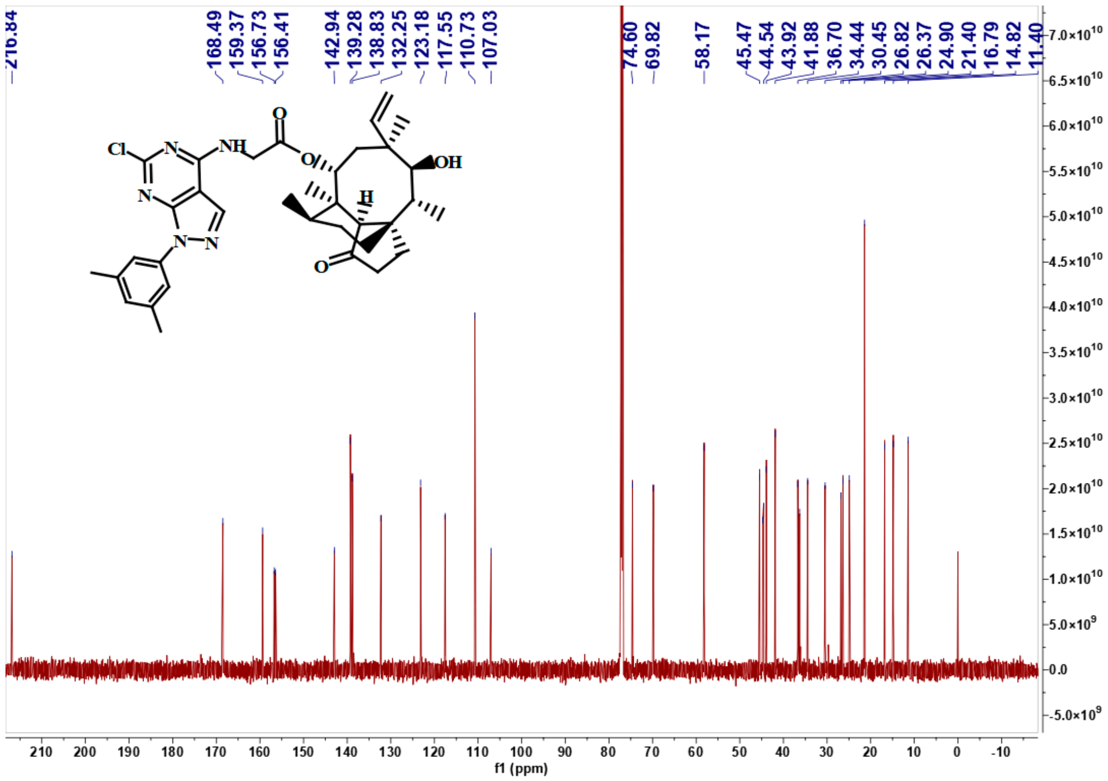

Figure S11.^{13}C-NMR spectrum ($CDCl_3$, 151MHz) of compound **15b**.

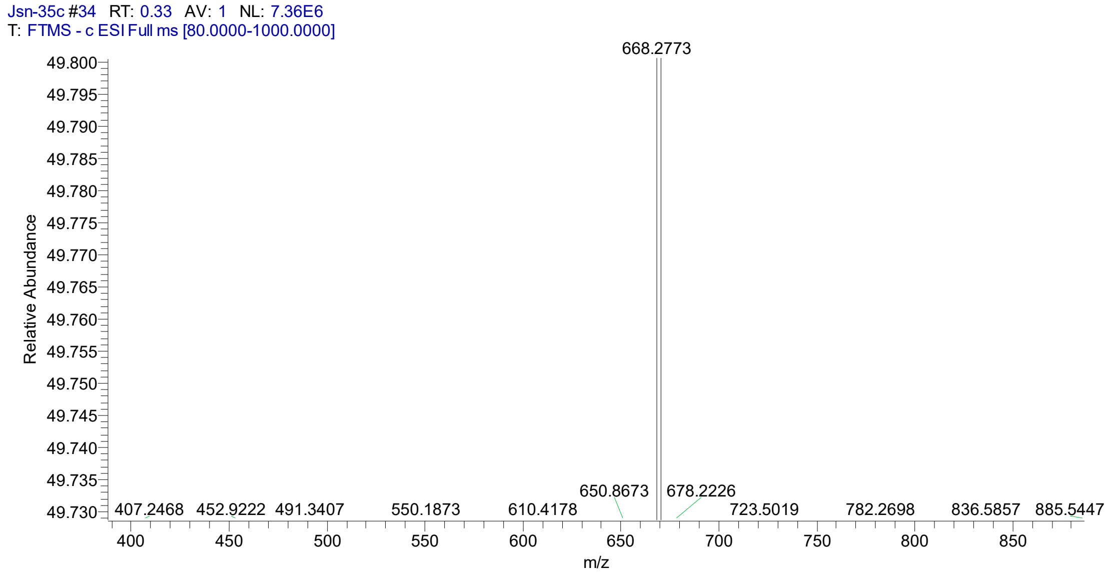

Figure S12.HR Mass spectrum (ESI) of compound **15b.**

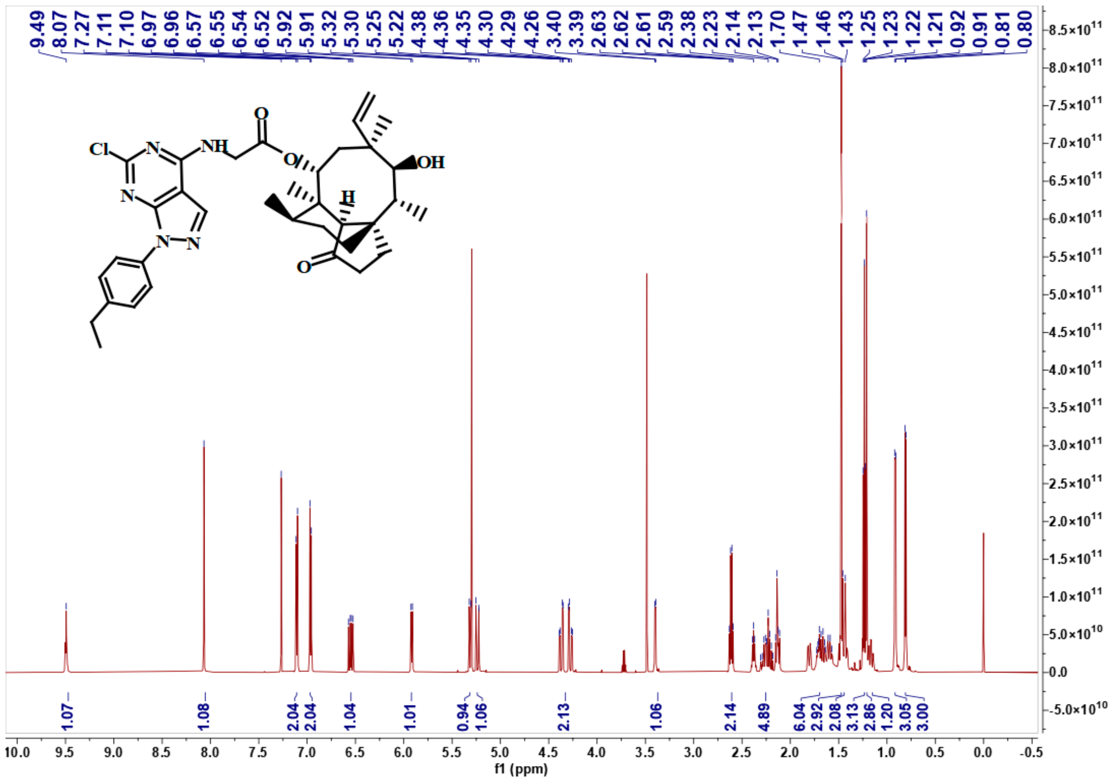

Figure S13.^{1}H-NMR spectrum ($CDCl_3$, 600MHz) of compound **16b**.

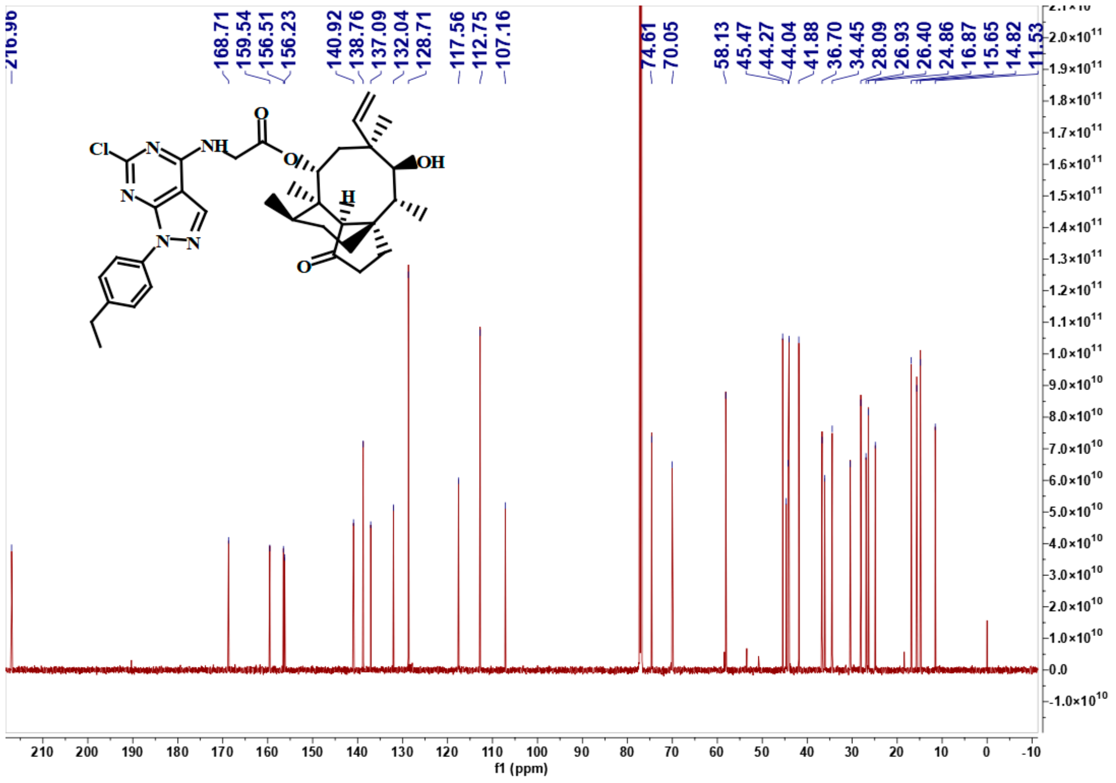

Figure S14.^{13}C-NMR spectrum ($CDCl_3$, 151MHz) of compound **16b**.

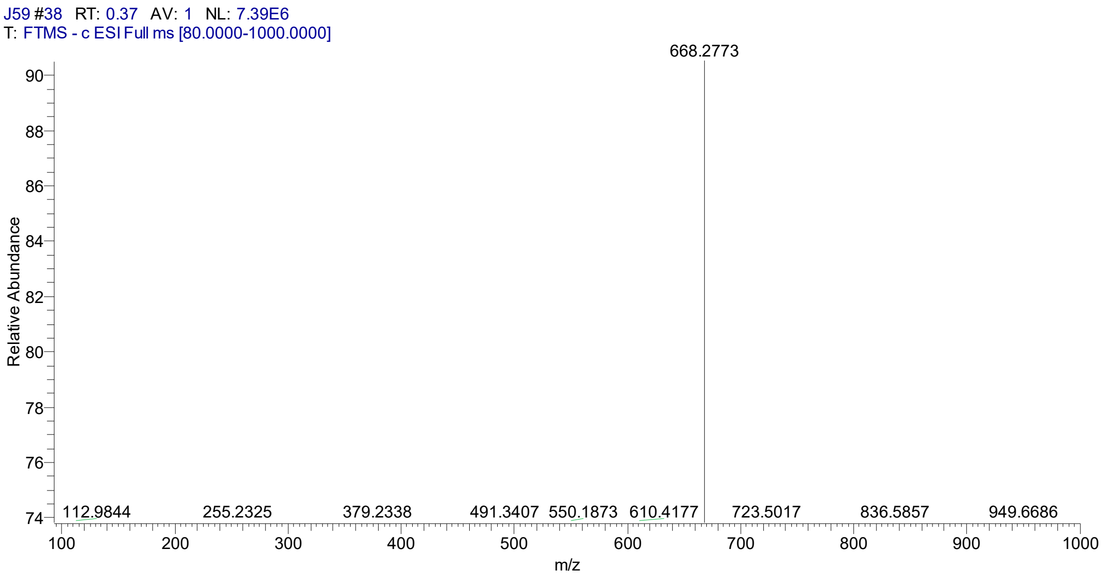

Figure S15.HR Mass spectrum (ESI) of compound **16b.**

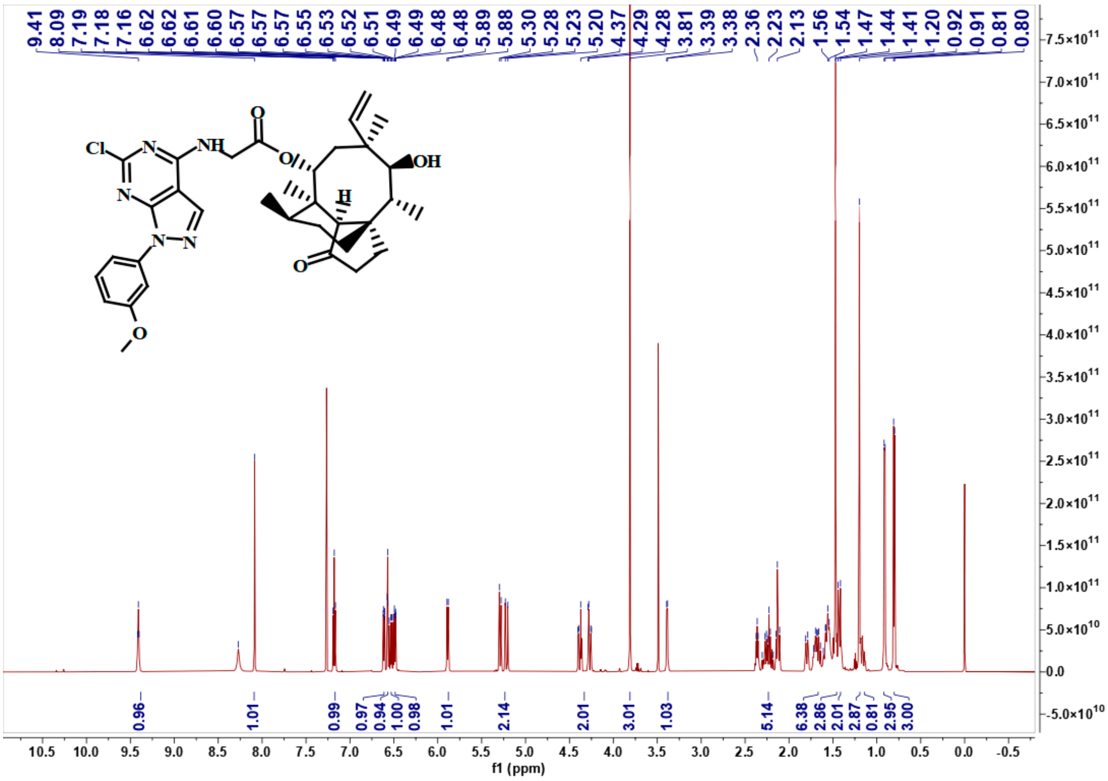

Figure S16.^{1}H-NMR spectrum ($CDCl_3$, 600MHz) of compound **17b**.

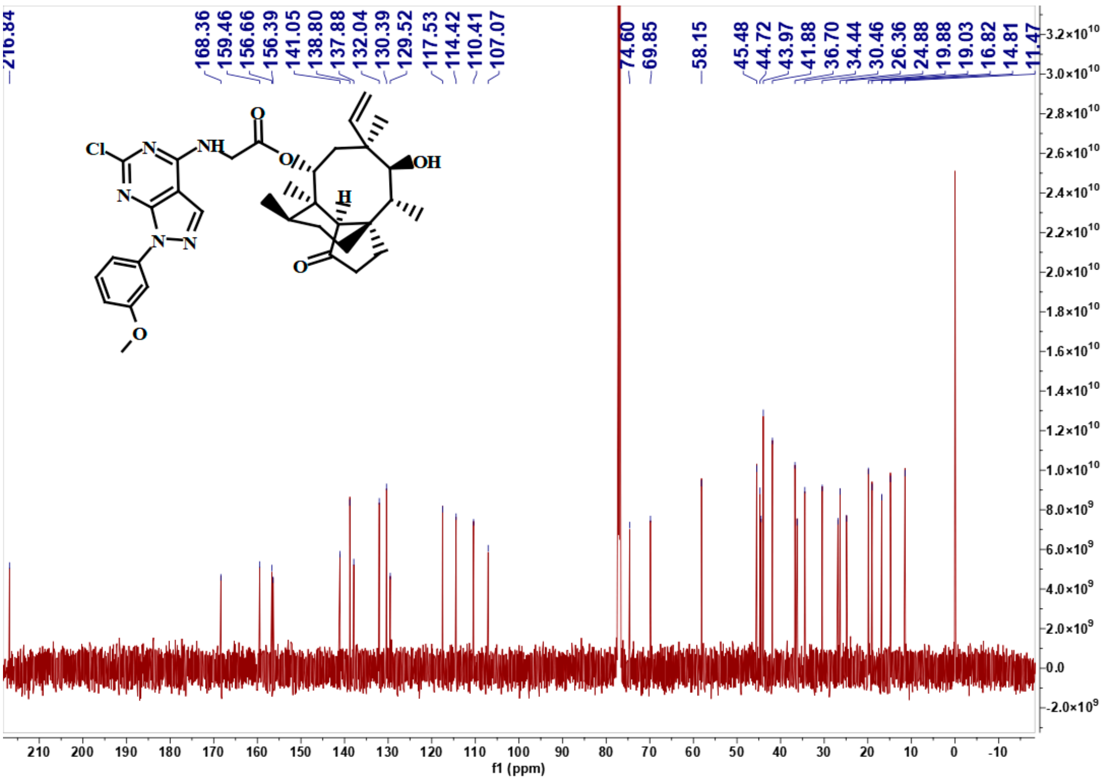

Figure S17.^{13}C-NMR spectrum ($CDCl_3$, 151MHz) of compound **17b**.

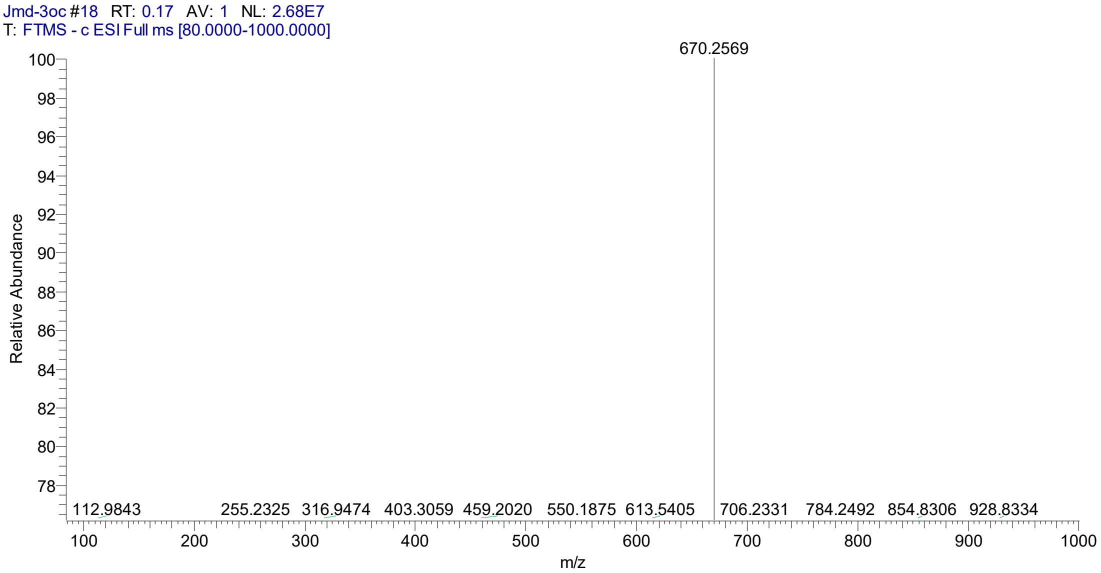

Figure S18.HR Mass spectrum (ESI) of compound **17b**.

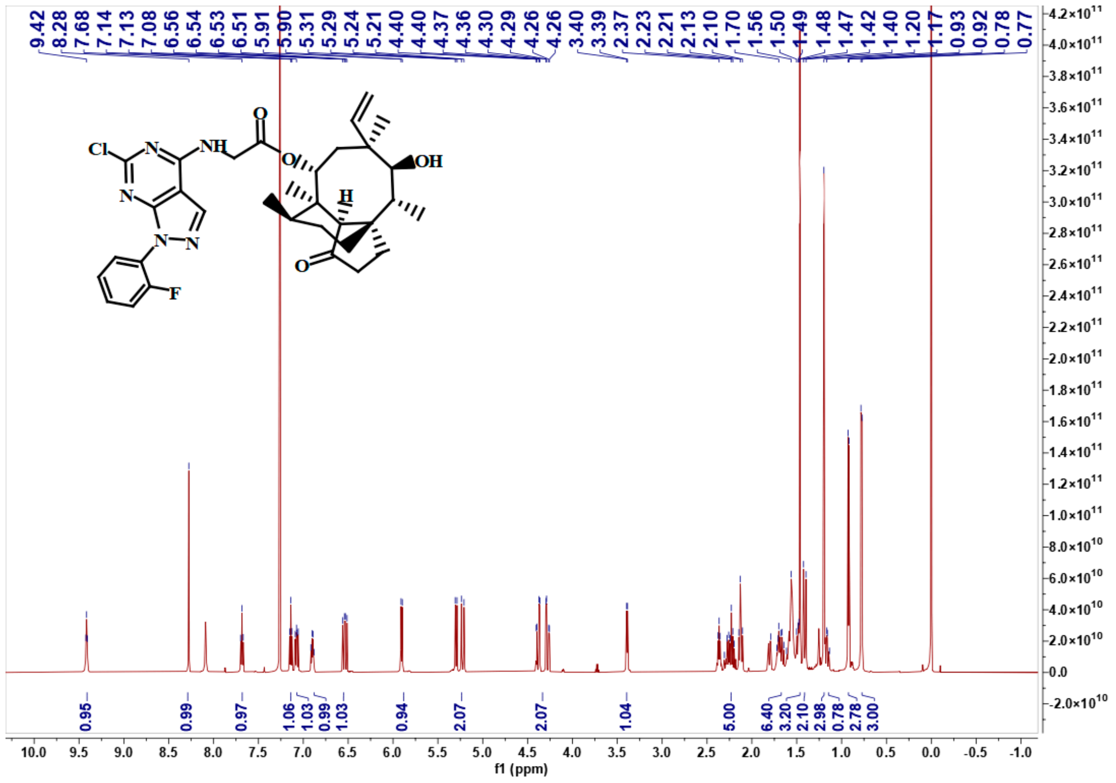

Figure S19.^{1}H-NMR spectrum ($CDCl_3$, 600MHz) of compound **18b**.

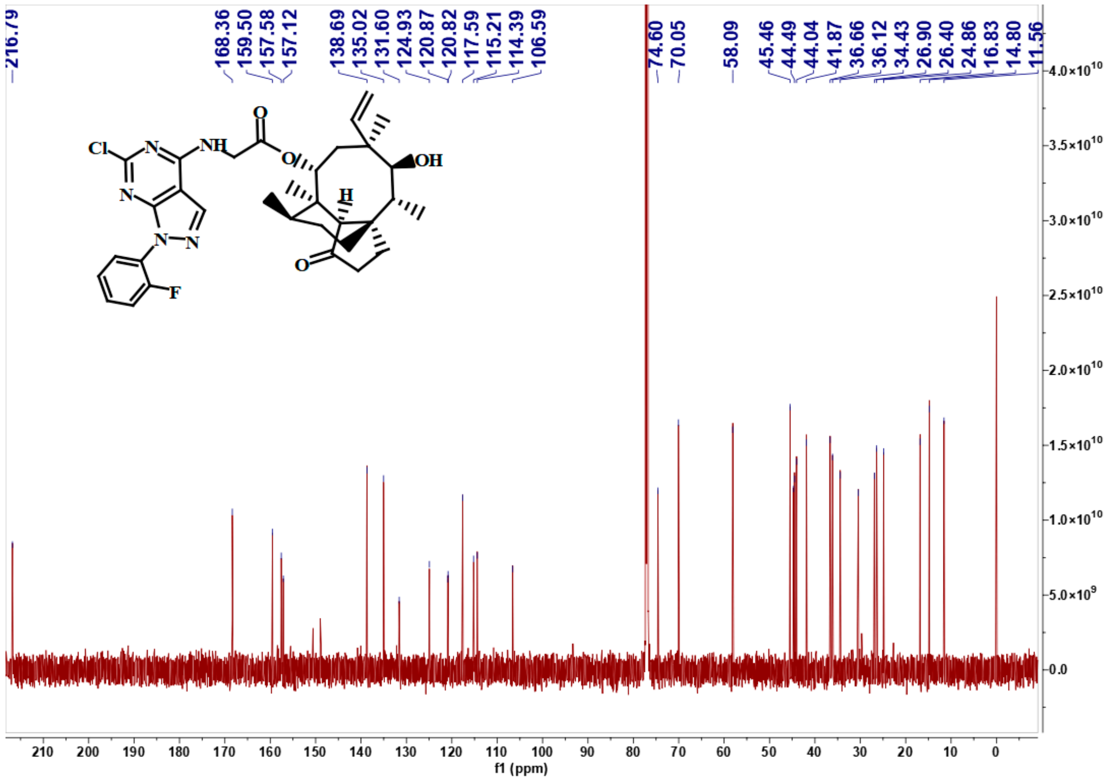

Figure S20.^{13}C-NMR spectrum ($CDCl_3$, 151MHz) of compound **18b**.

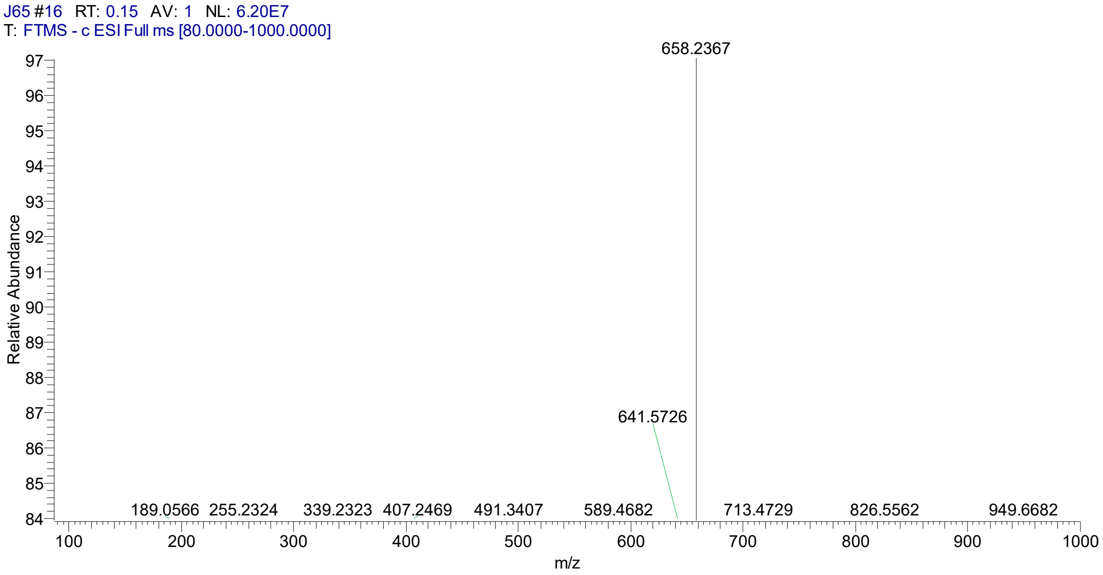

Figure S21.HR Mass spectrum (ESI) of compound **18b**.

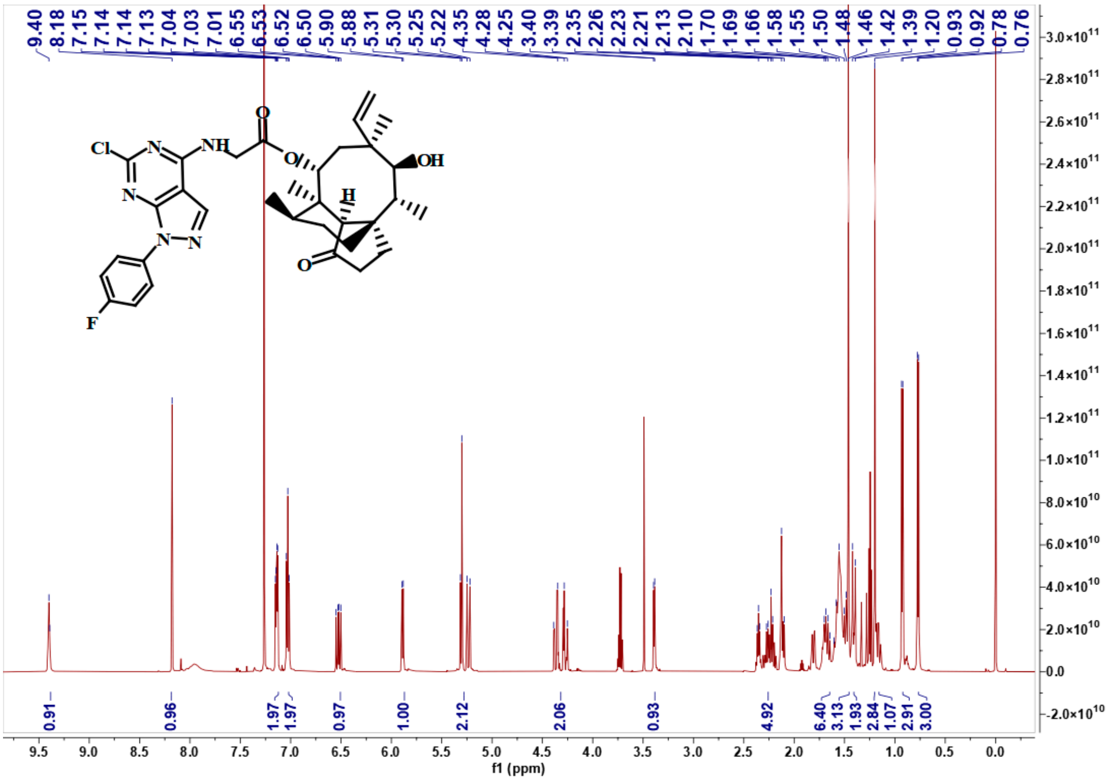

Figure S22.^{1}H-NMR spectrum ($CDCl_3$, 600MHz) of compound **19b**.

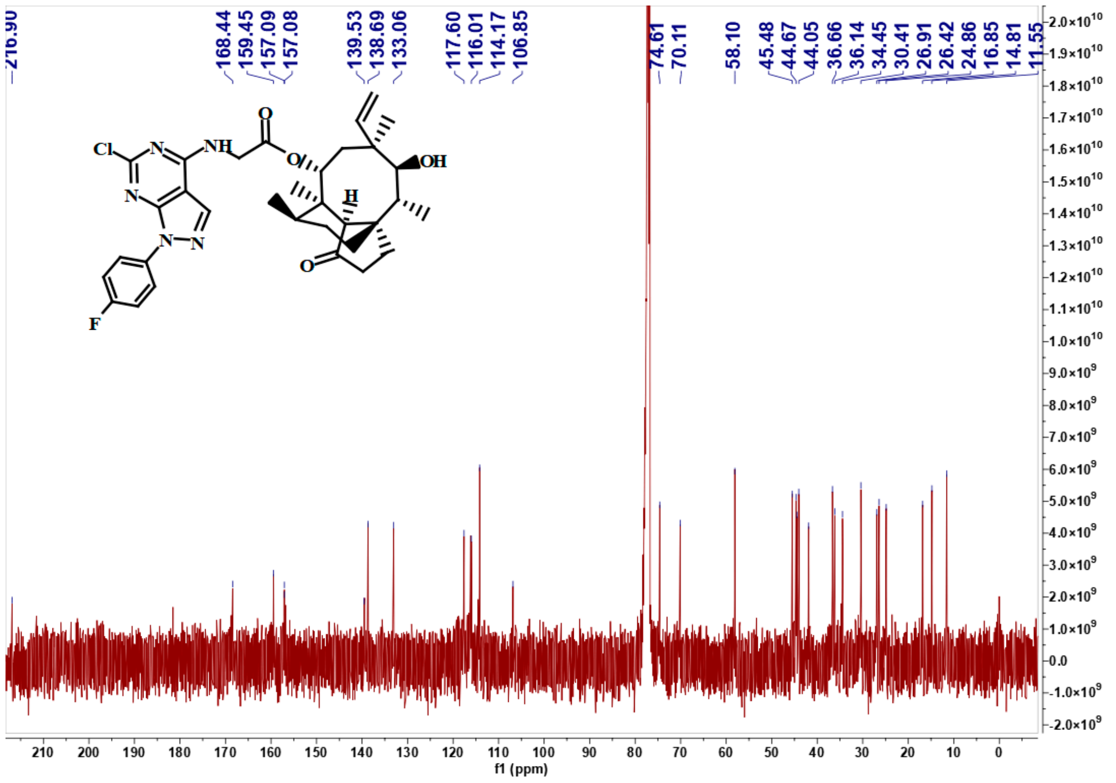

Figure S23.^{13}C-NMR spectrum ($CDCl_3$, 151MHz) of compound **19b**.

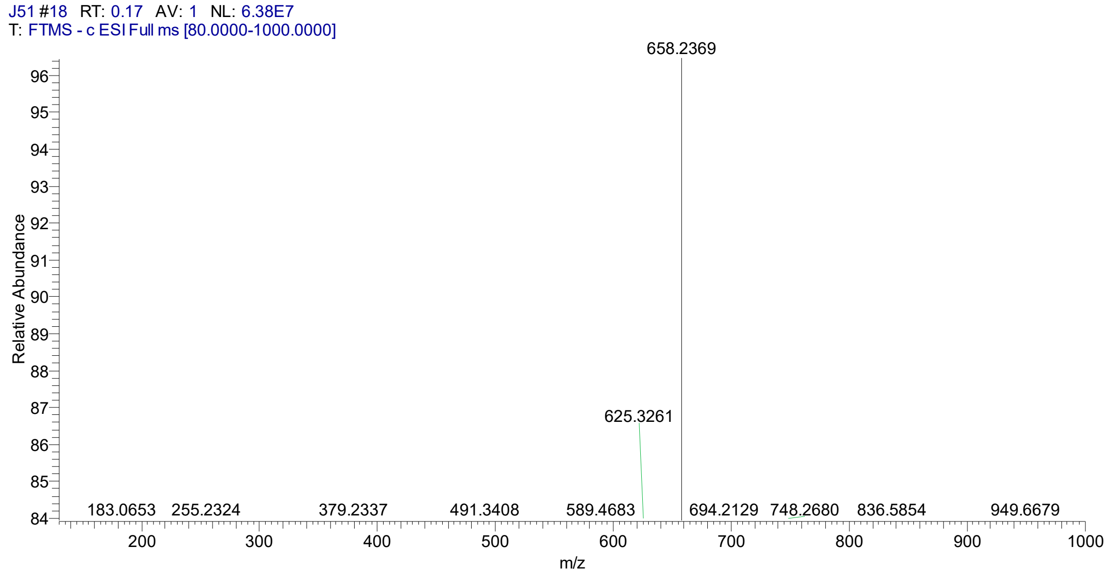

Figure S24.HR Mass spectrum (ESI) of compound **19b**.

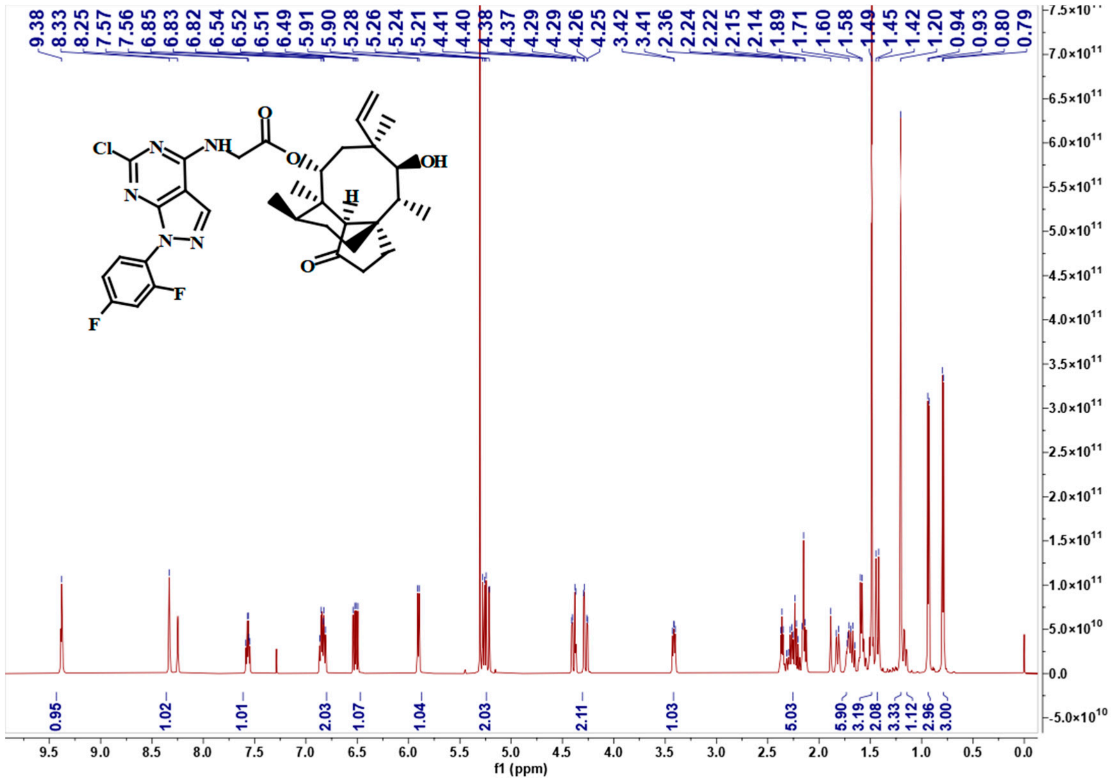

Figure S25.^{1}H-NMR spectrum ($CDCl_3$, 600MHz) of compound **20b**.

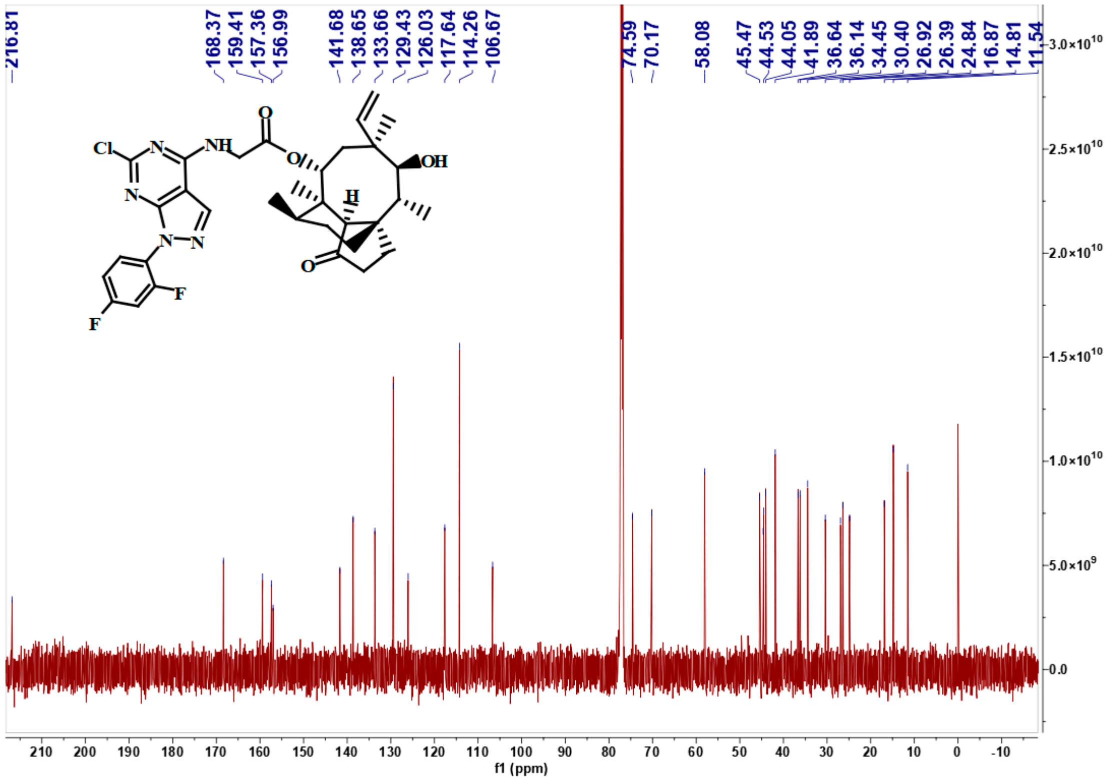

Figure S26.^{13}C-NMR spectrum (DMSO, 151MHz) of compound **20b**.

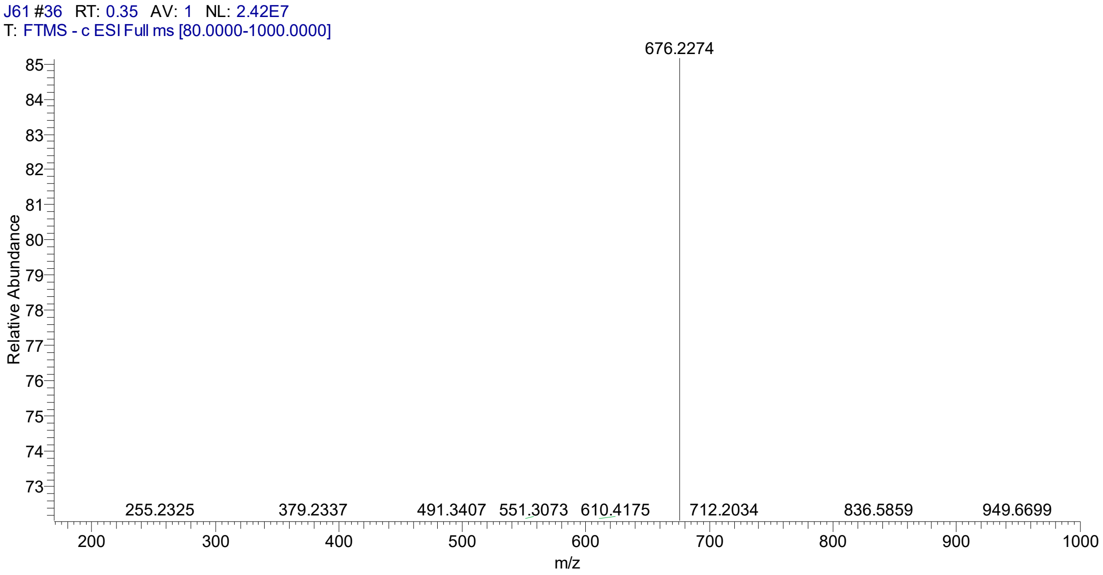

Figure S27.HR Mass spectrum (ESI) of compound **20b**.

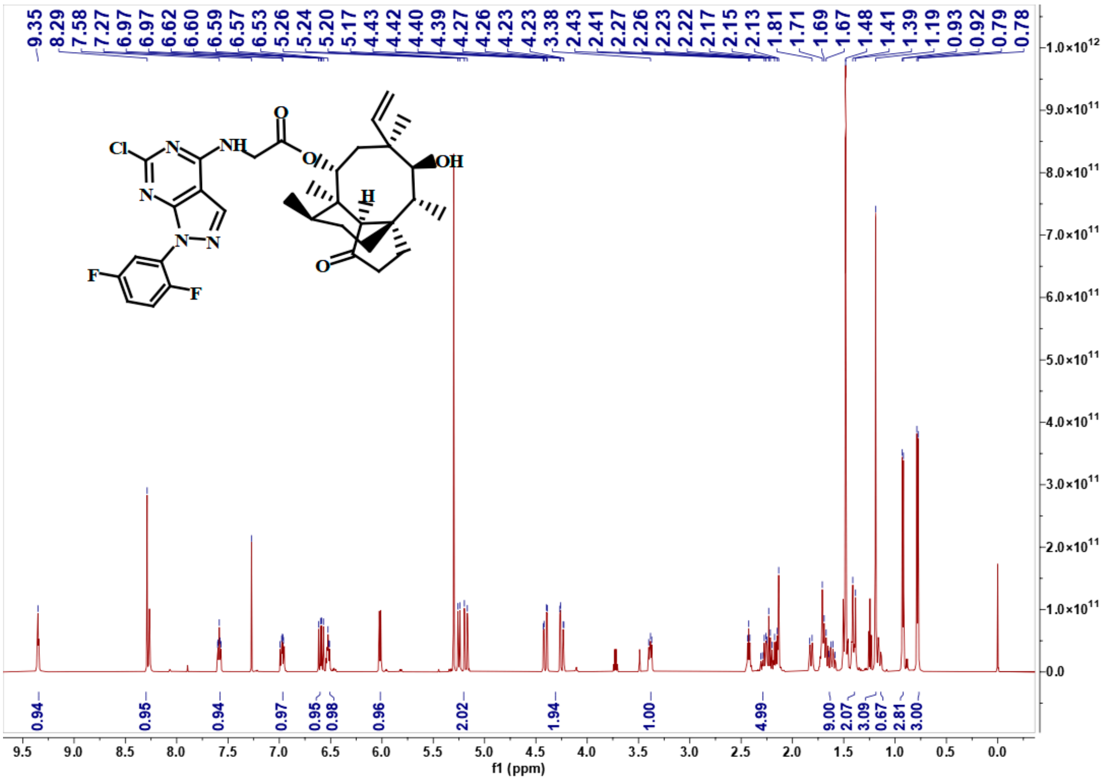

Figure S28.^{1}H-NMR spectrum ($CDCl_3$, 600MHz) of compound **21b**.

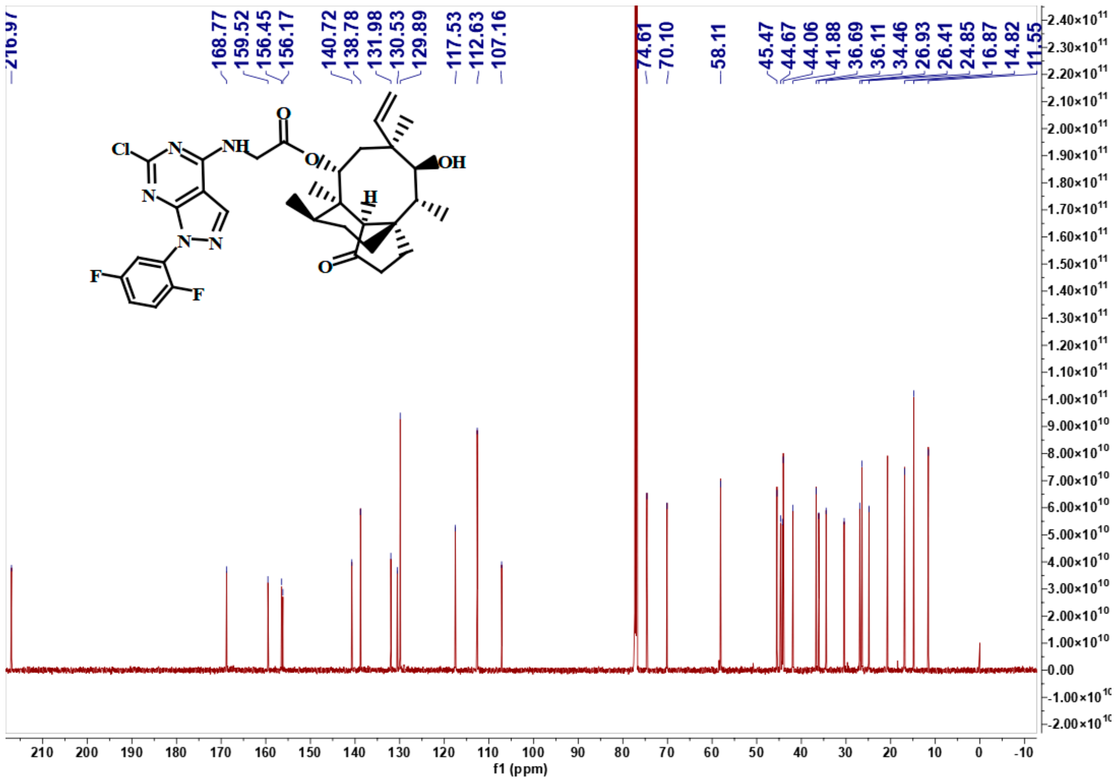

Figure S29.^{13}C-NMR spectrum ($CDCl_3$, 151MHz) of compound **21b**.

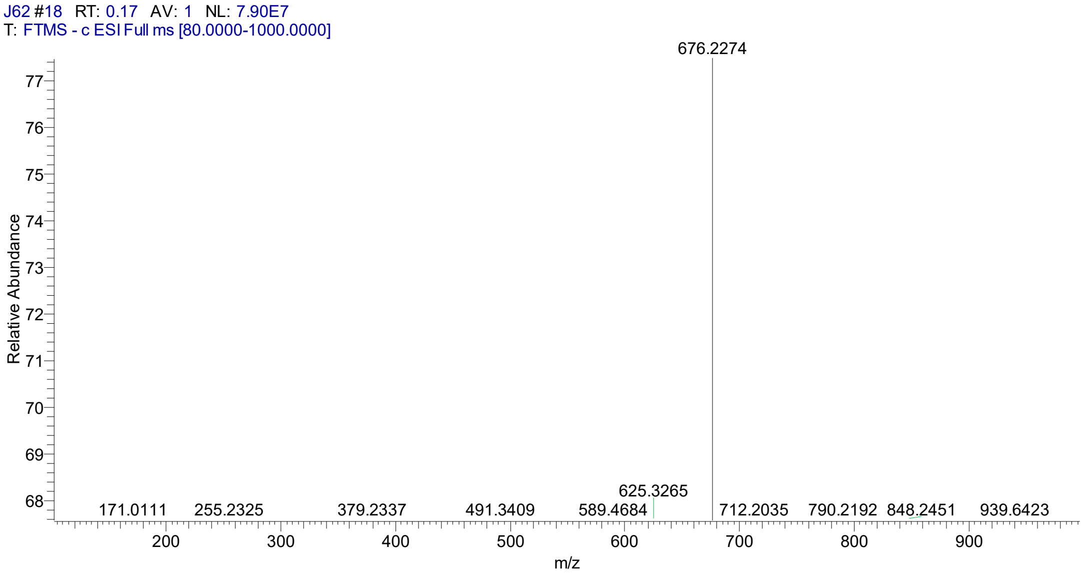

Figure S30.HR Mass spectrum (ESI) of compound **21b**.

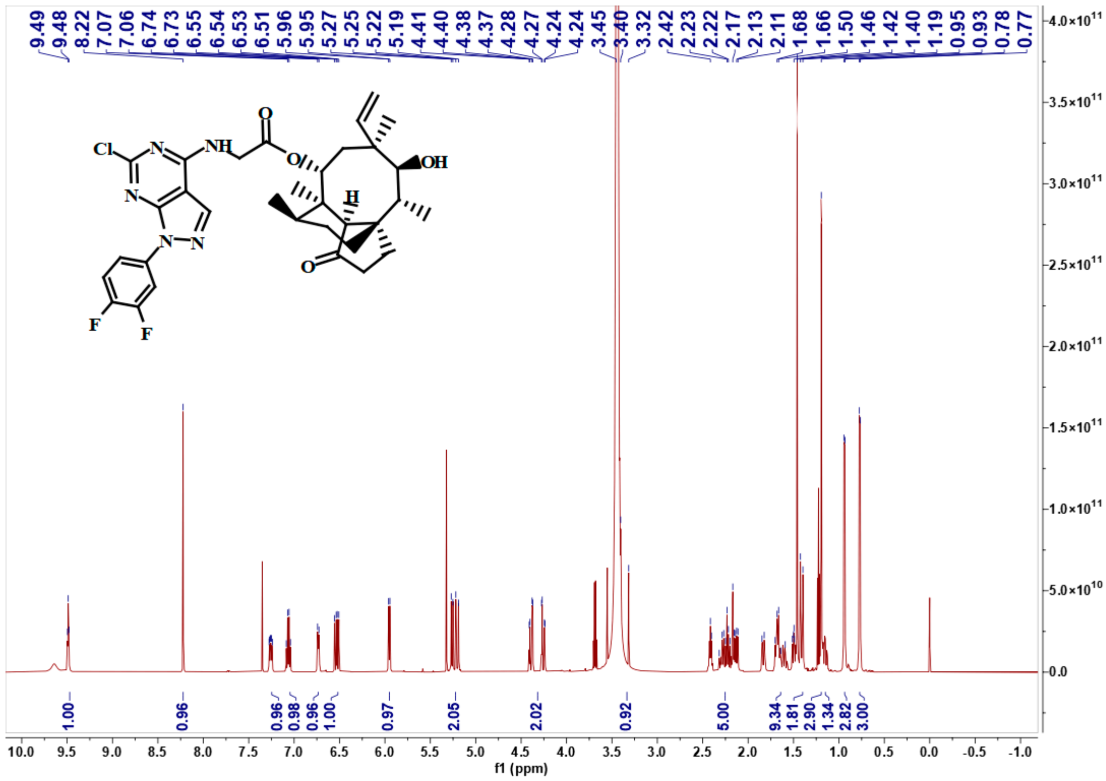

Figure S31.^{1}H-NMR spectrum ($CDCl_3$, 600MHz) of compound **22b**.

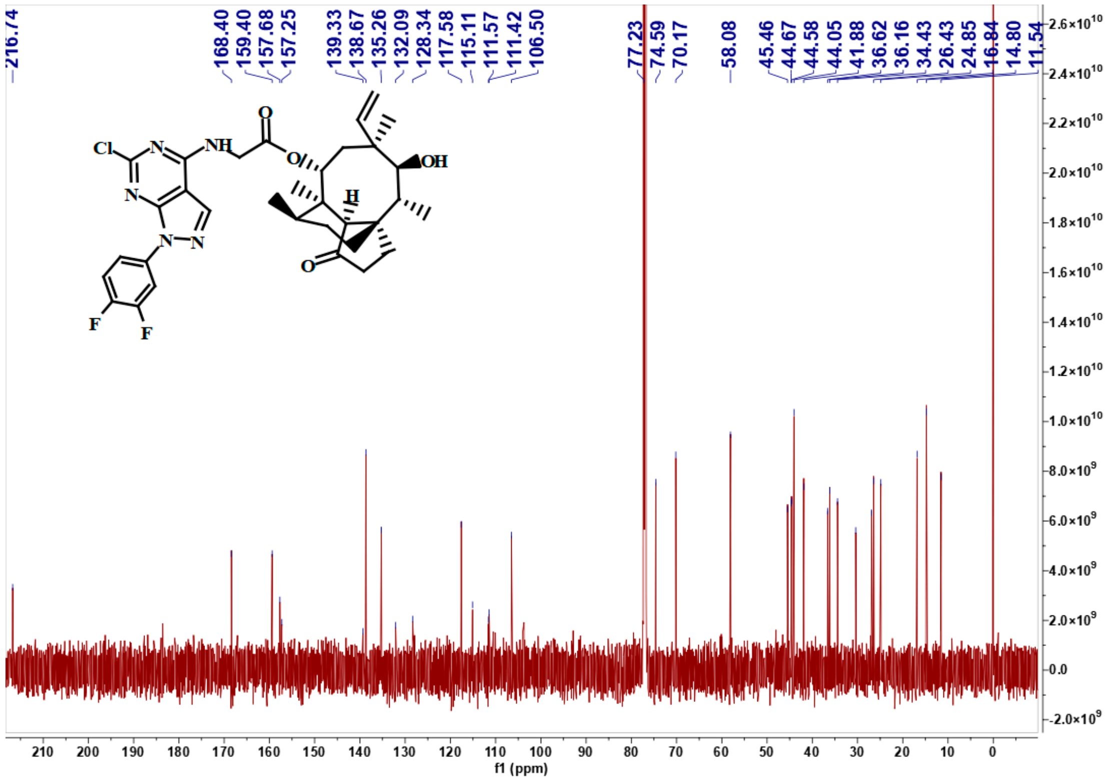

Figure S32.^{13}C-NMR spectrum ($CDCl_3$, 151MHz) of compound **22b**.

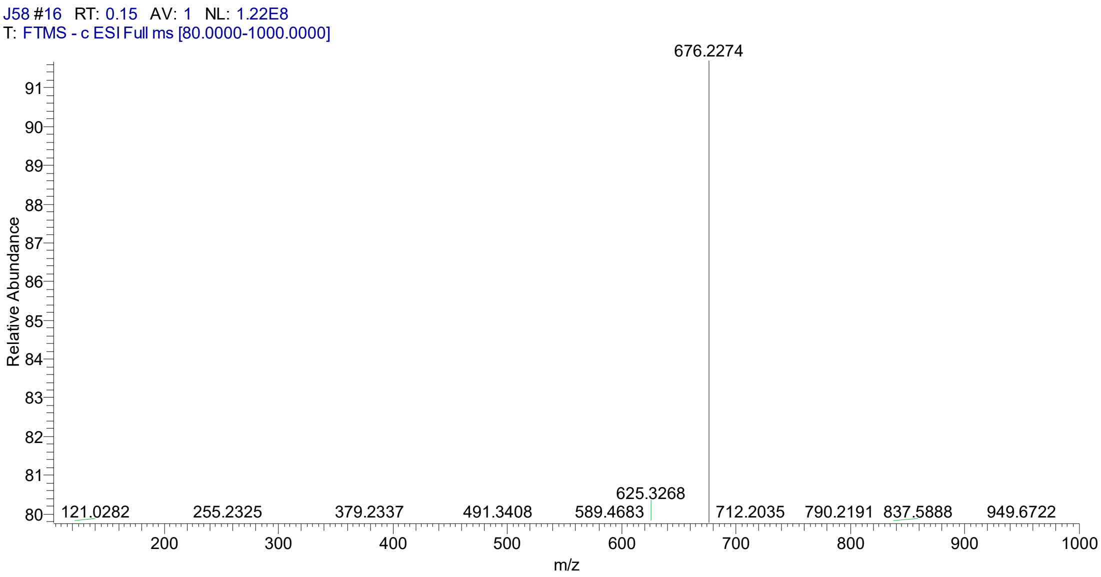

Figure S33.HR Mass spectrum (ESI) of compound **22b**.

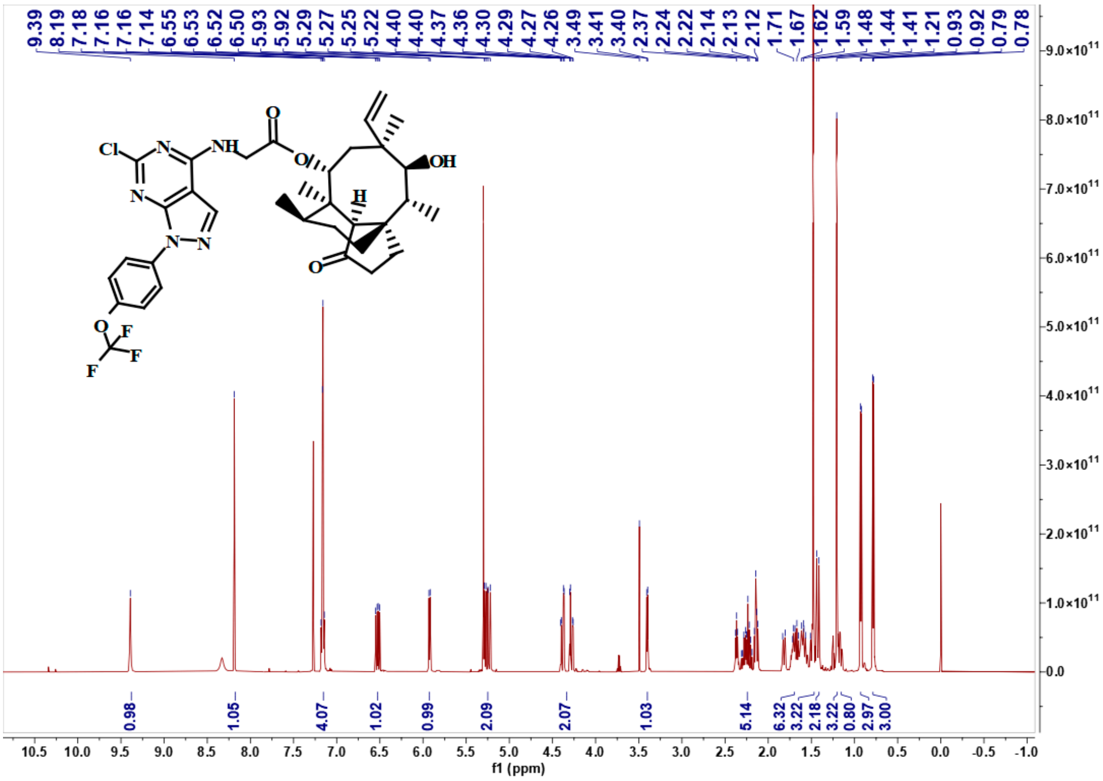

Figure S34.^{1}H-NMR spectrum ($CDCl_3$, 600MHz) of compound **23b**.

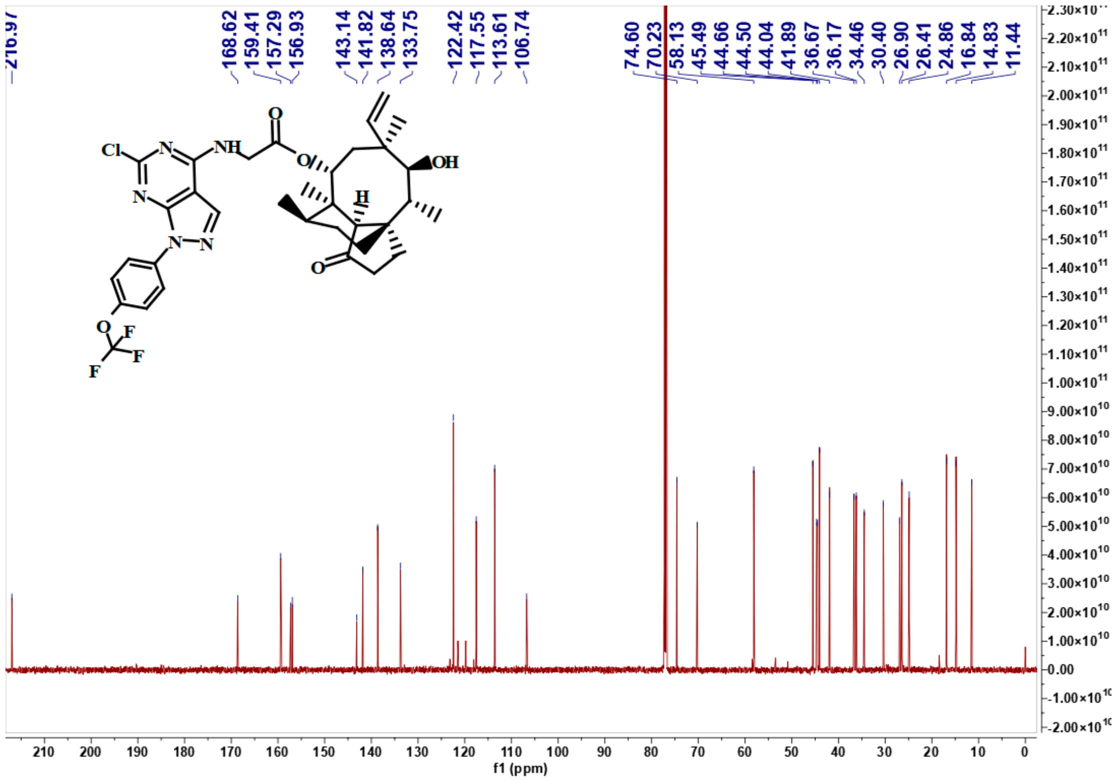

Figure S35.^{13}C-NMR spectrum ($CDCl_3$, 151MHz) of compound **23b**.

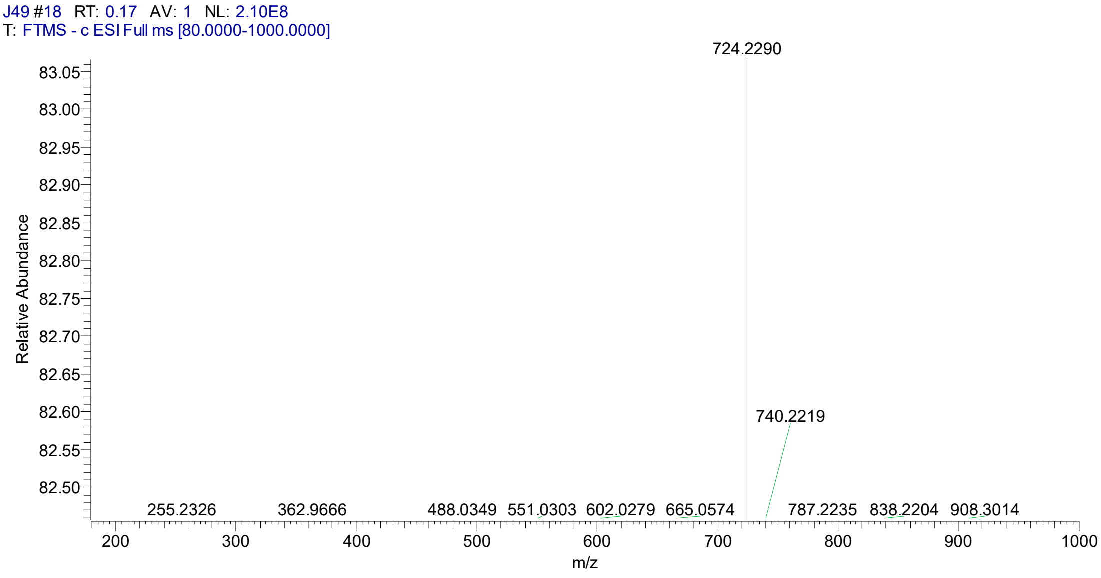

Figure S36.HR Mass spectrum (ESI) of compound **23b**.

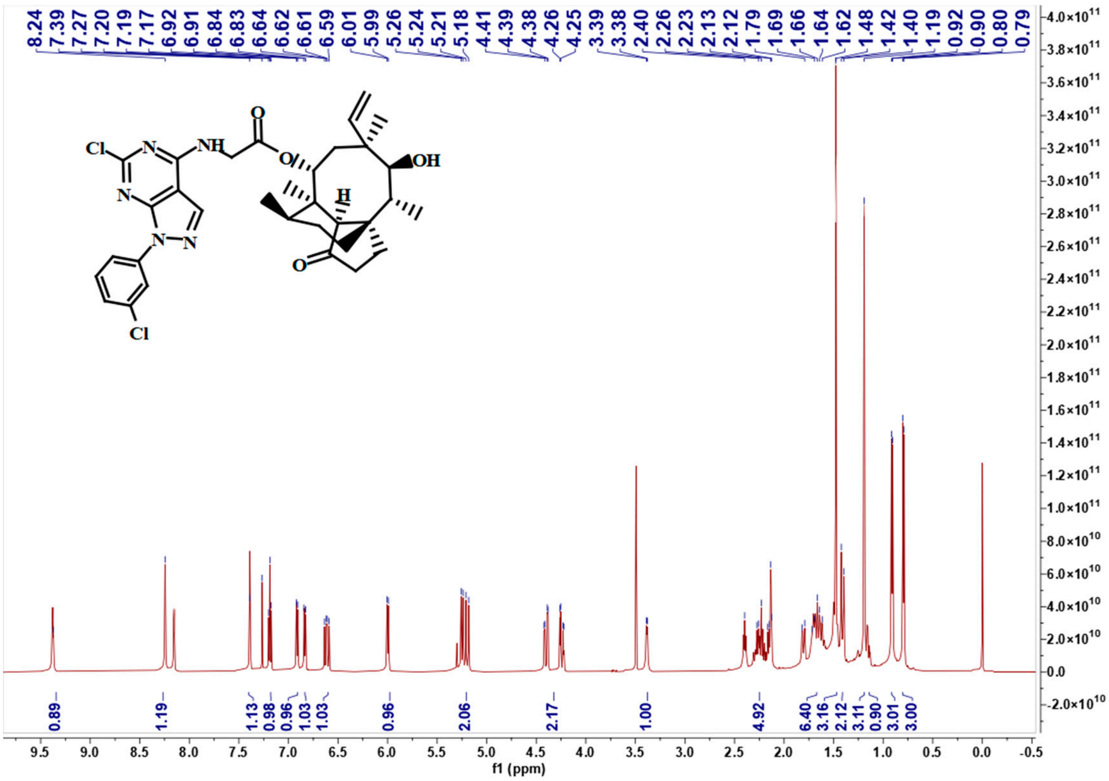

Figure S37.^{1}H-NMR spectrum ($CDCl_3$, 600MHz) of compound **24b**.

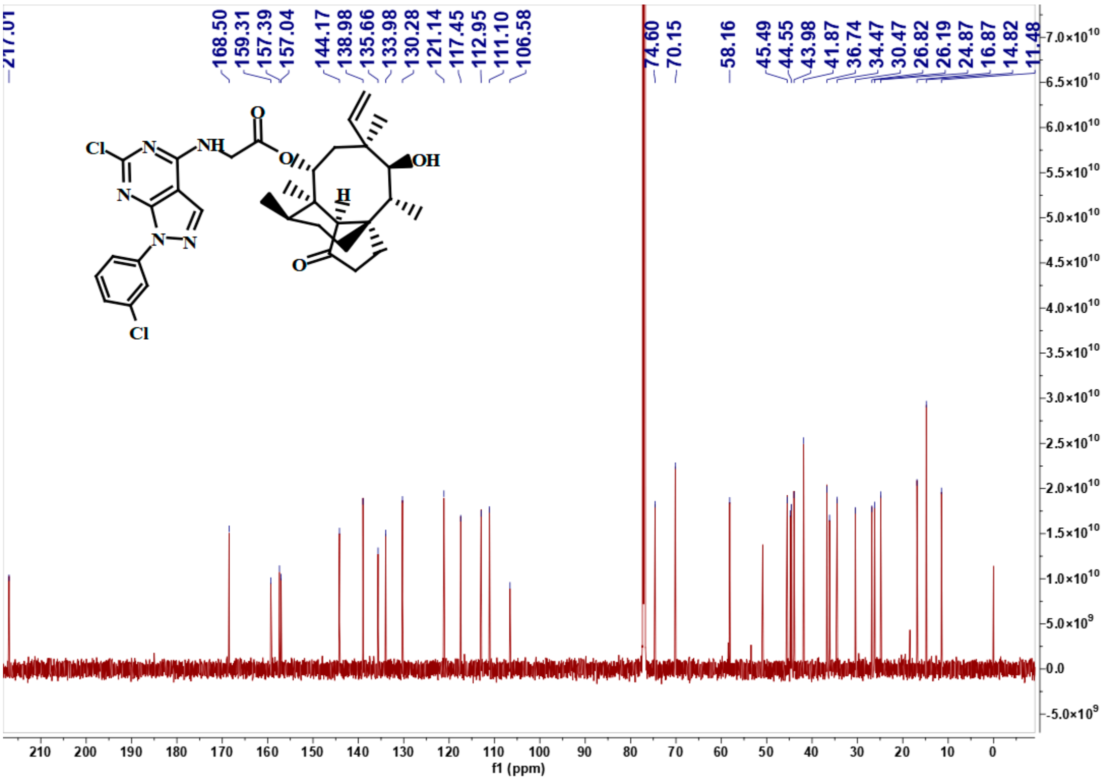

Figure S38.^{13}C-NMR spectrum ($CDCl_3$, 151MHz) of compound **24b**.

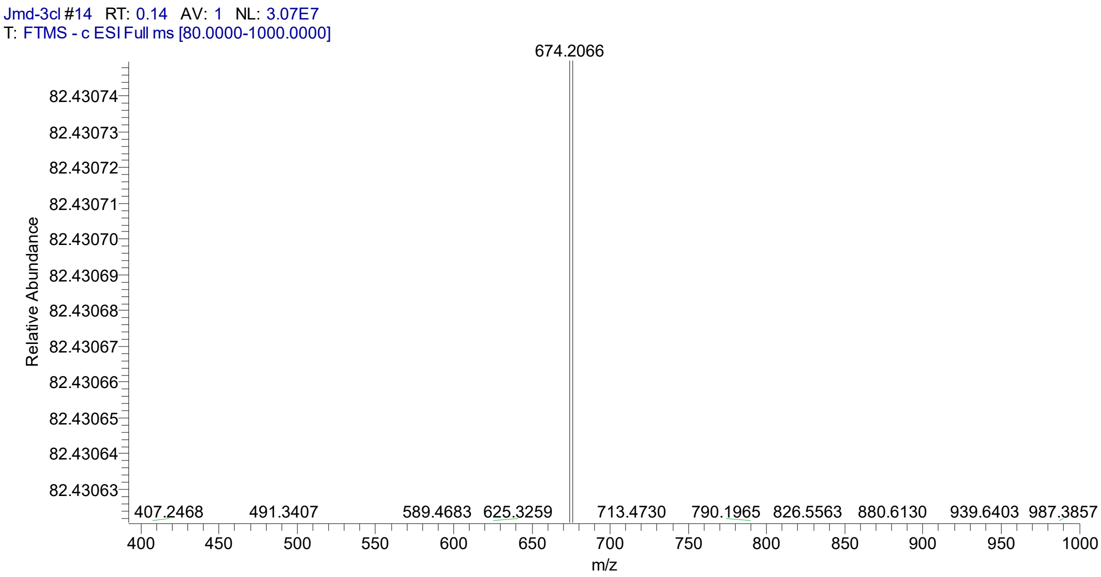

Figure S39. HR Mass spectrum (ESI) of compound **24b**.

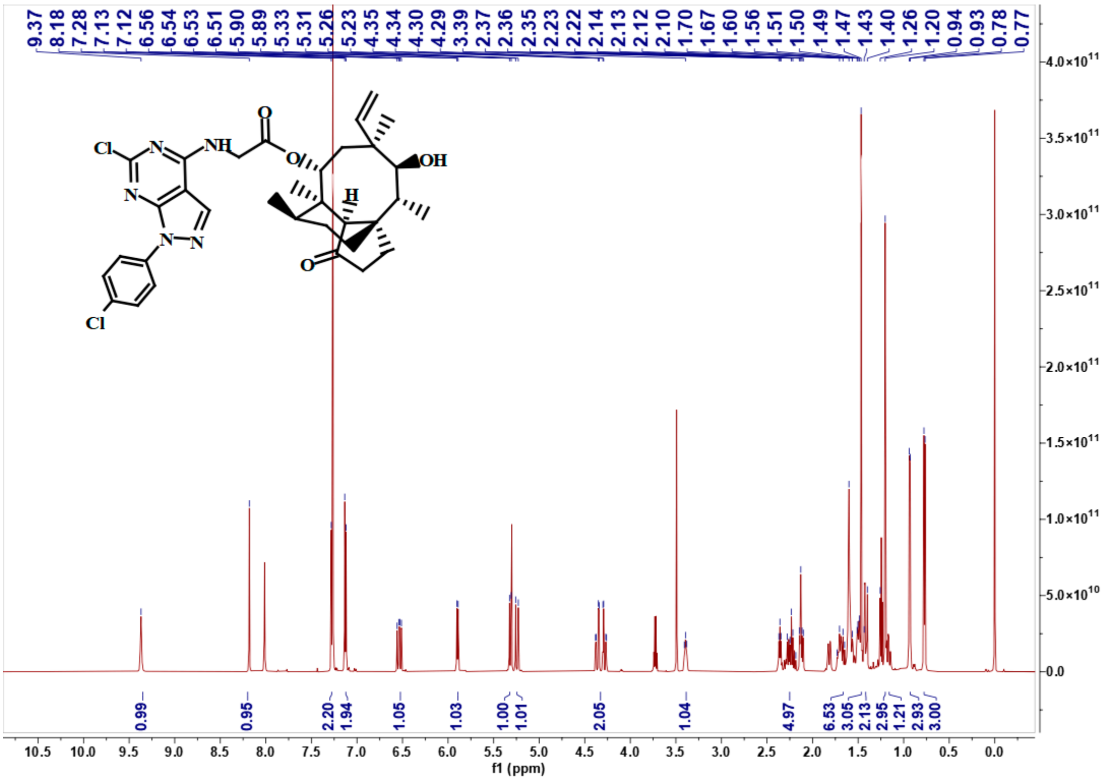

Figure S40. ^{1}H-NMR spectrum ($CDCl_3$, 600MHz) of compound **25b**.

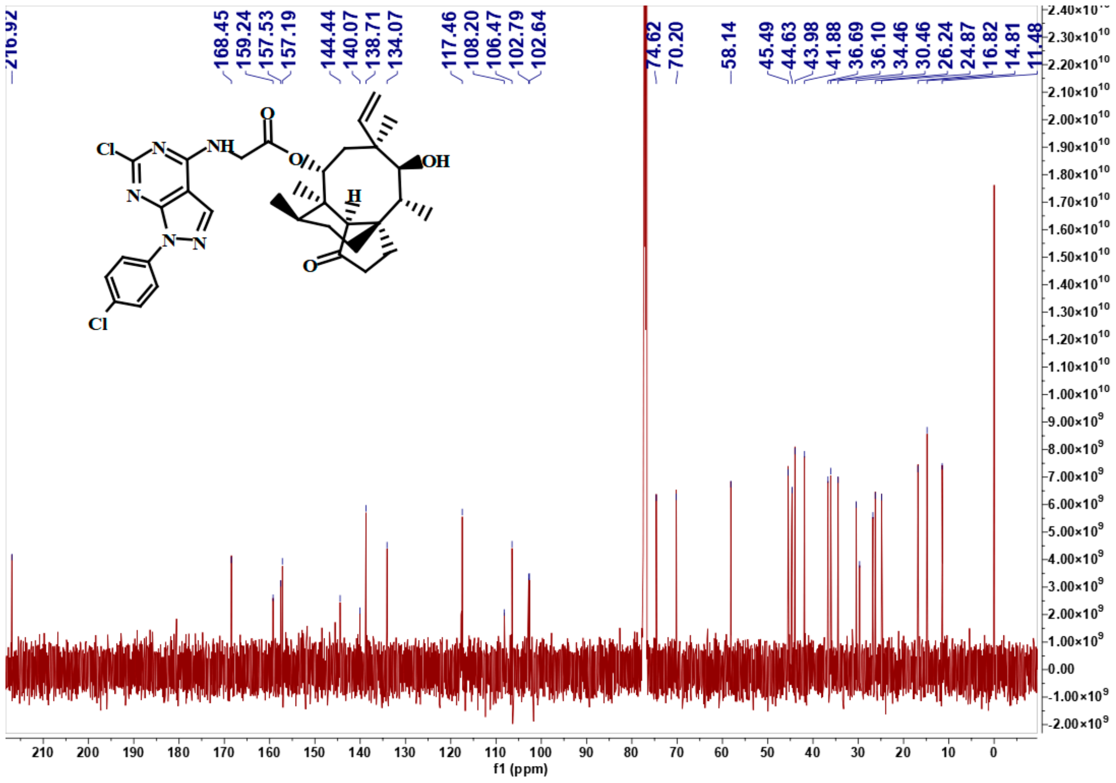

Figure S41.^{13}C-NMR spectrum ($CDCl_3$, 151MHz) of compound **25b**.

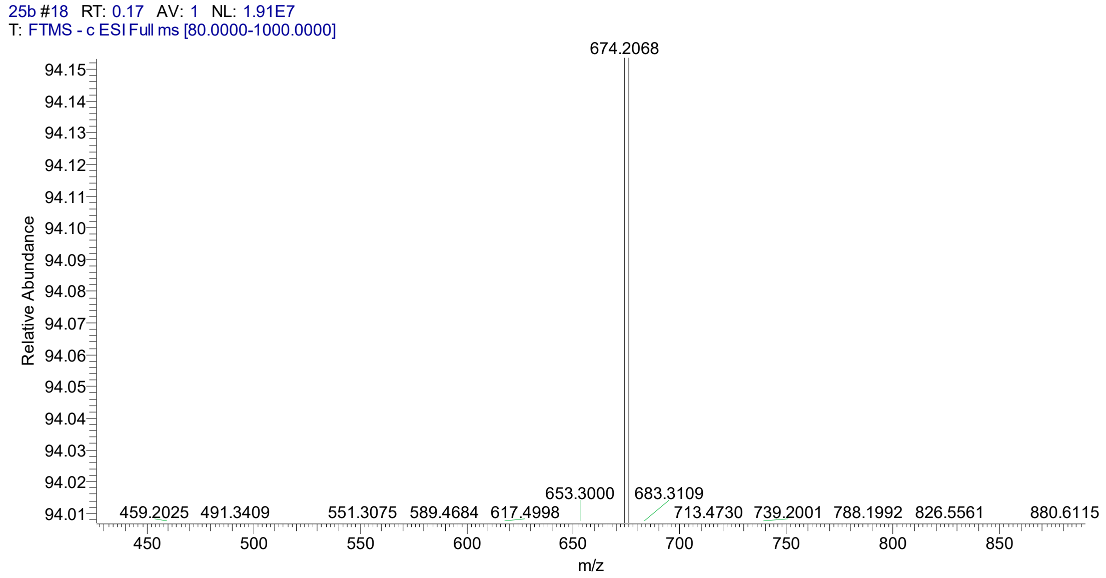

Figure S42.HR Mass spectrum (ESI) of compound **25b**.

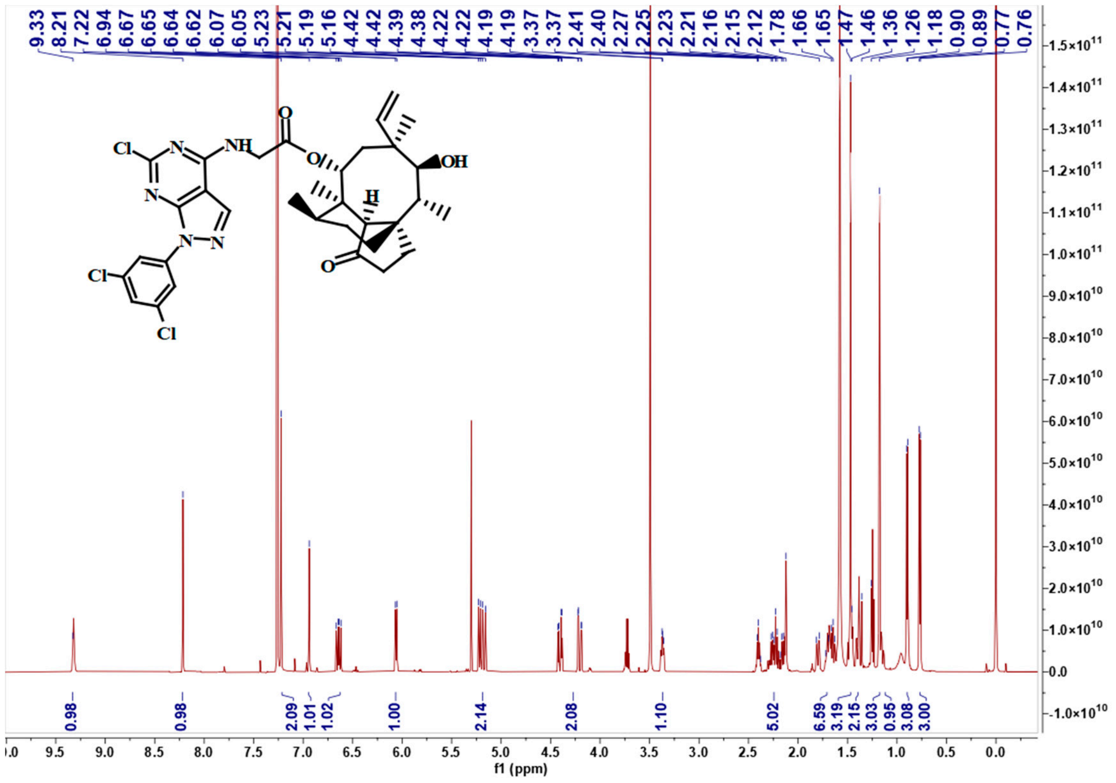

Figure S43.^{1}H-NMR spectrum ($CHCl_3$, 600MHz) of compound **26b**.

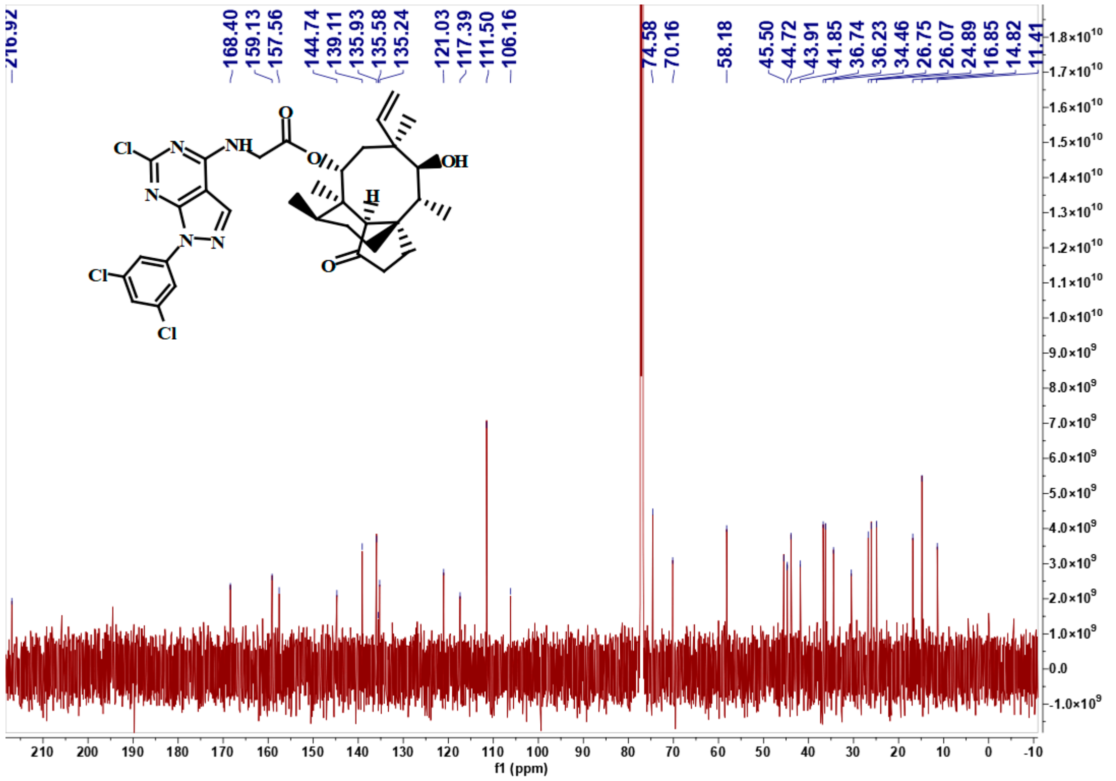

Figure S44.^{13}C-NMR spectrum ($CHCl_3$, 151MHz) of compound **26b**.

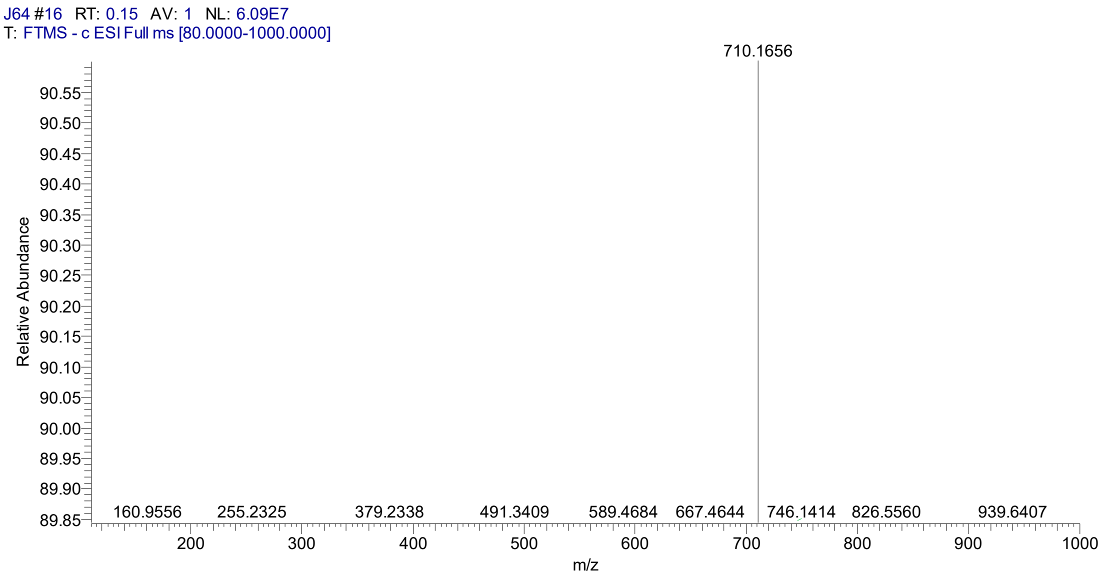

Figure S45.HR Mass spectrum (ESI) of compound **26b**.

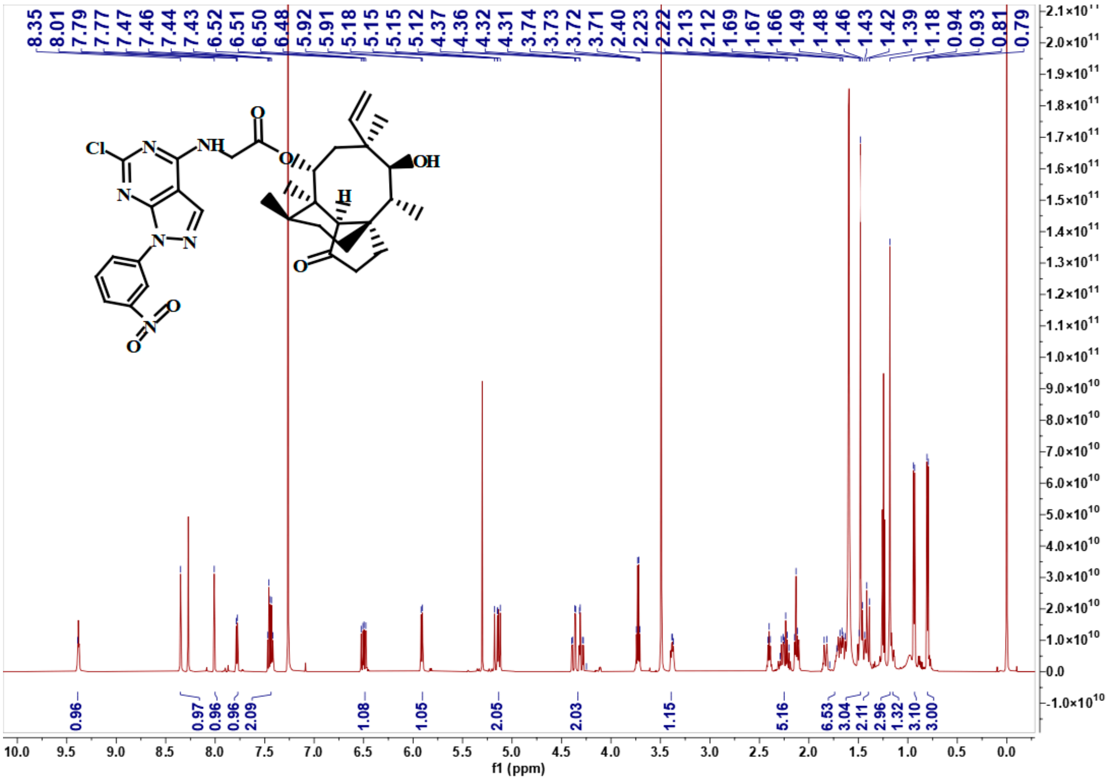

Figure S46.^{1}H-NMR spectrum ($CDCl_3$, 600MHz) of compound **27b**.

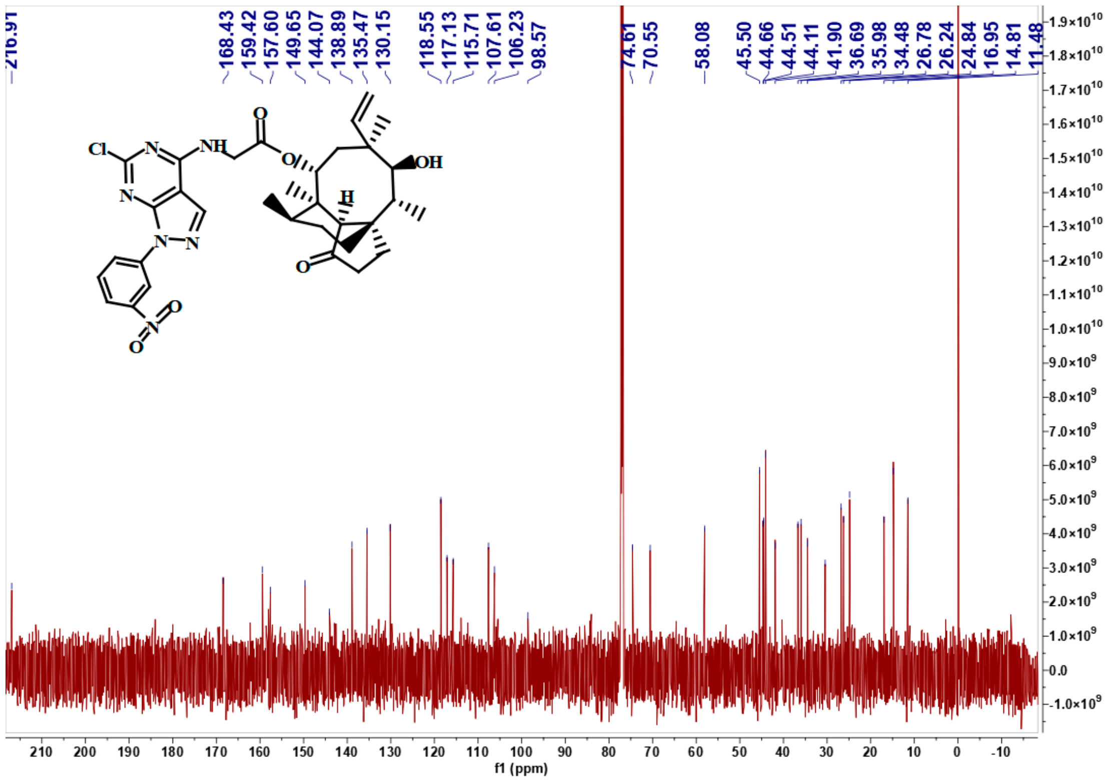

Figure S47.^{13}C-NMR spectrum ($CDCl_3$, 151MHz) of compound **27b**.

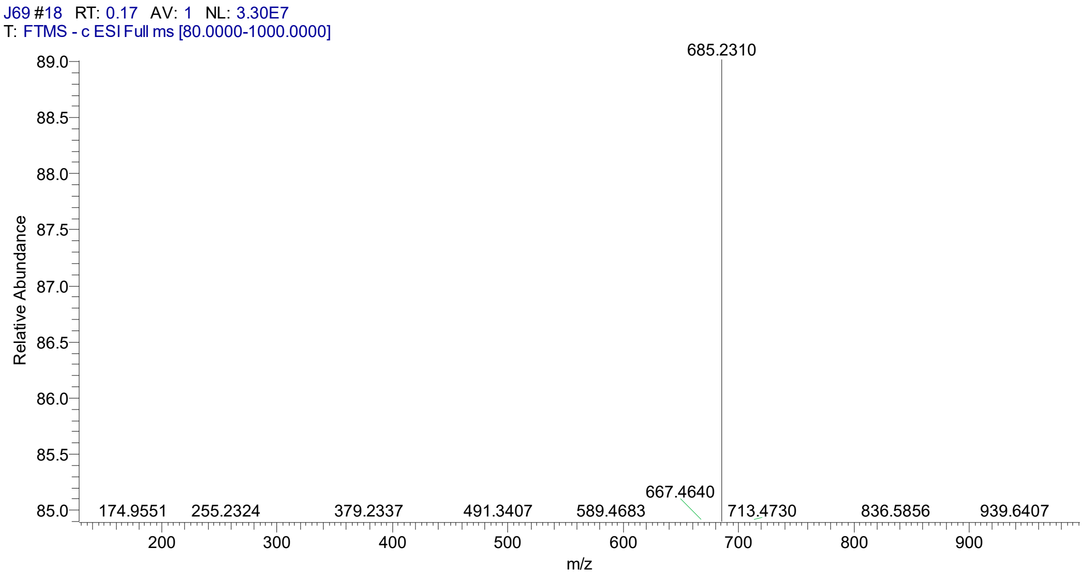

Figure S48.HR Mass spectrum (ESI) of compound **27b**.

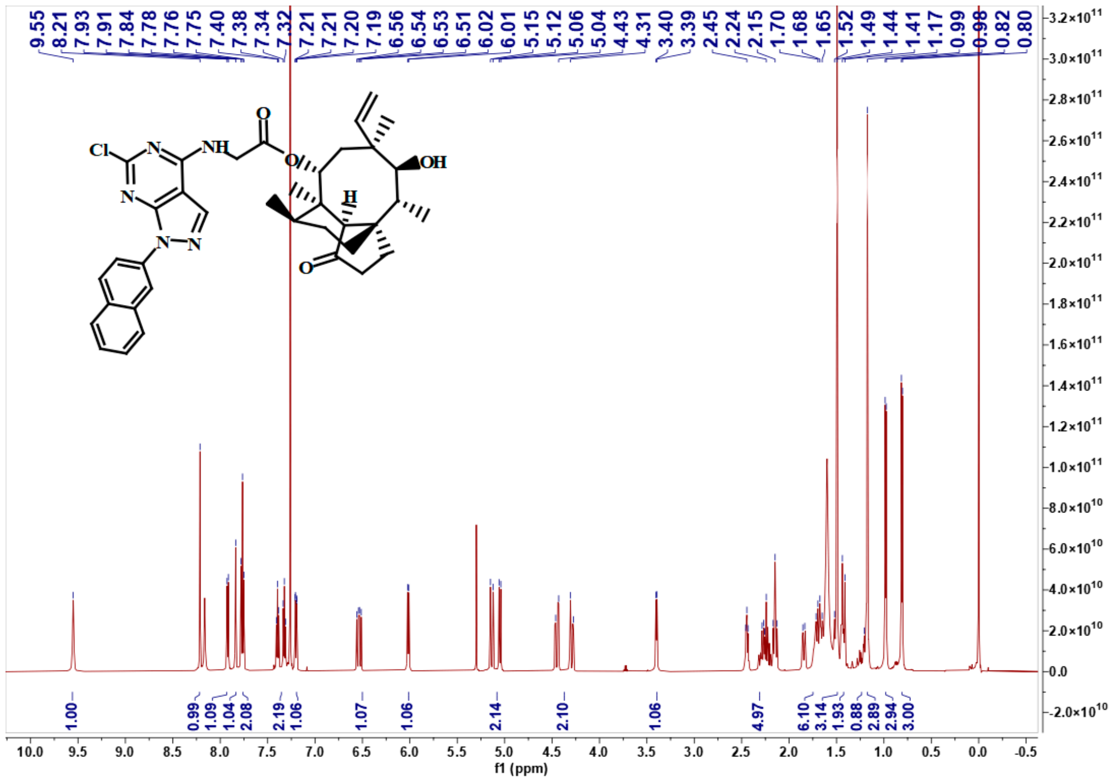

Figure S49.^{1}H-NMR spectrum ($CDCl_3$, 600MHz) of compound **28b.**

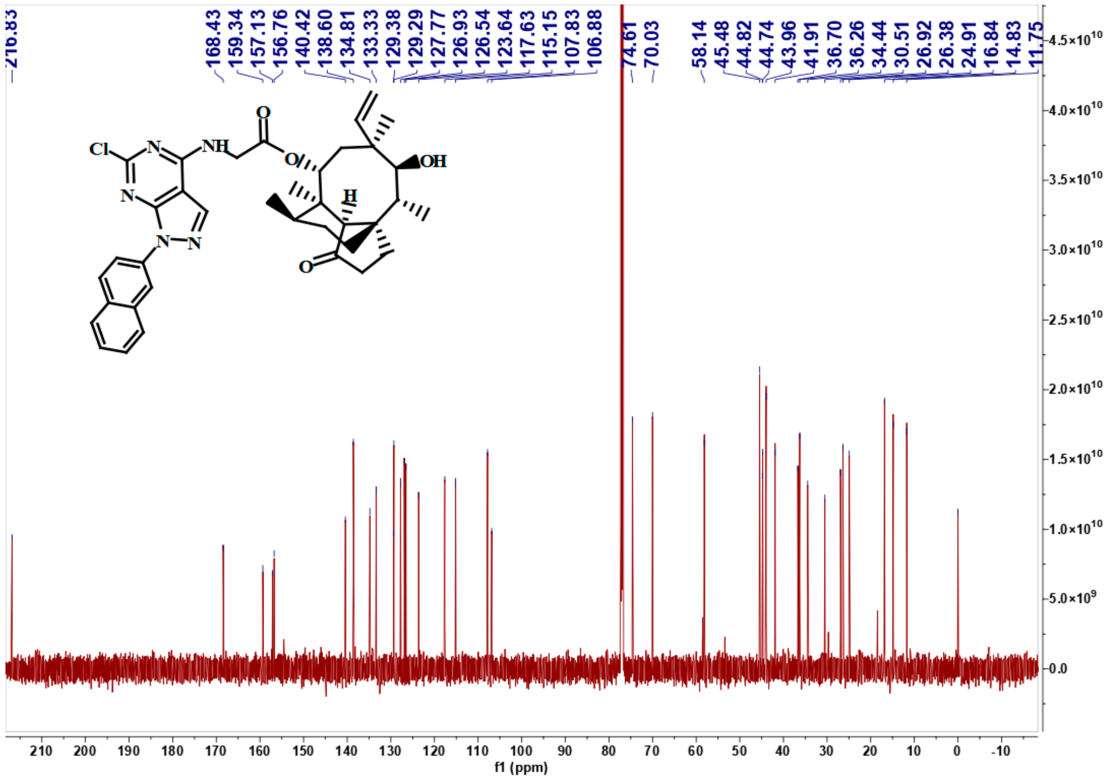

Figure S50.^{13}C-NMR spectrum ($CDCl_3$, 151MHz) of compound **28b**.

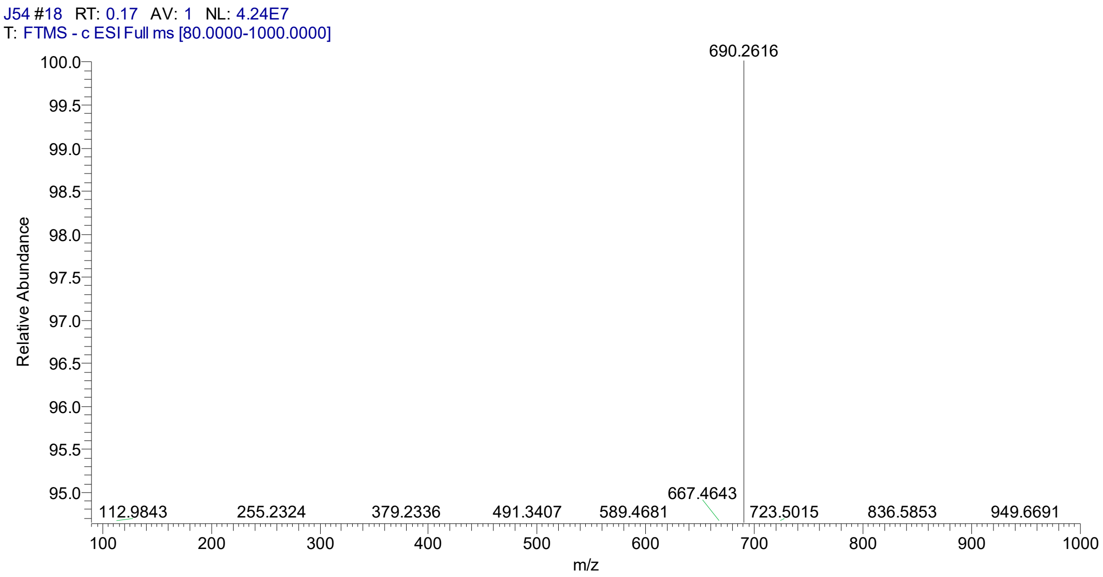

Figure S51.HR Mass spectrum (ESI) of compound **28b**.

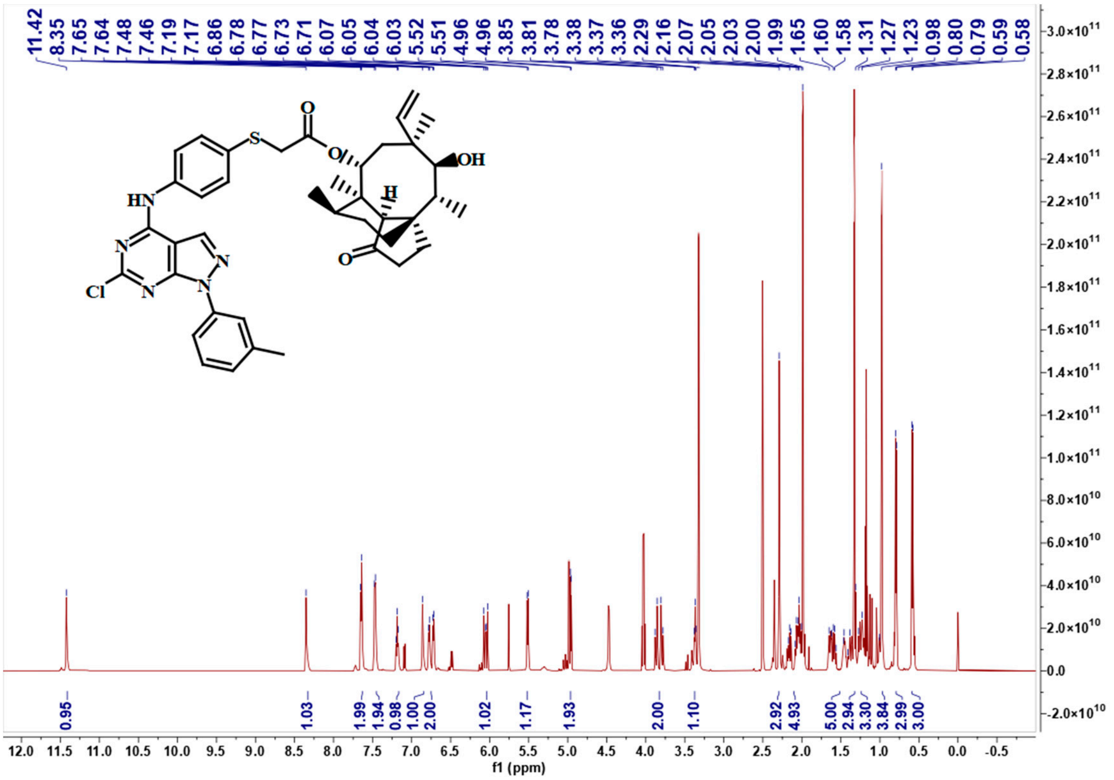

Figure S52.^{1}H-NMR spectrum (DMSO3, 600MHz) of compound **12c**.

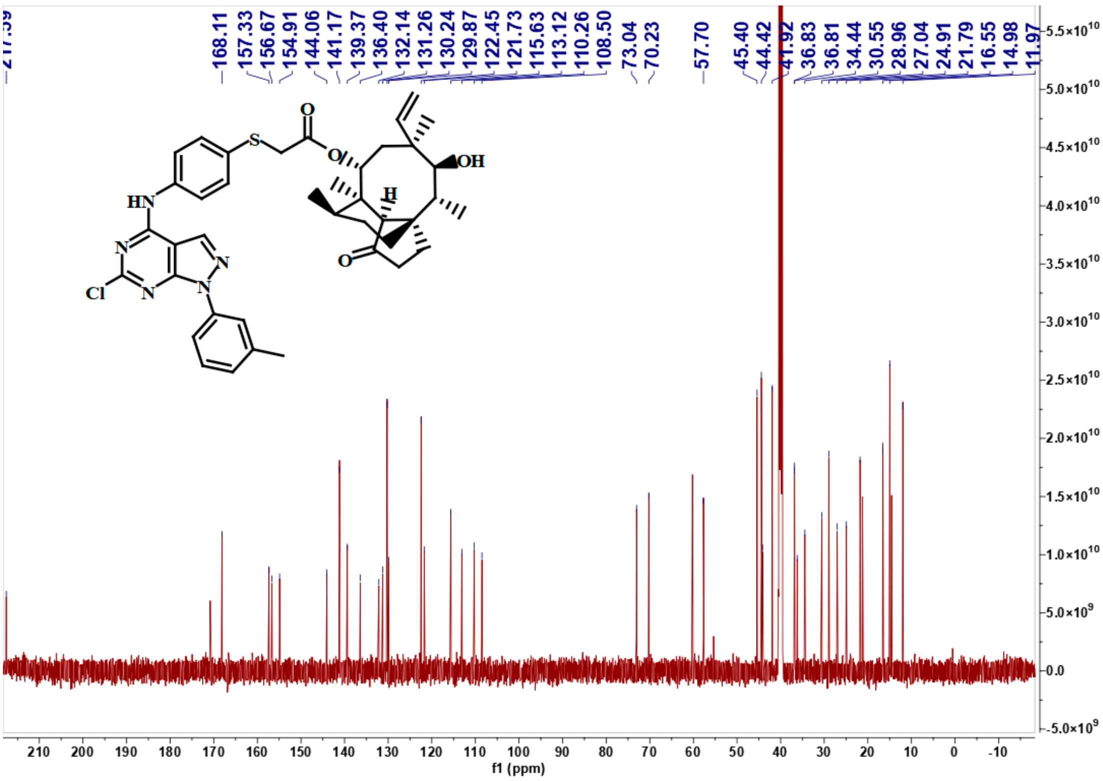

Figure S53.^{13}C-NMR spectrum (DMSO, 151MHz) of compound **12c**.

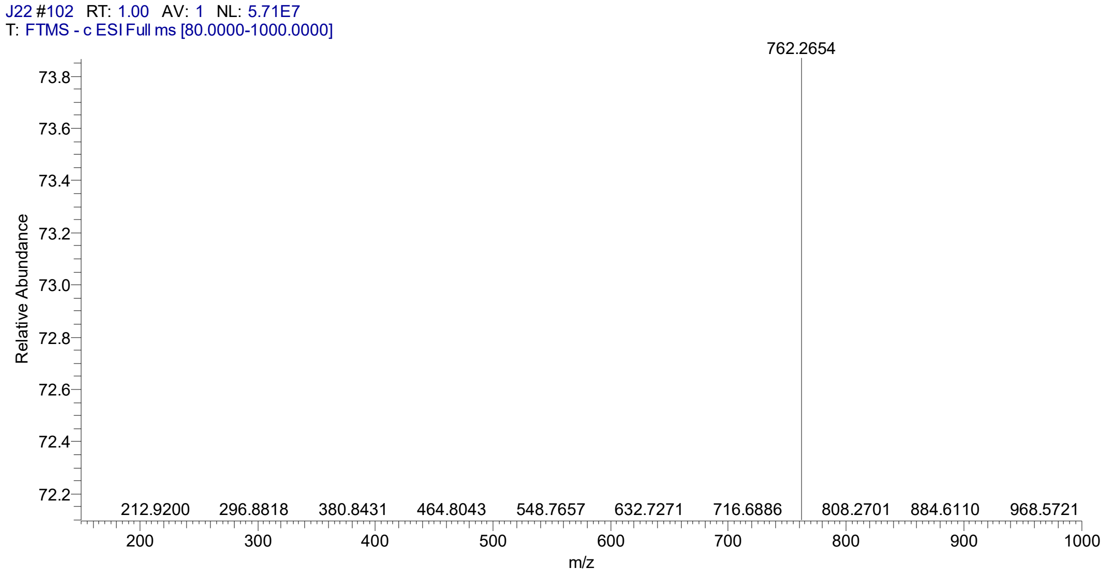

Figure S54.HR Mass spectrum (ESI) of compound **12c**.

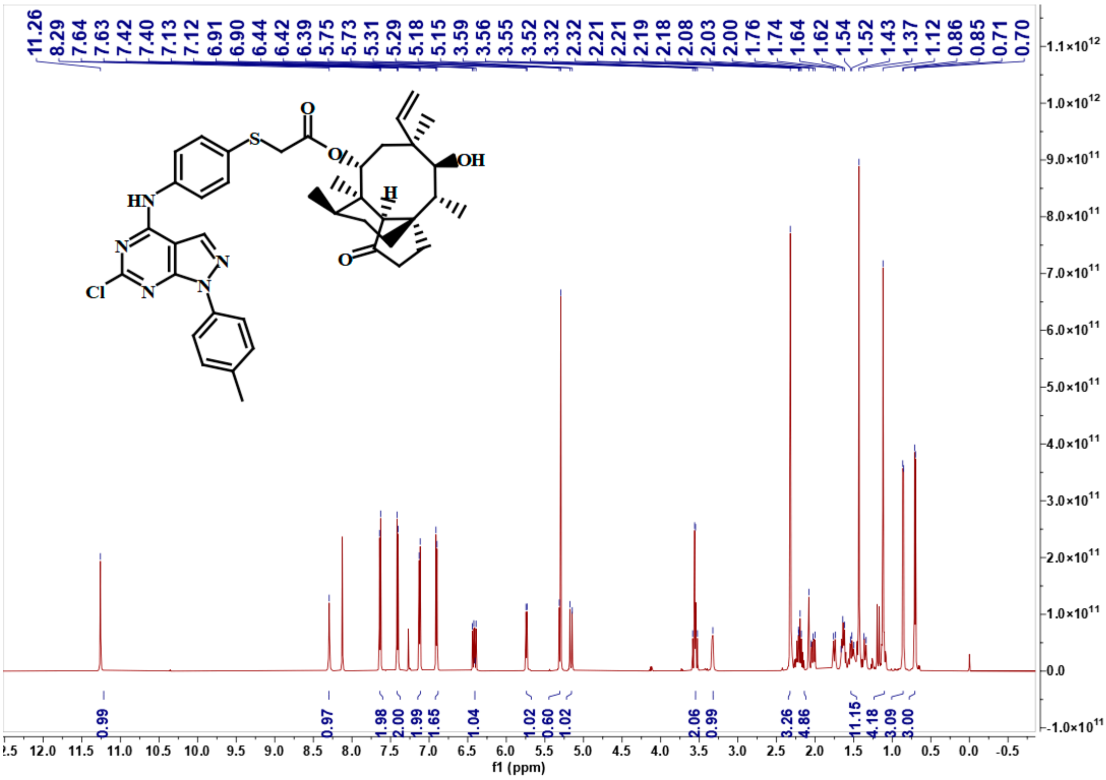

Figure S55.^{1}H-NMR spectrum ($CDCl_3$, 600MHz) of compound **13c**.

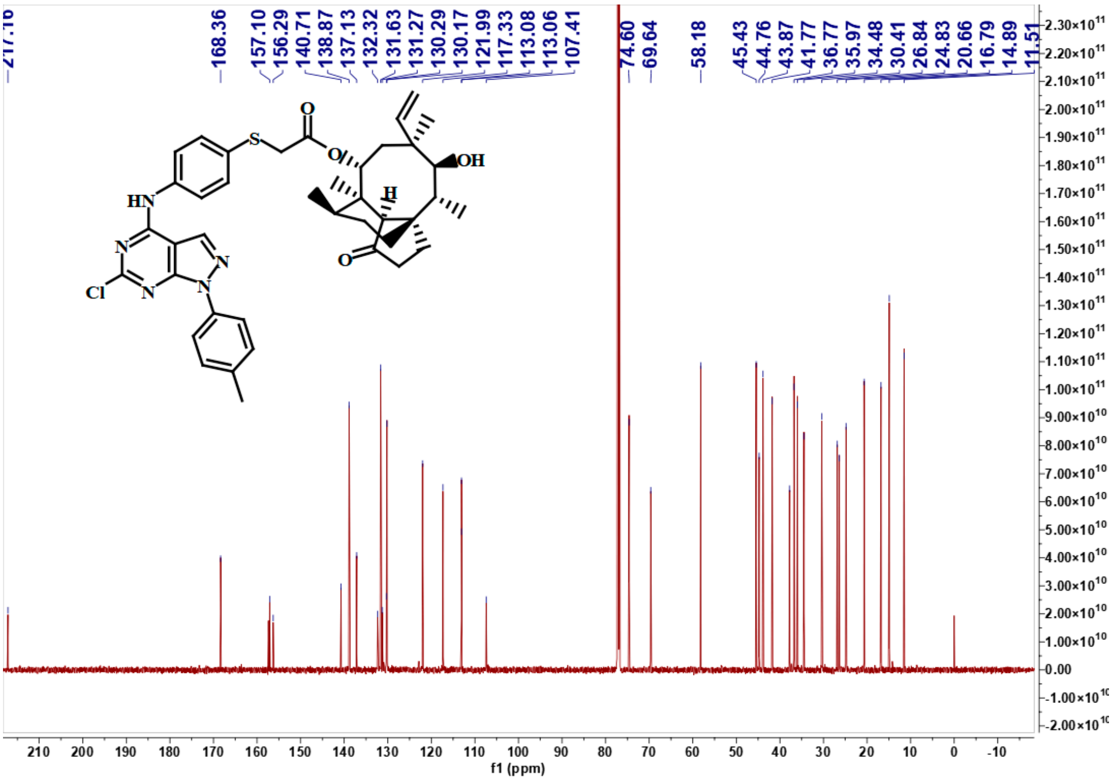

Figure S56.^{13}C-NMR spectrum ($CDCl_3$, 151MHz) of compound **13c**.

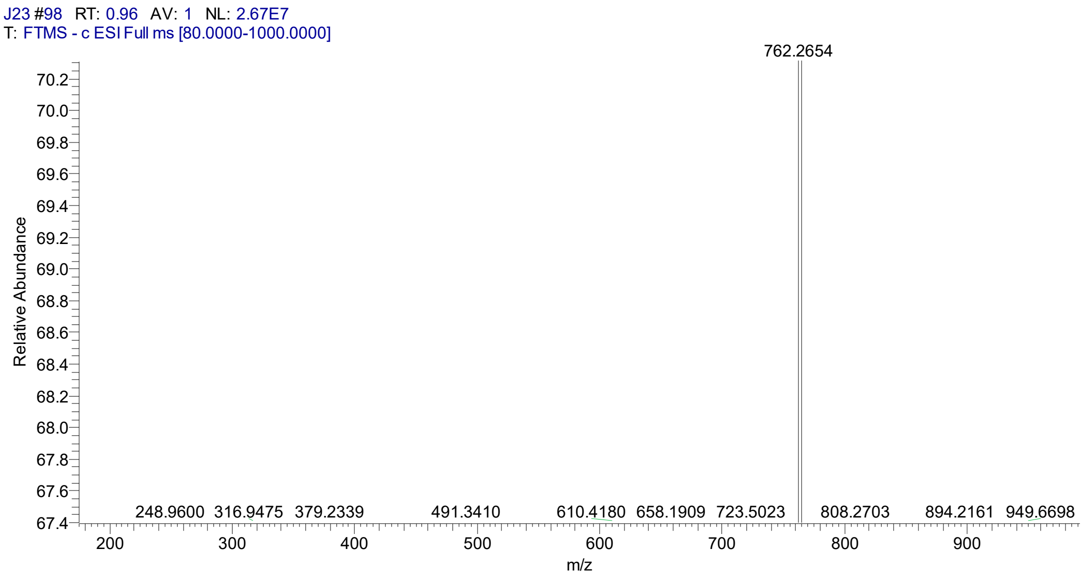

Figure S57.HR Mass spectrum (ESI) of compound **13c**.

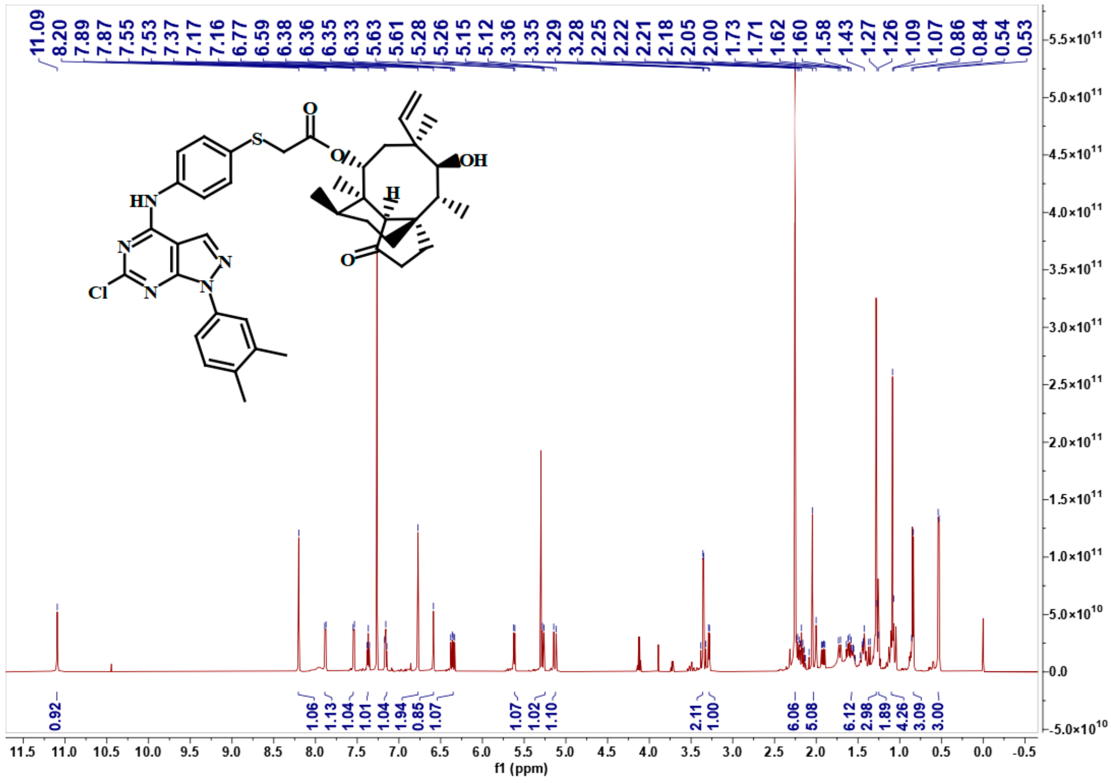

Figure S58.^{1}H-NMR spectrum ($CDCl_3$, 600MHz) of compound **14c**.

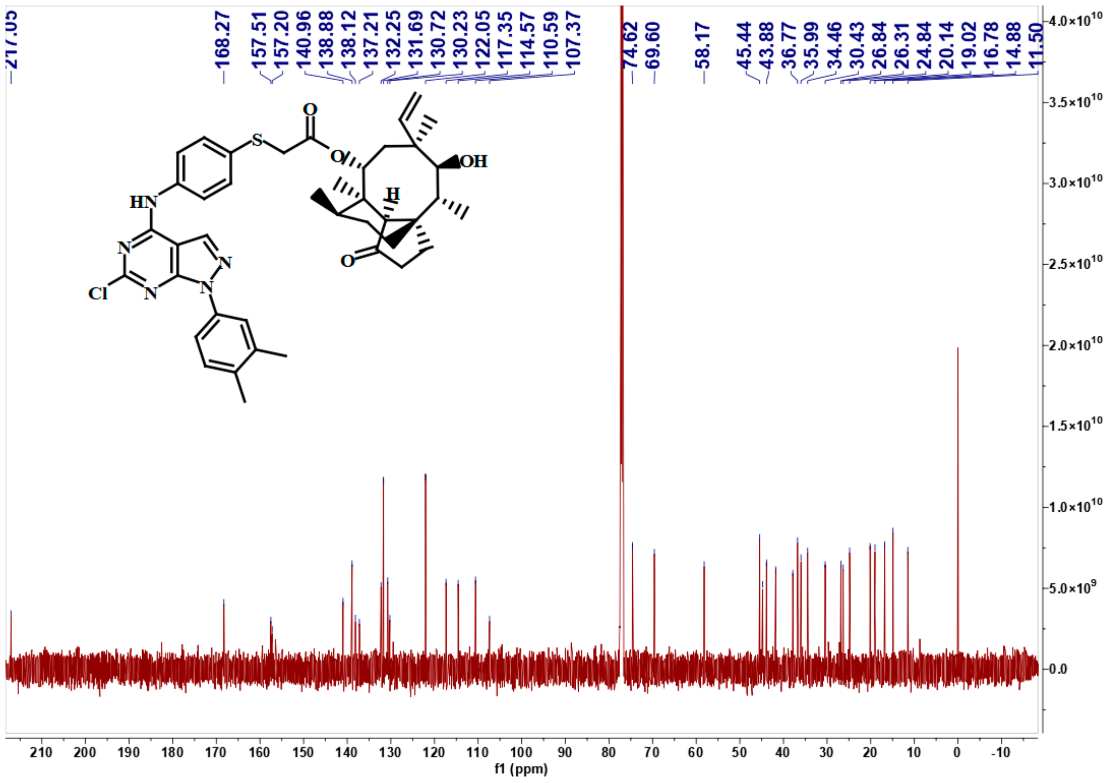

Figure S59.^{13}C-NMR spectrum ($CDCl_3$, 151MHz) of compound **14c**.

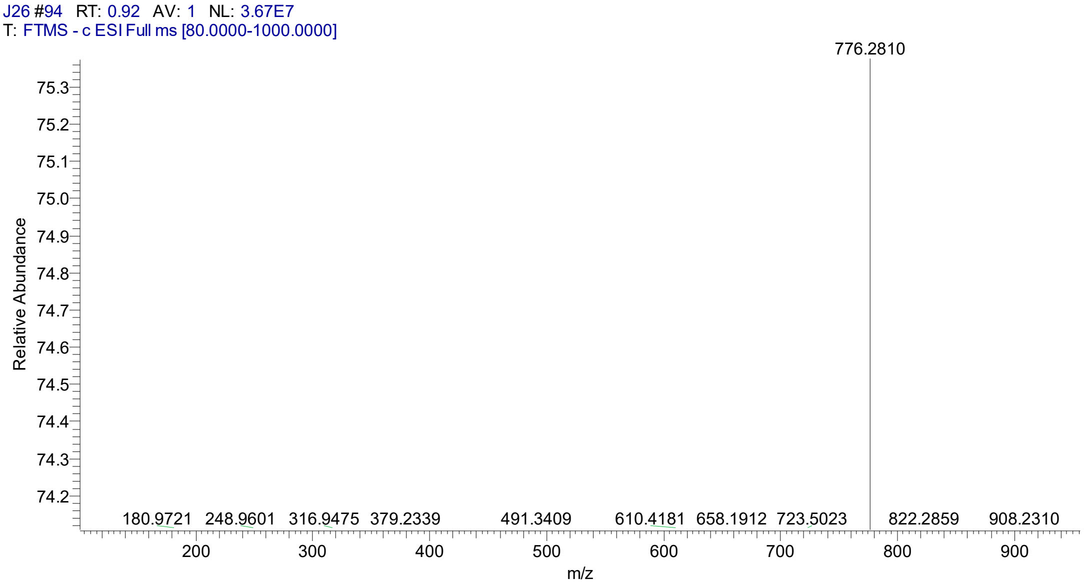

Figure S60.HR Mass spectrum (ESI) of compound **14c**.

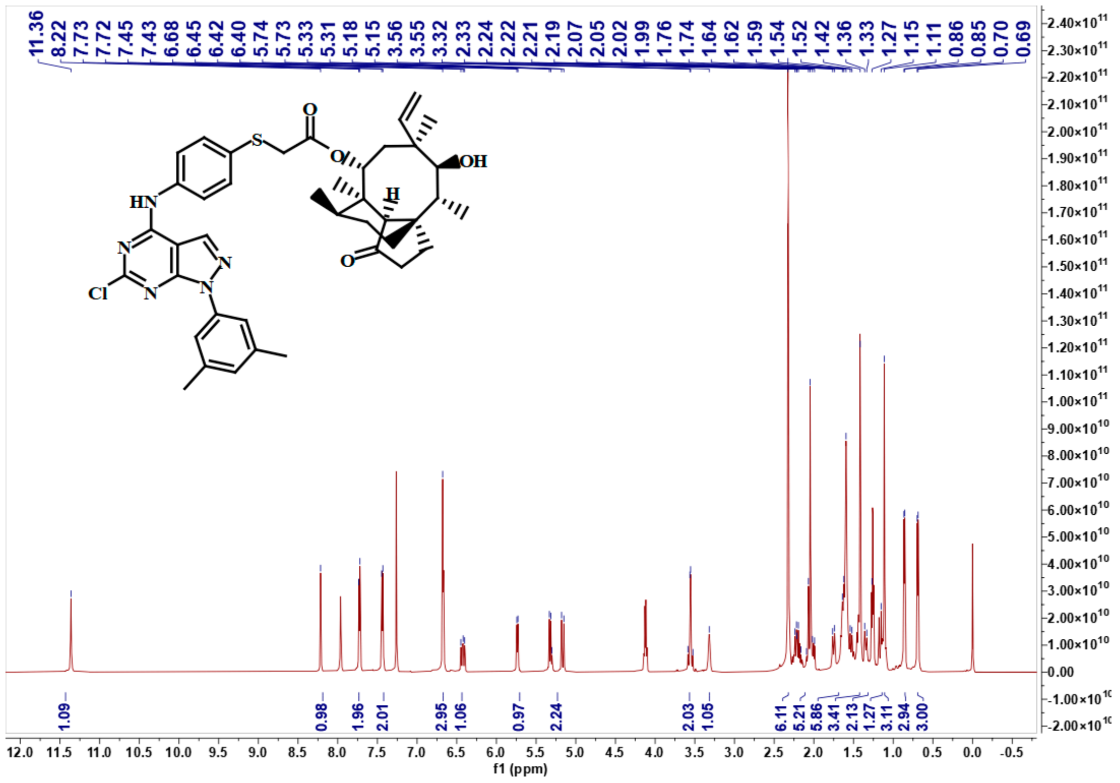

Figure S61.^{1}H-NMR spectrum ($CDCl_3$, 600MHz) of compound **15c**.

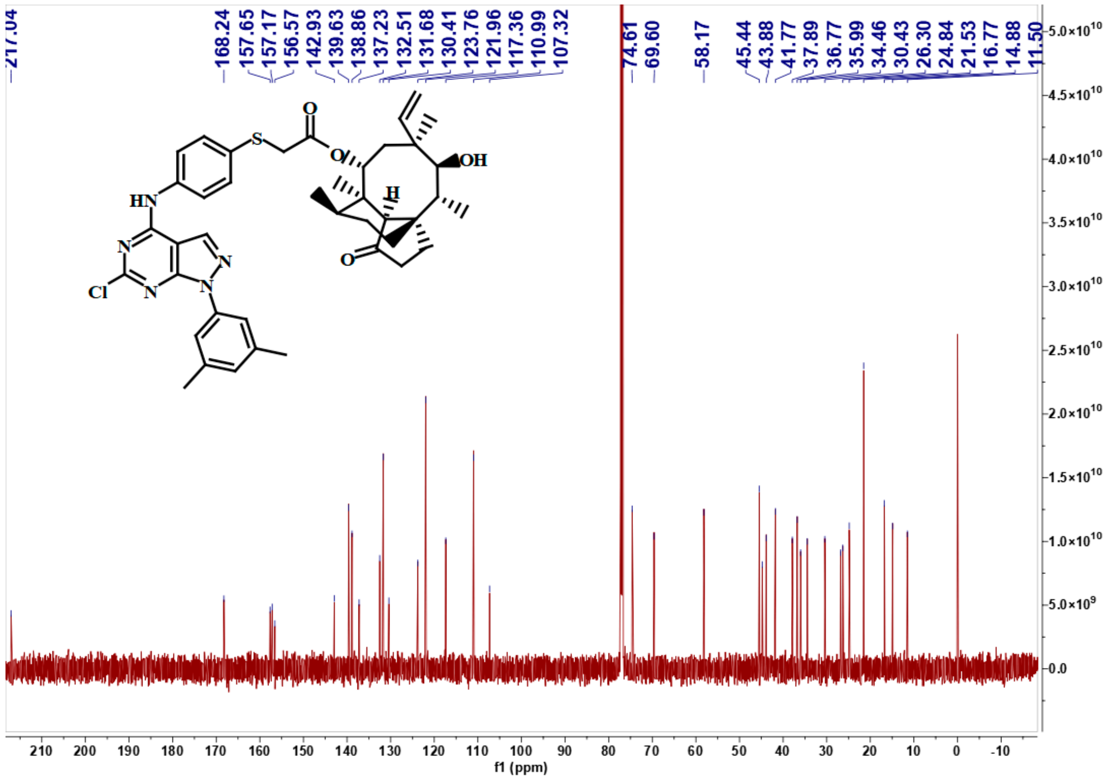

Figure S62.^{13}C-NMR spectrum ($CDCl_3$, 151MHz) of compound **15c**.

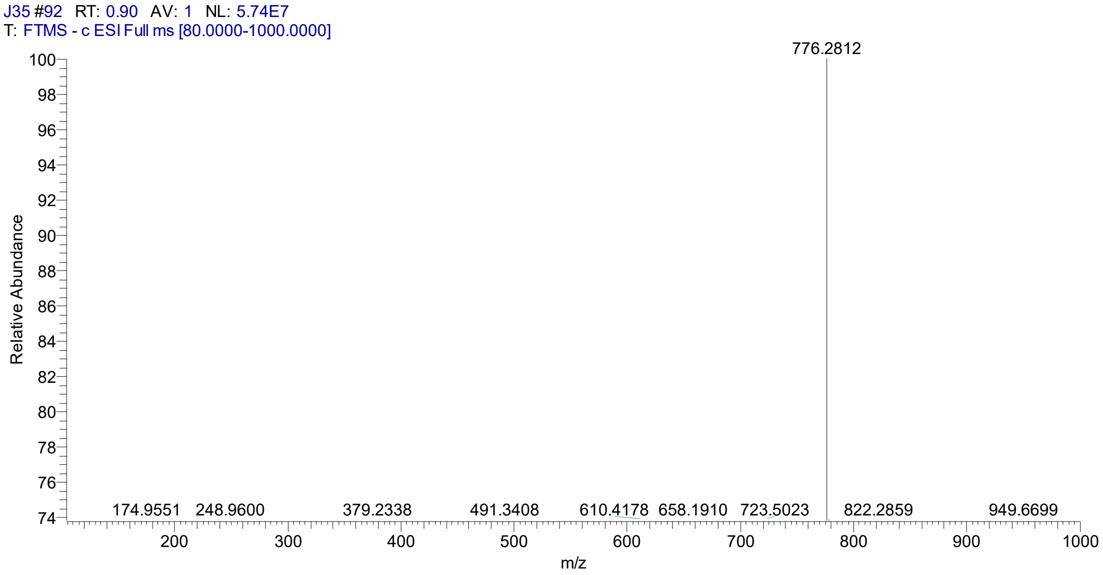

Figure S63.HR Mass spectrum (ESI) of compound **15c**.

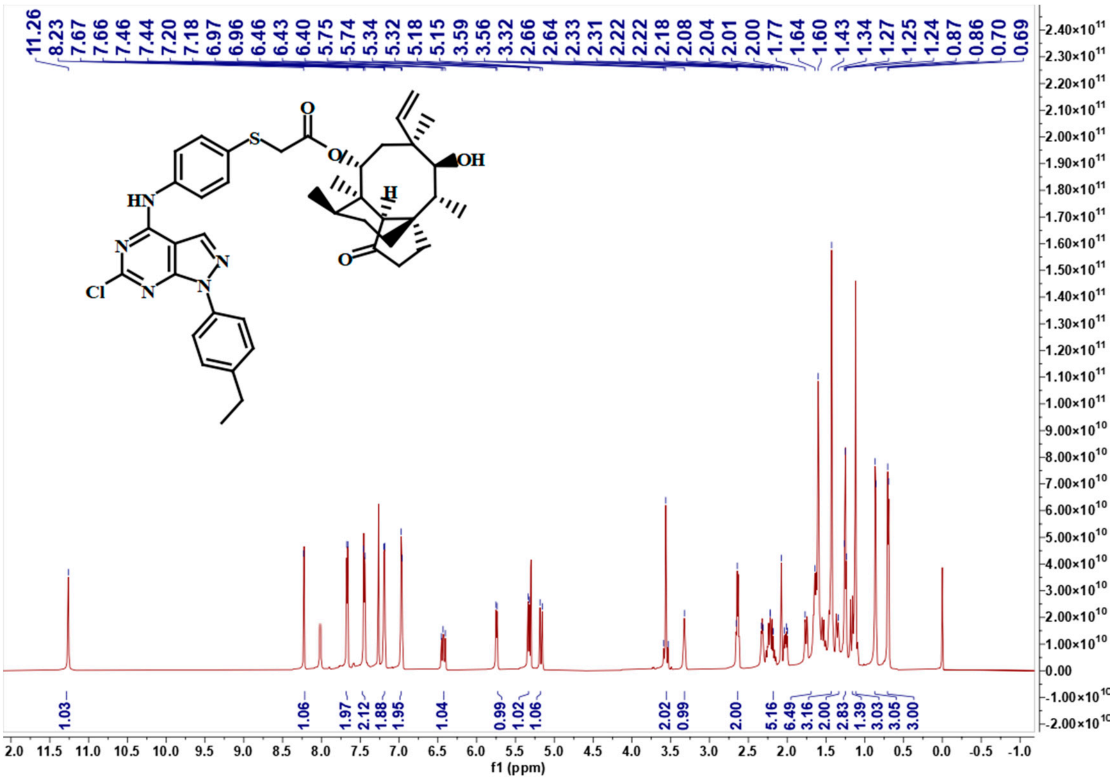

Figure S64.^{1}H-NMR spectrum ($CDCl_3$, 600MHz) of compound **16c**.

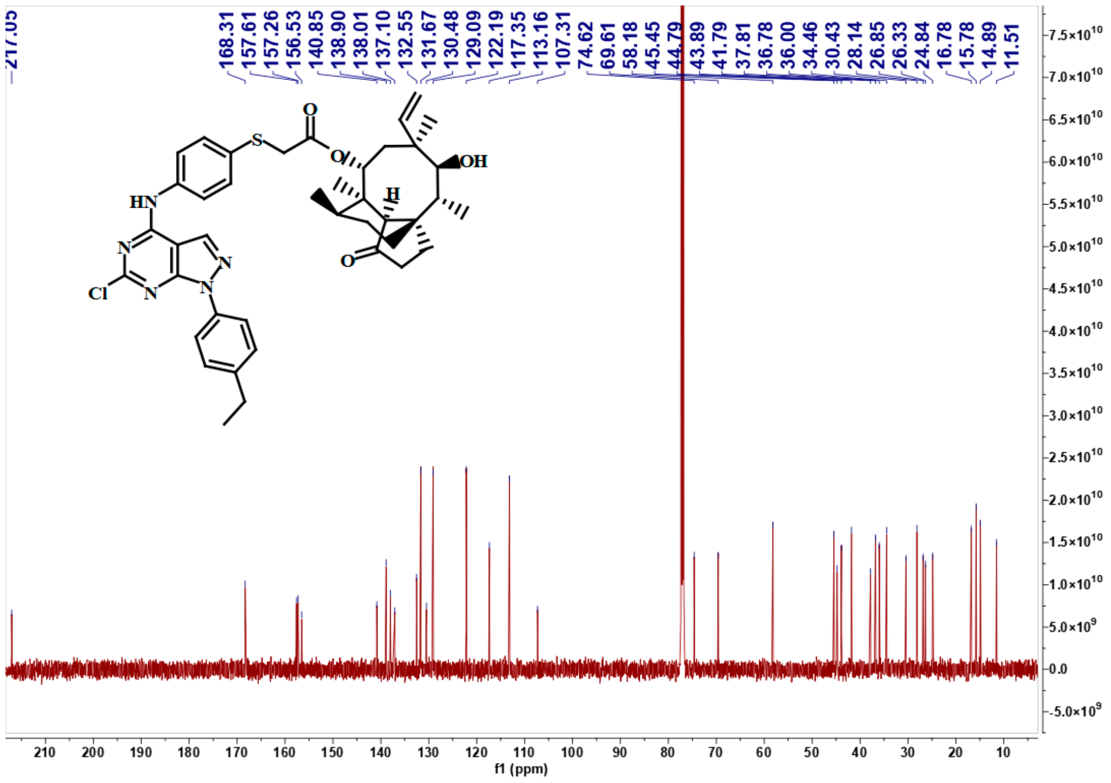

Figure S65.^{13}C-NMR spectrum ($CDCl_3$, 151MHz) of compound **16c**.

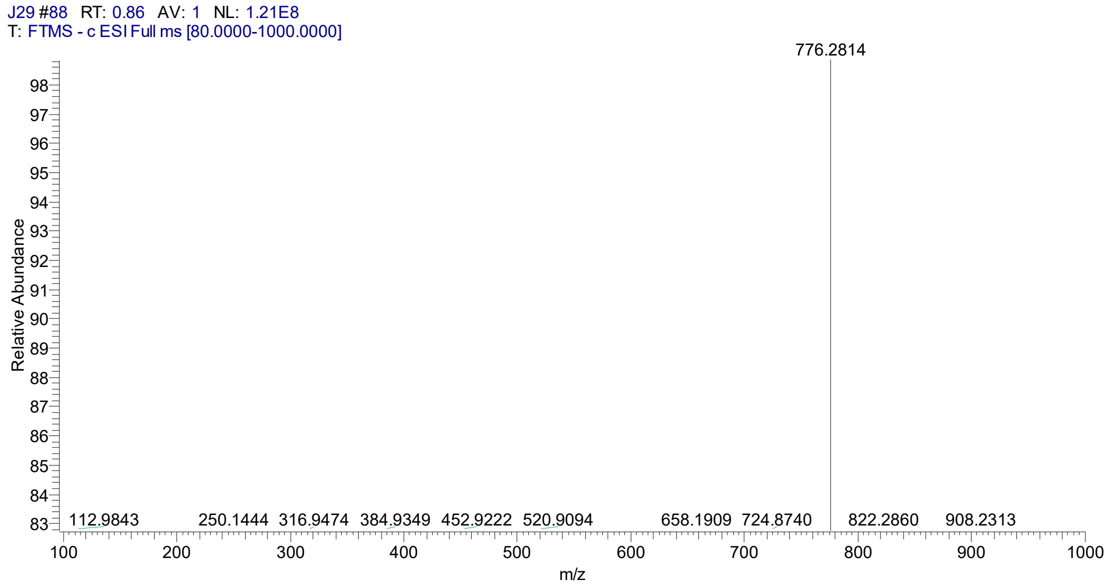

Figure S66.HR Mass spectrum (ESI) of compound **16c**.

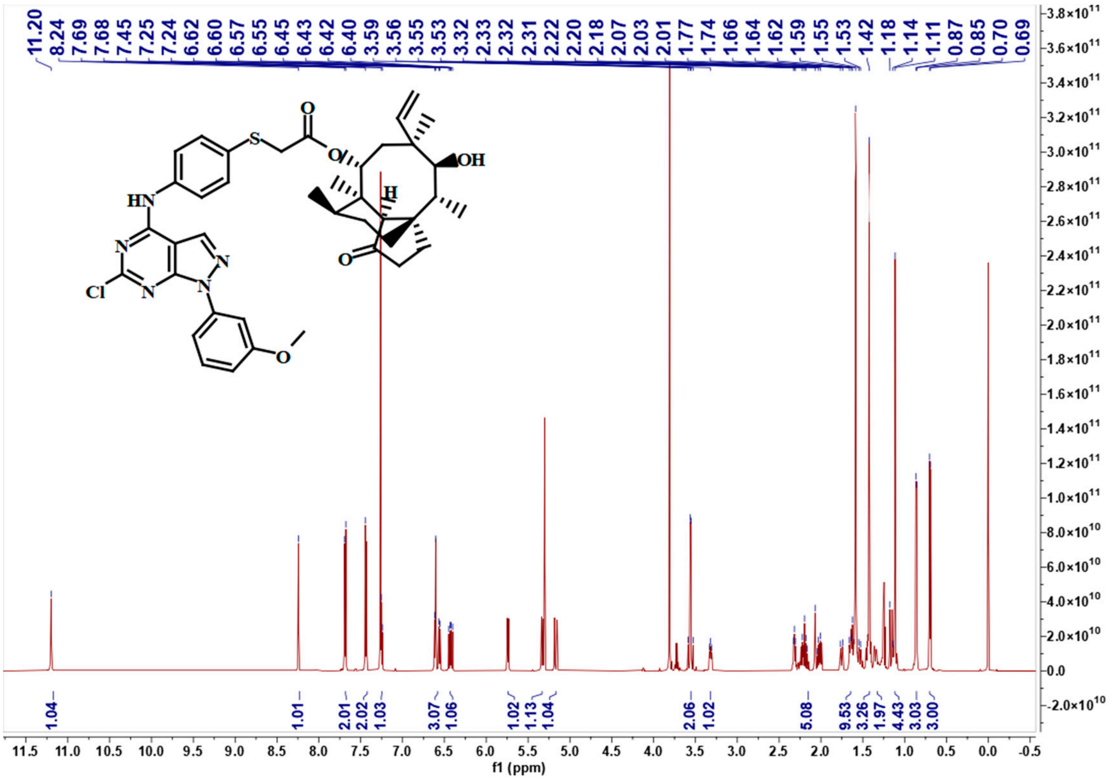

Figure S67.^{1}H-NMR spectrum ($CDCl_3$, 600MHz) of compound **17c**.

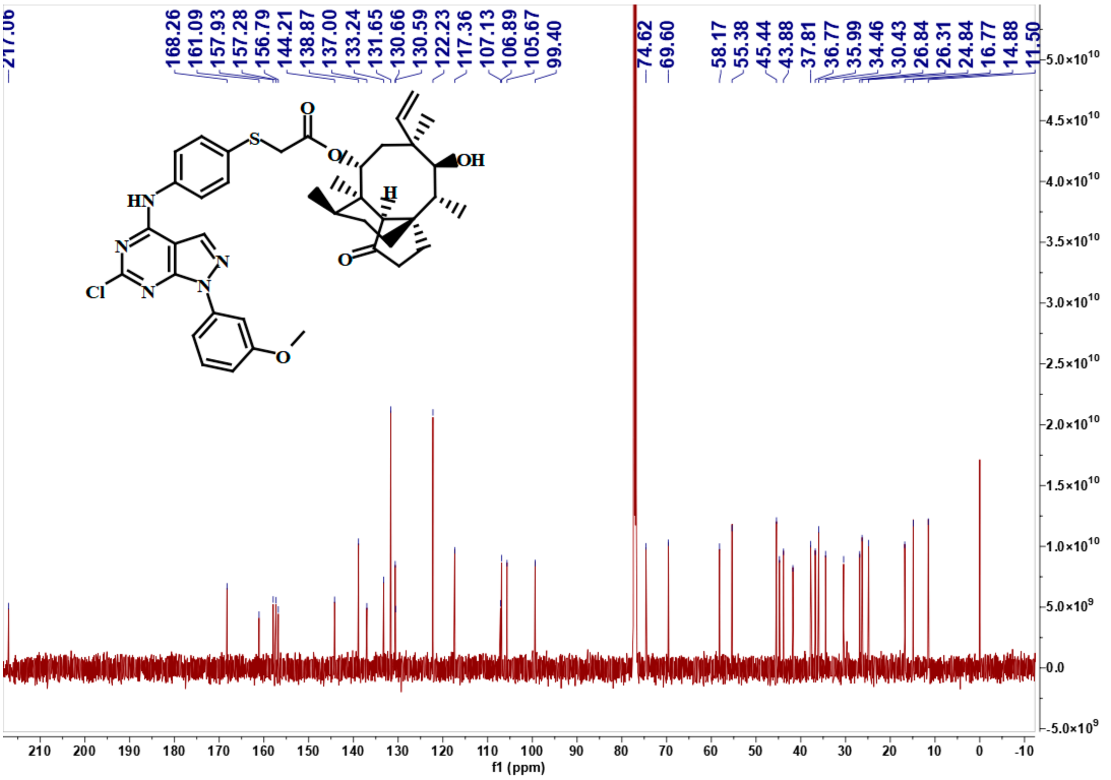

Figure S68.^{13}C-NMR spectrum ($CDCl_3$, 151MHz) of compound **17c**.

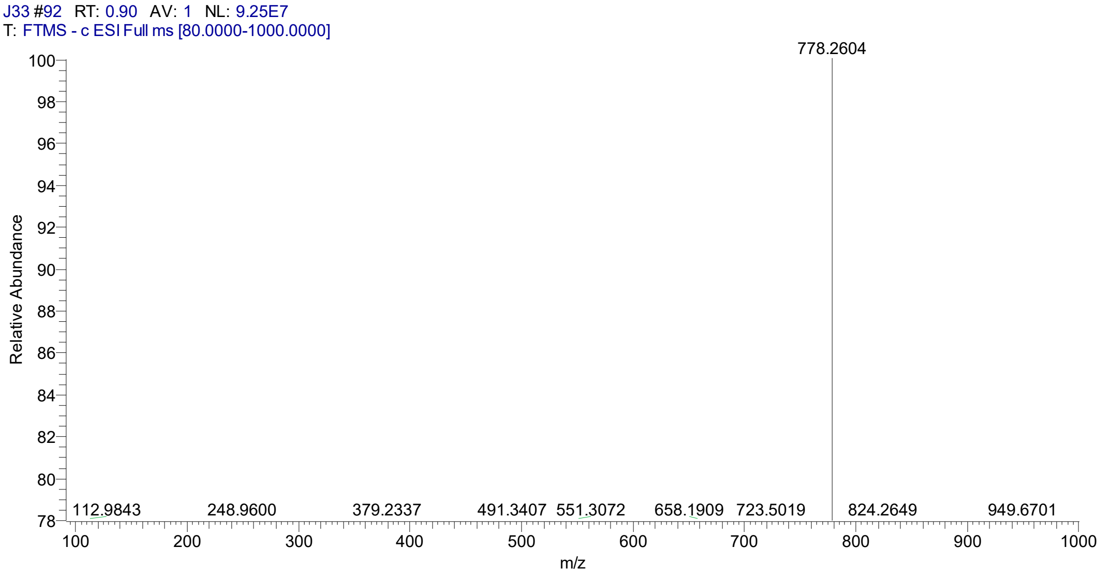

Figure S69.HR Mass spectrum (ESI) of compound **17c**.

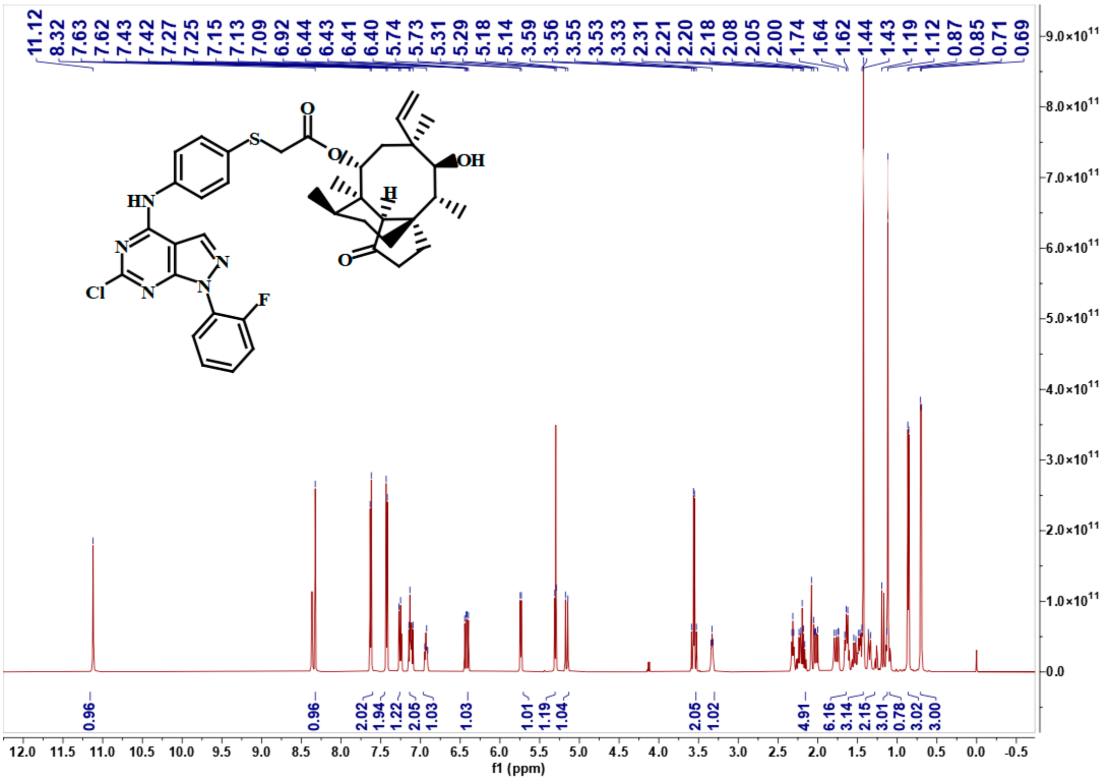

Figure S70.^{1}H-NMR spectrum ($CDCl_3$, 600MHz) of compound **18c**.

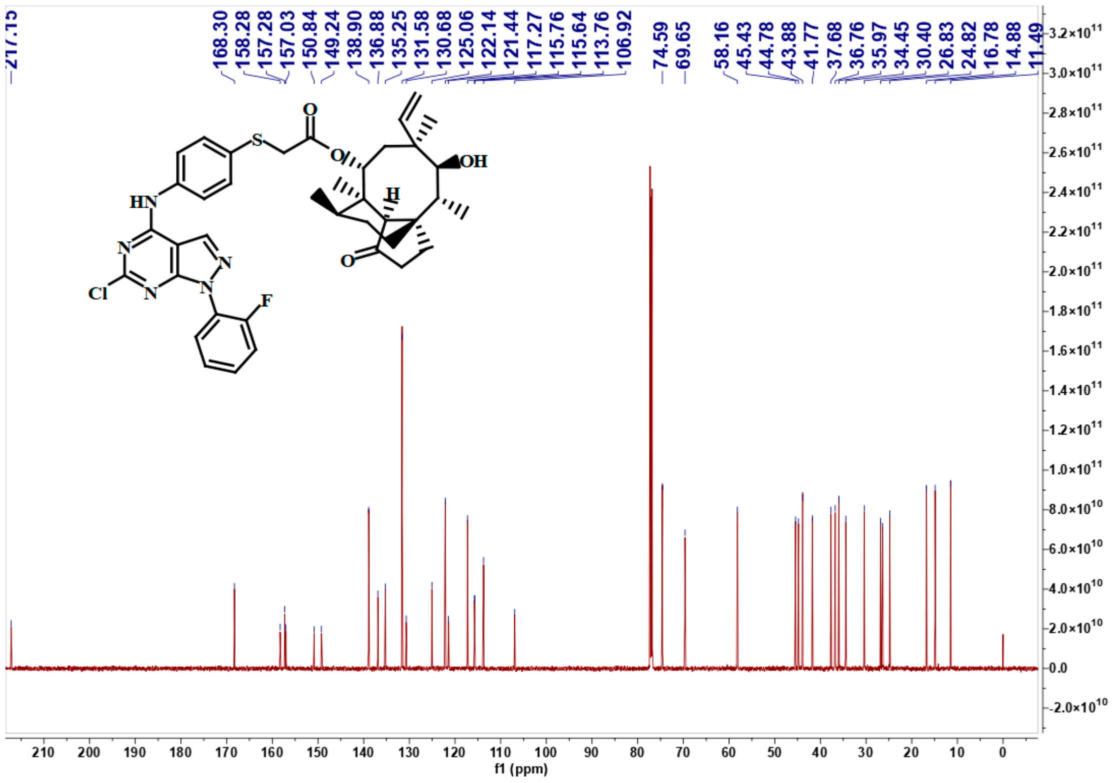

Figure S71.^{13}C-NMR spectrum ($CDCl_3$, 151MHz) of compound **18c**.

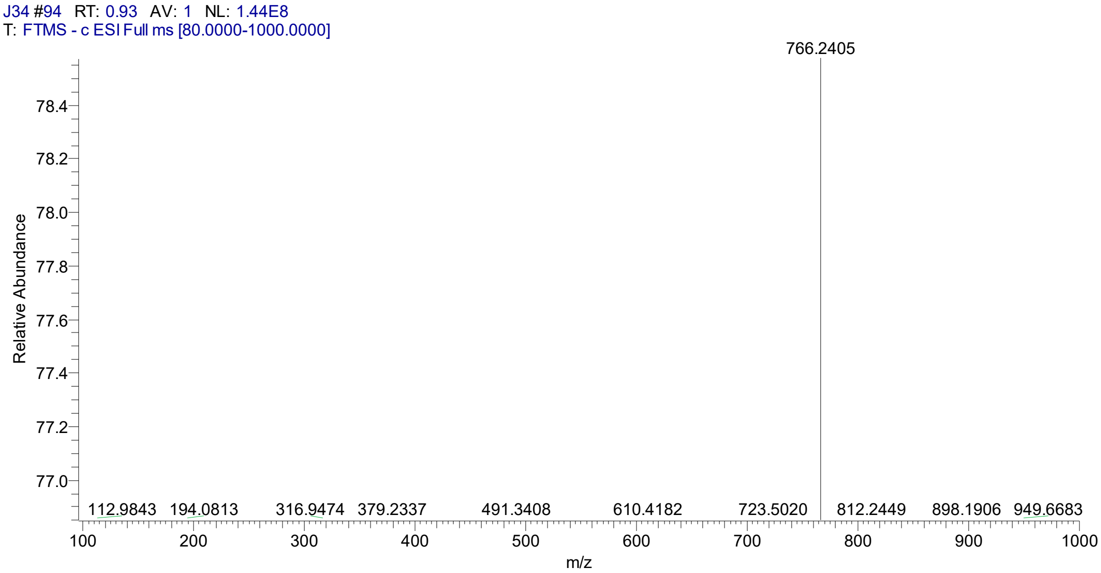

Figure S72.HR Mass spectrum (ESI) of compound **18c**.

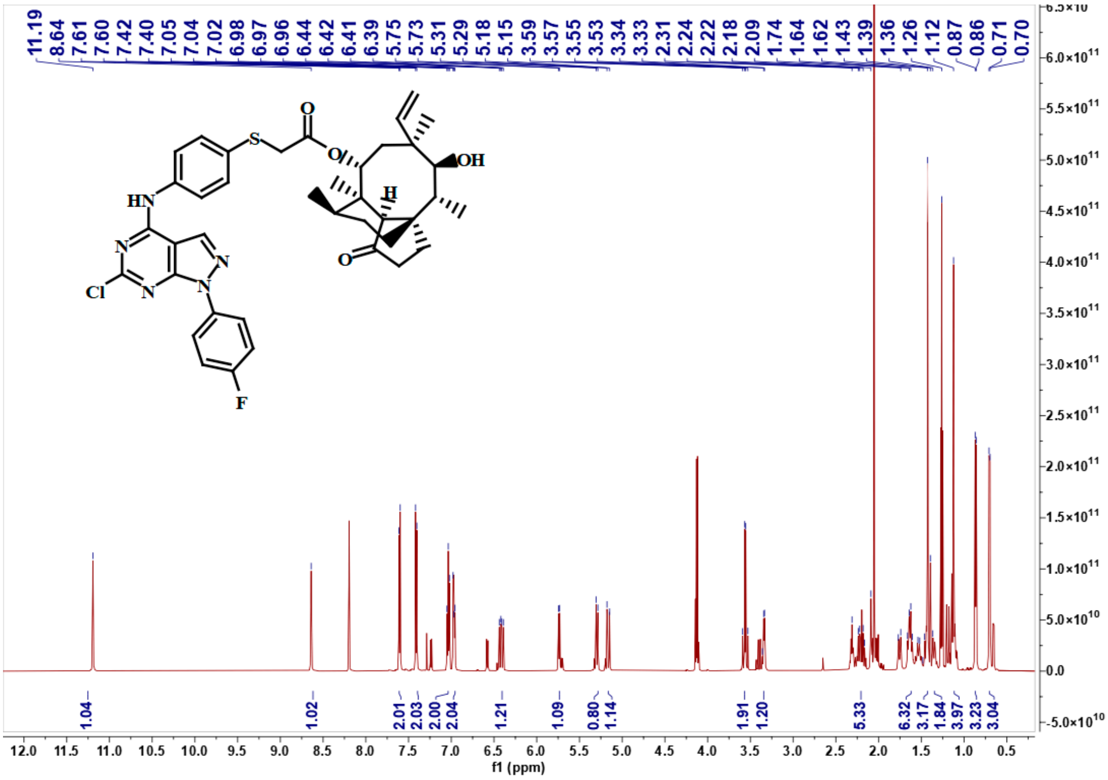

Figure S73.^{1}H-NMR spectrum ($CDCl_3$, 600MHz) of compound **19c**.

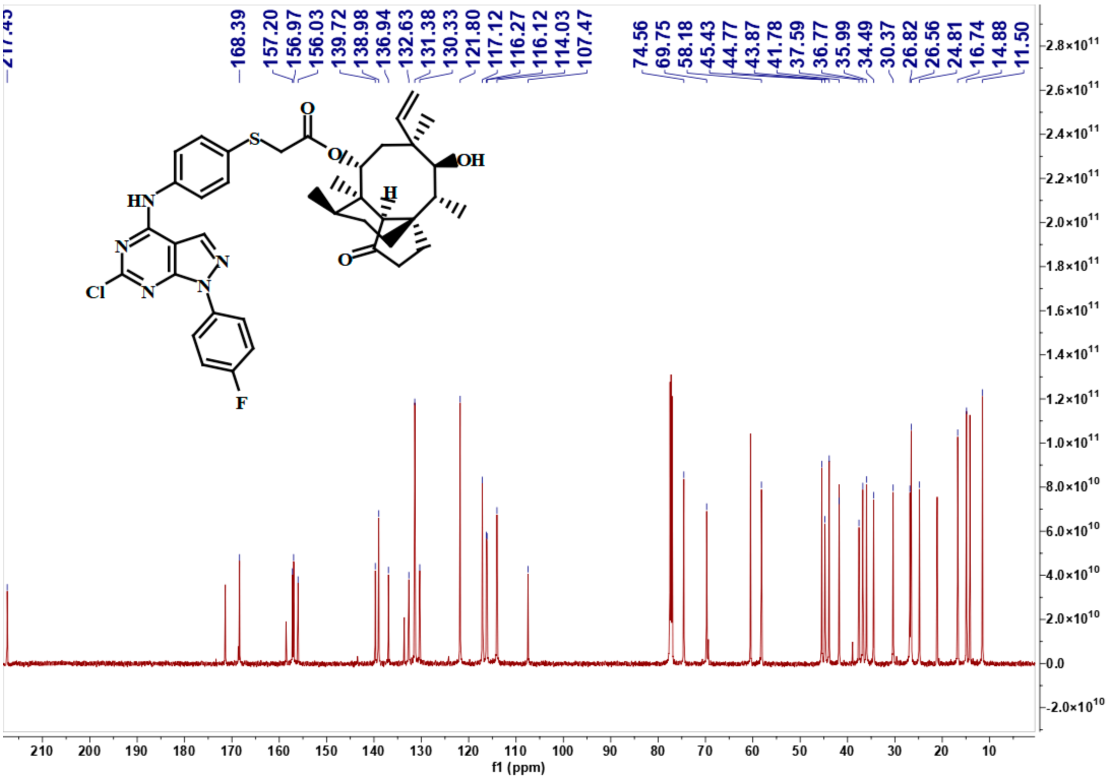

Figure S74.^{13}C-NMR spectrum ($CDCl_3$, 151MHz) of compound **19c**.

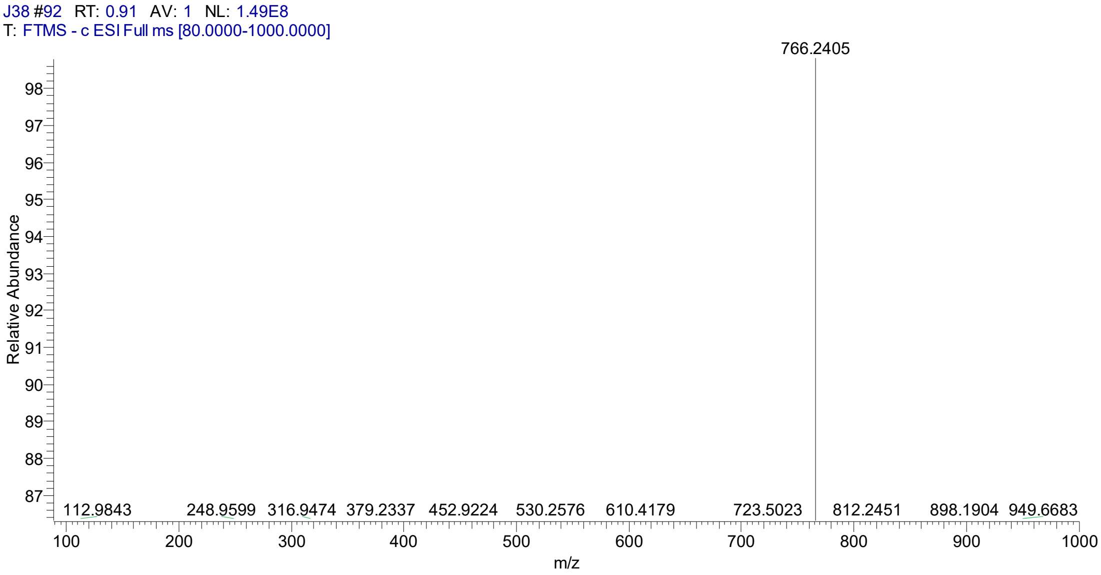

Figure S75.HR Mass spectrum (ESI) of compound **19c**.

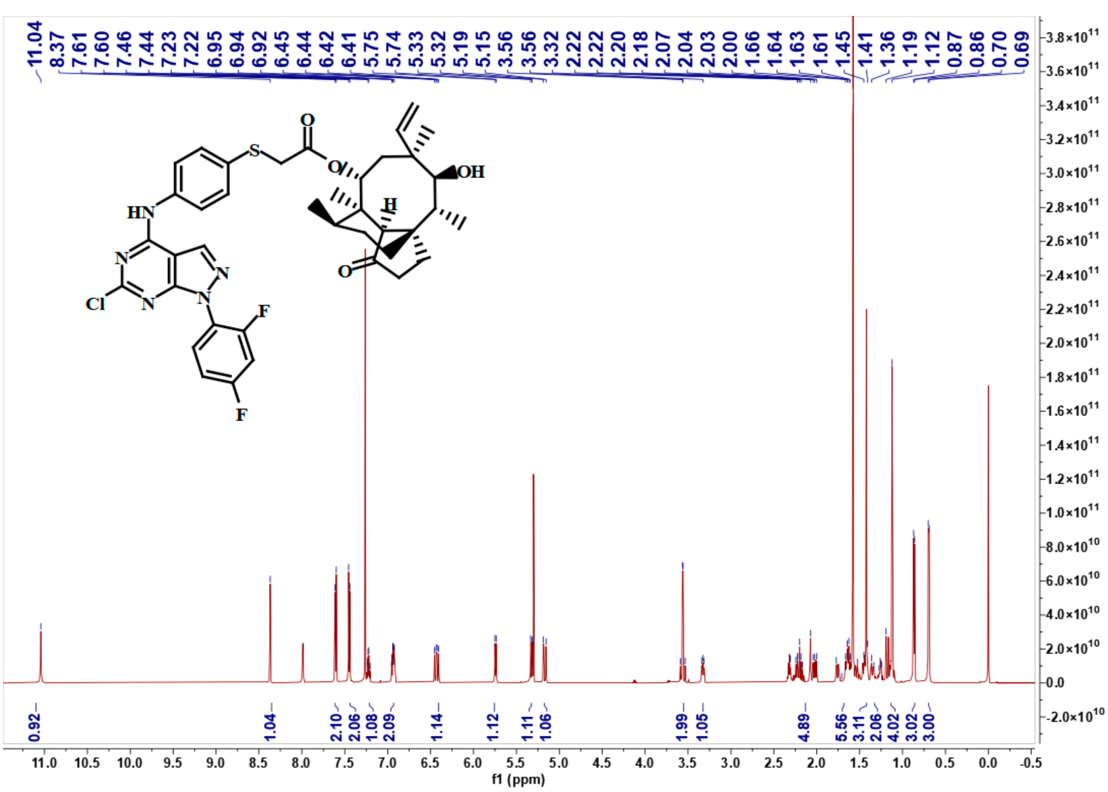

Figure S76.^{1}H-NMR spectrum ($CDCl_3$, 600MHz) of compound **20c**.

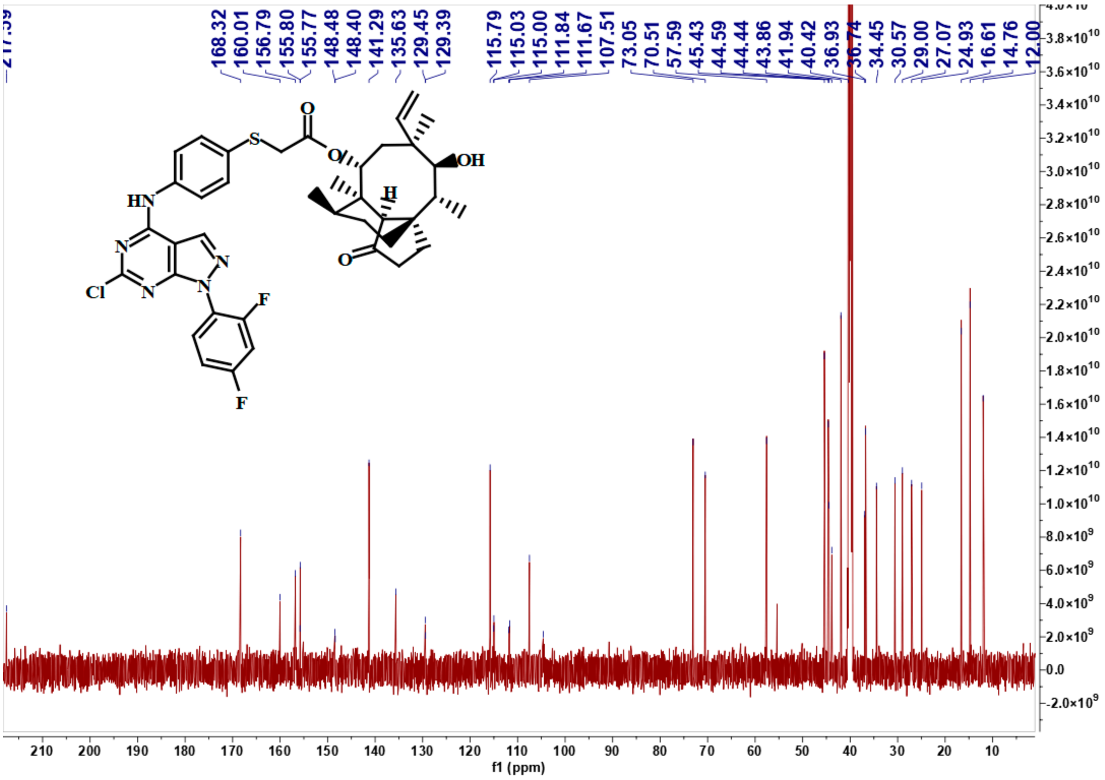

Figure S77.^{13}C-NMR spectrum (DMSO, 151MHz) of compound **20c**.

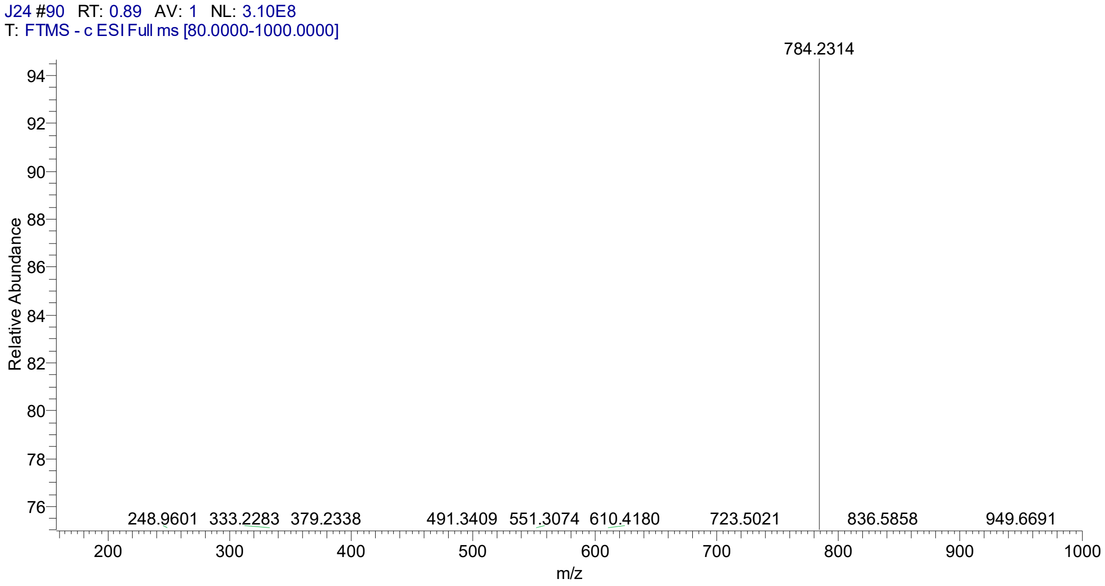

Figure S78.HR Mass spectrum (ESI) of compound **20c**.

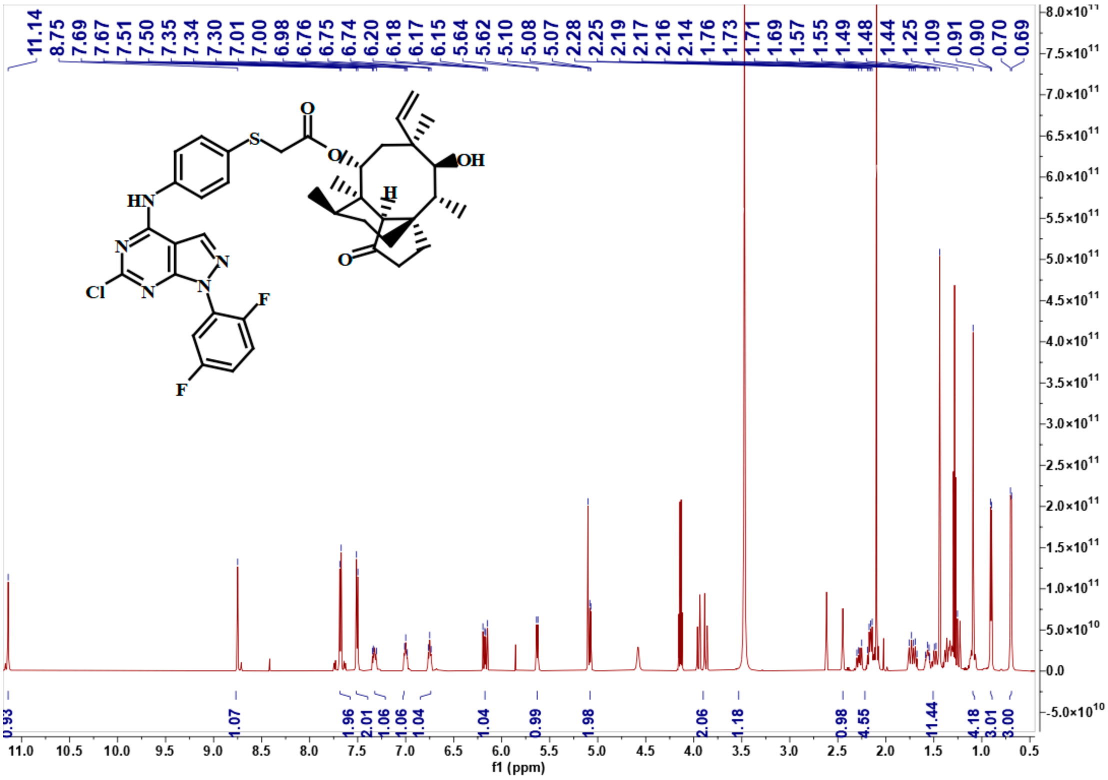

Figure S79.^{1}H-NMR spectrum (DMSO, 600MHz) of compound **21c**.

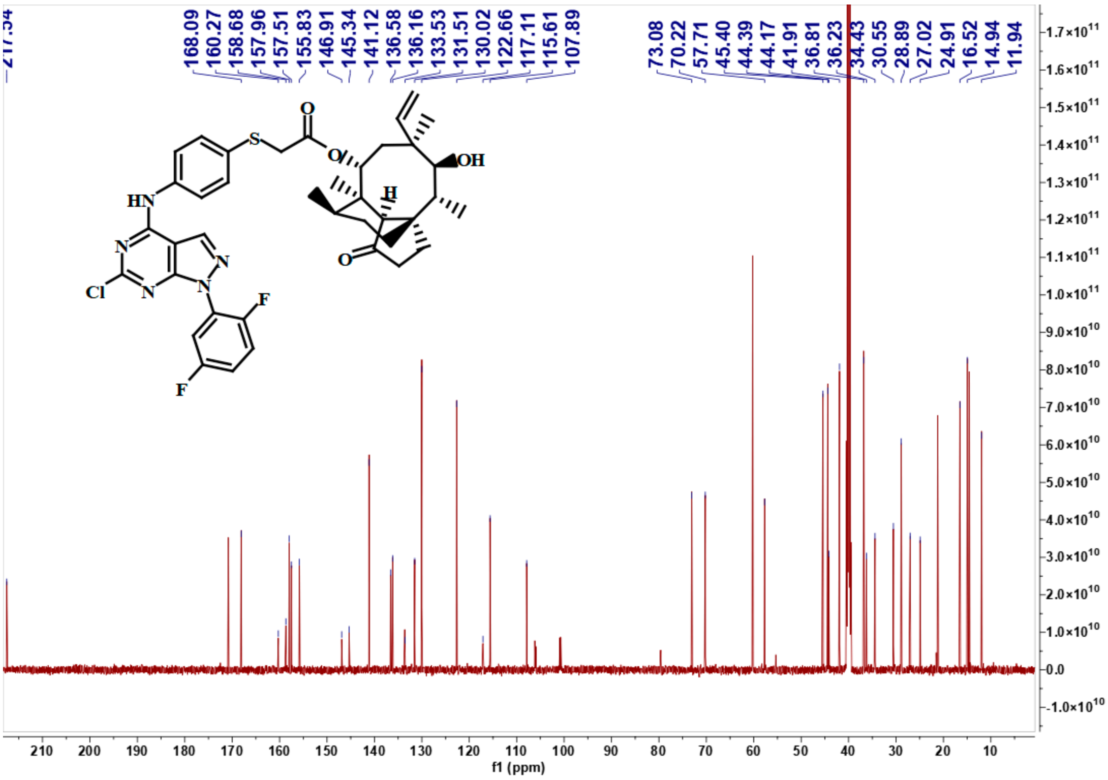

Figure S80.^{13}C-NMR spectrum (DMSO, 151MHz) of compound **21c**.

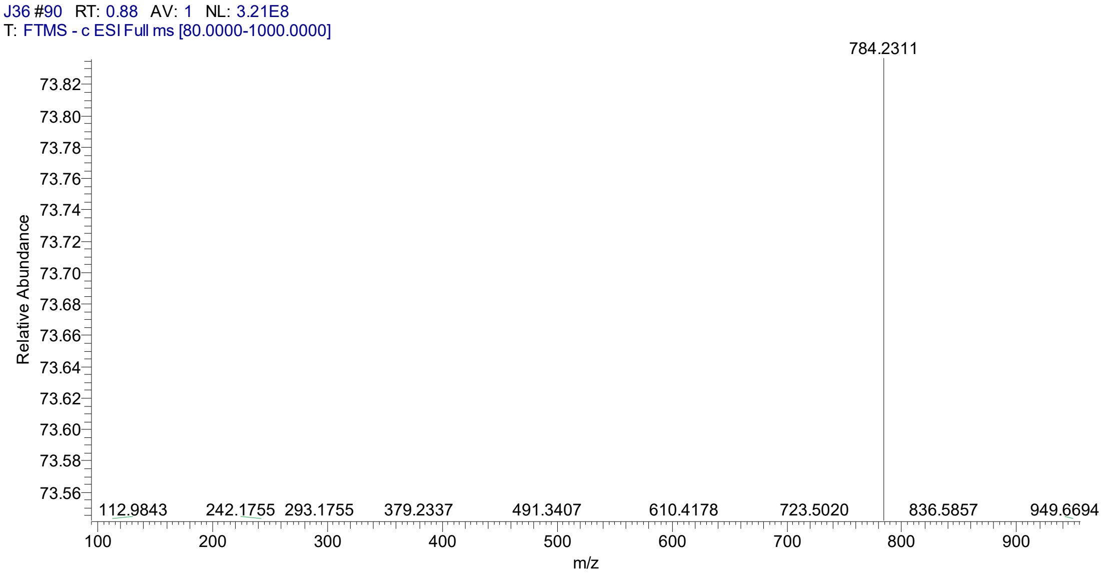

Figure S81.HR Mass spectrum (ESI) of compound **21c**.

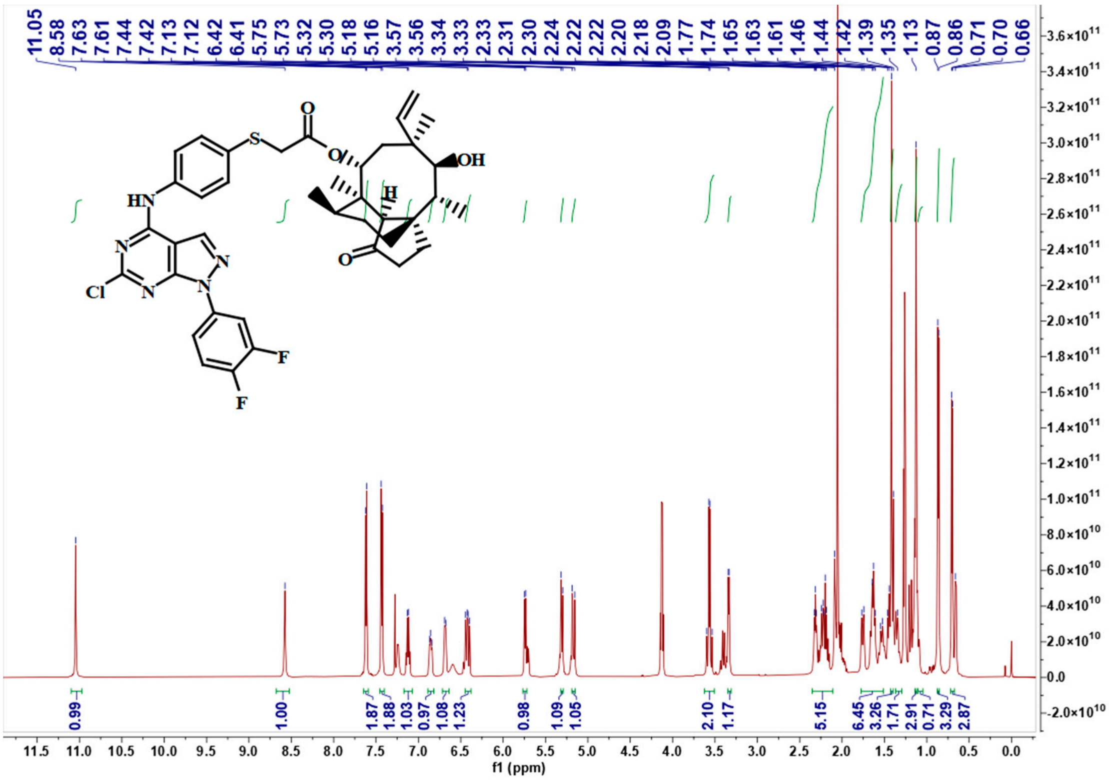

Figure S82.^{1}H-NMR spectrum ($CDCl_3$, 600MHz) of compound **22c**.

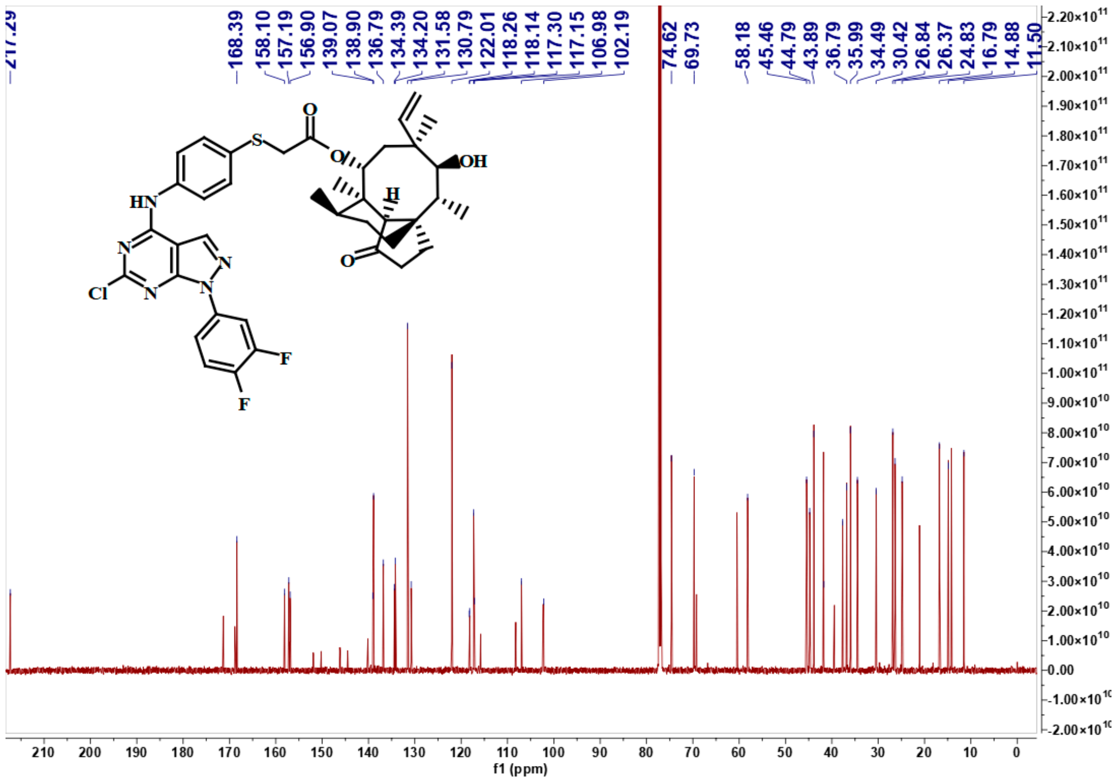

Figure S83.^{13}C-NMR spectrum ($CDCl_3$, 151MHz) of compound **22c**.

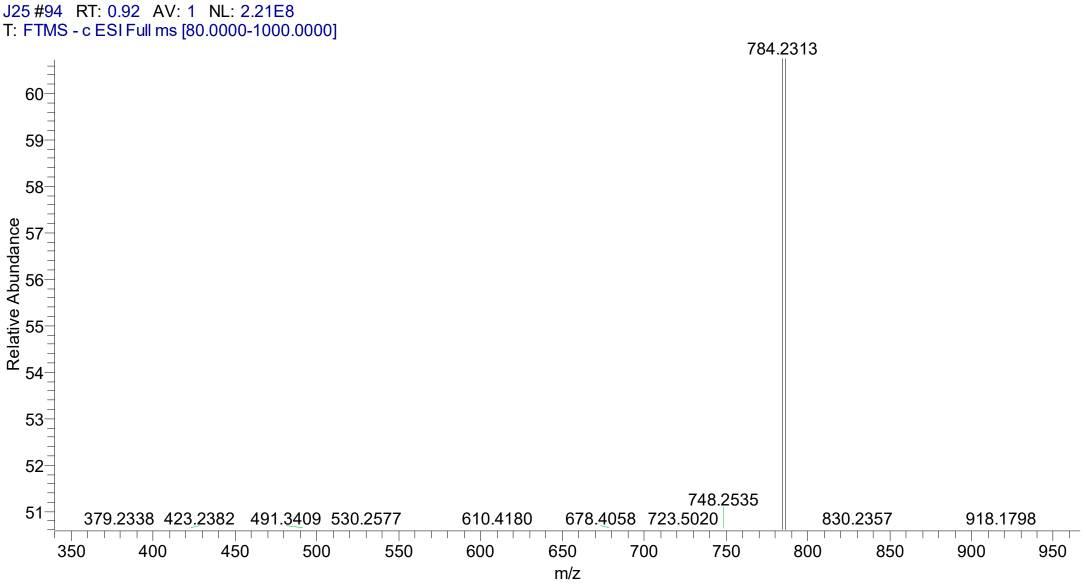

Figure S84.HR Mass spectrum (ESI) of compound **22c**.

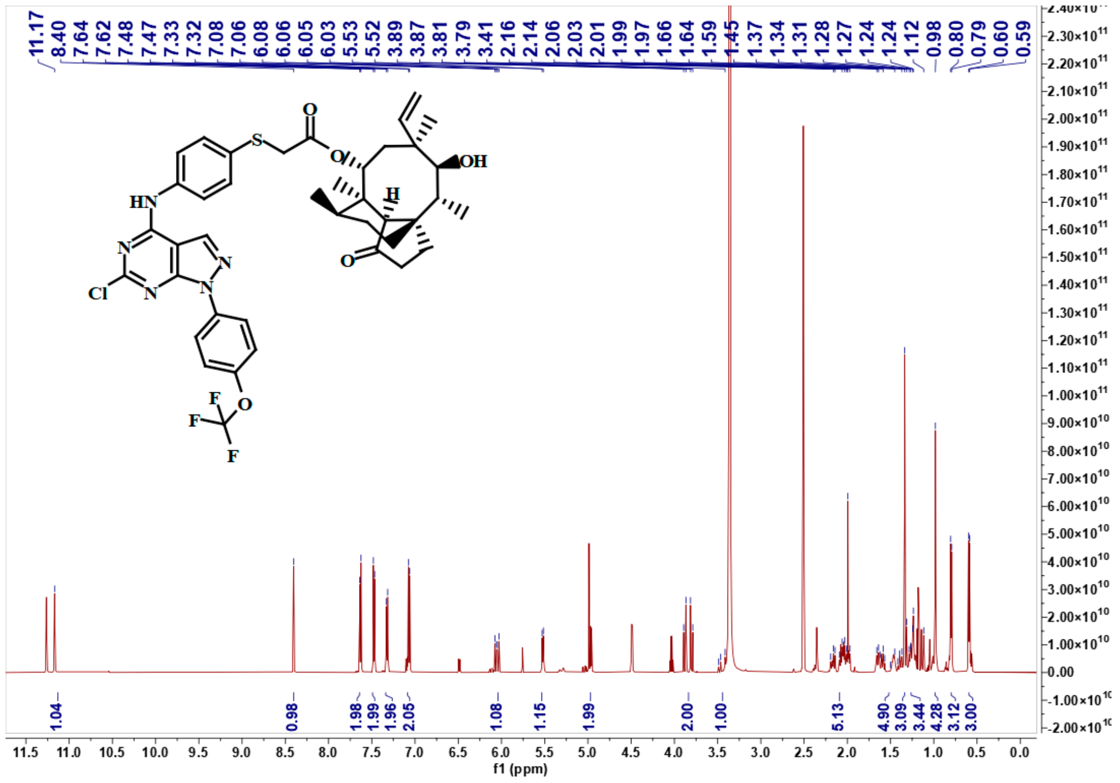

Figure S85.^{1}H-NMR spectrum (DMSO, 600MHz) of compound **23c**.

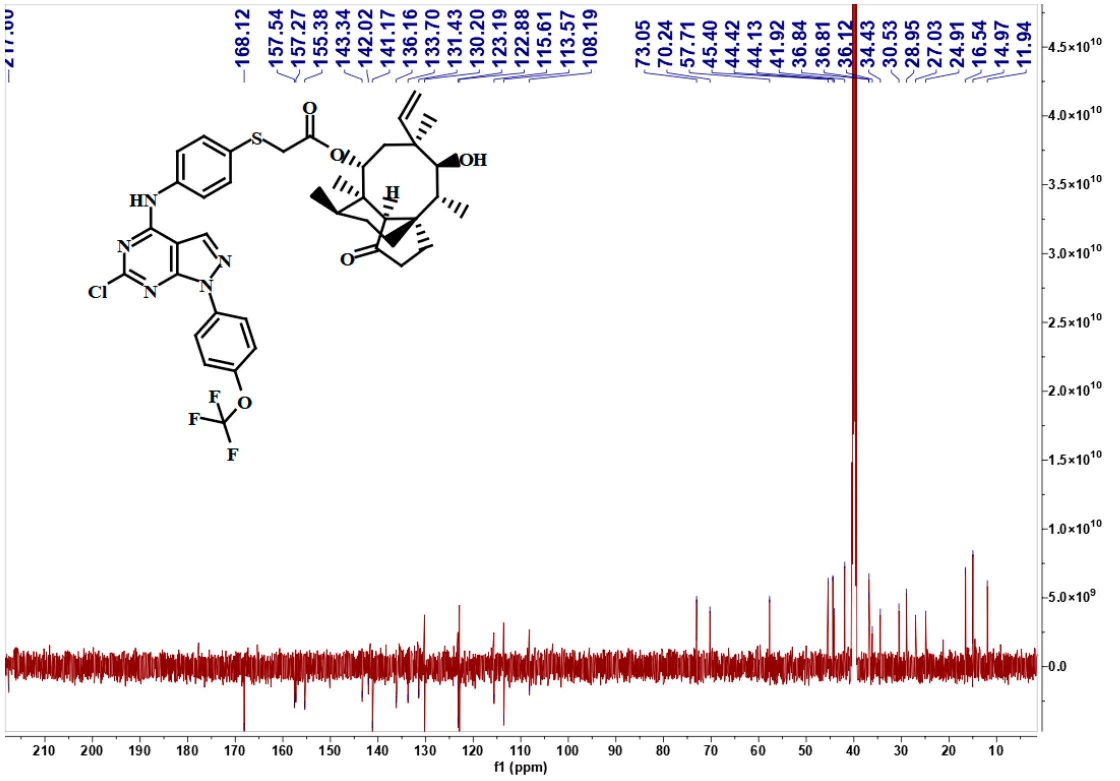

Figure S86.^{13}C-NMR spectrum (DMSO, 151MHz) of compound **23c**.

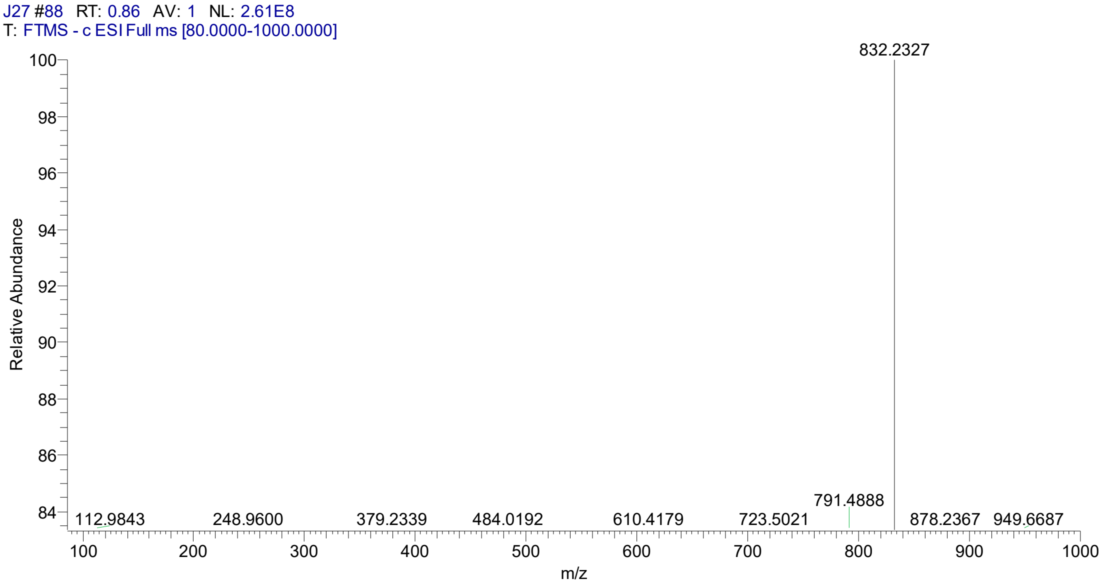

Figure S87.HR Mass spectrum (ESI) of compound **23c**.

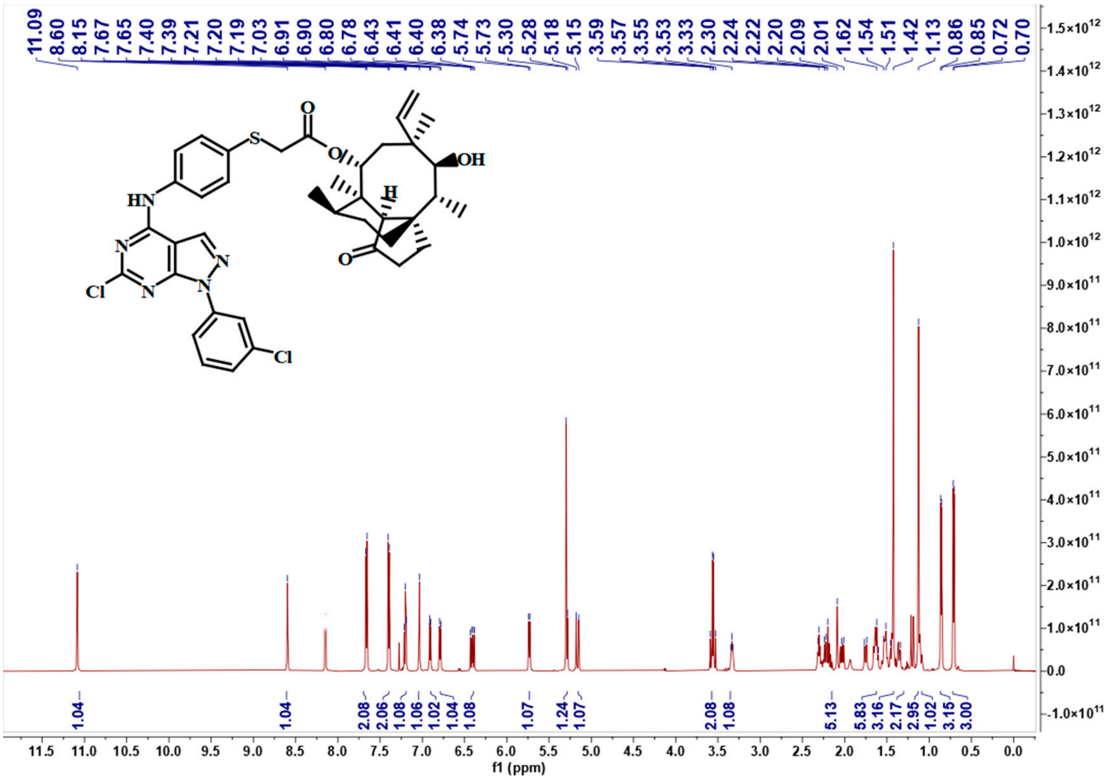

Figure S88.^{1}H-NMR spectrum ($CDCl_3$, 600MHz) of compound **24c**.

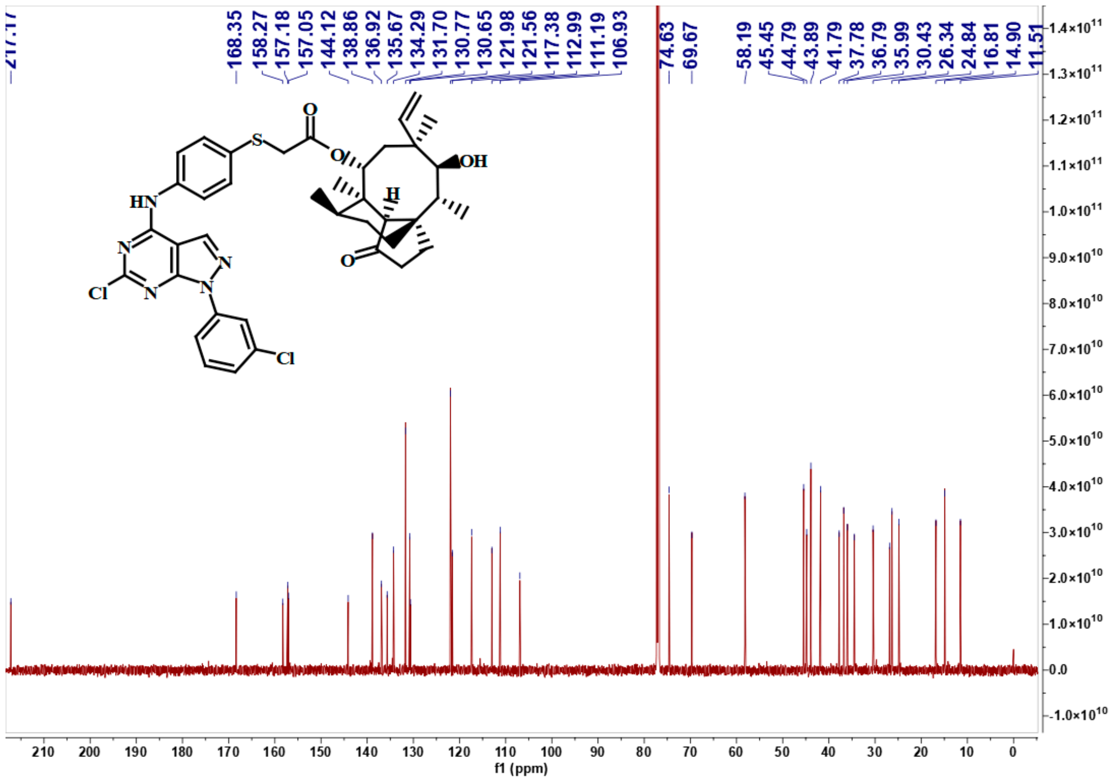

Figure S89.^{13}C-NMR spectrum ($CDCl_3$, 151MHz) of compound **24c**.

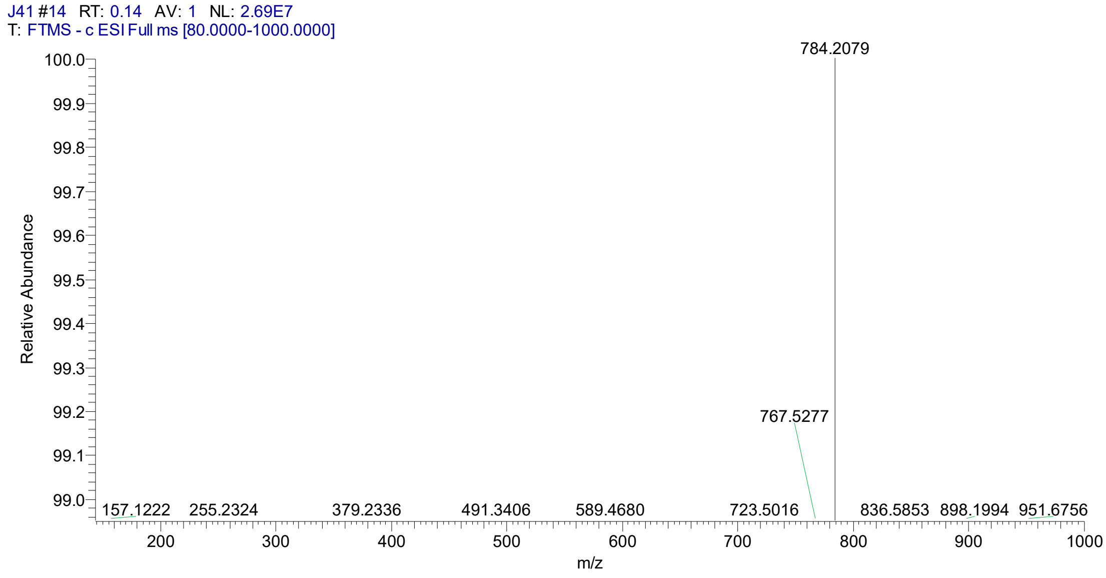

Figure S90.HR Mass spectrum (ESI) of compound **24c**.

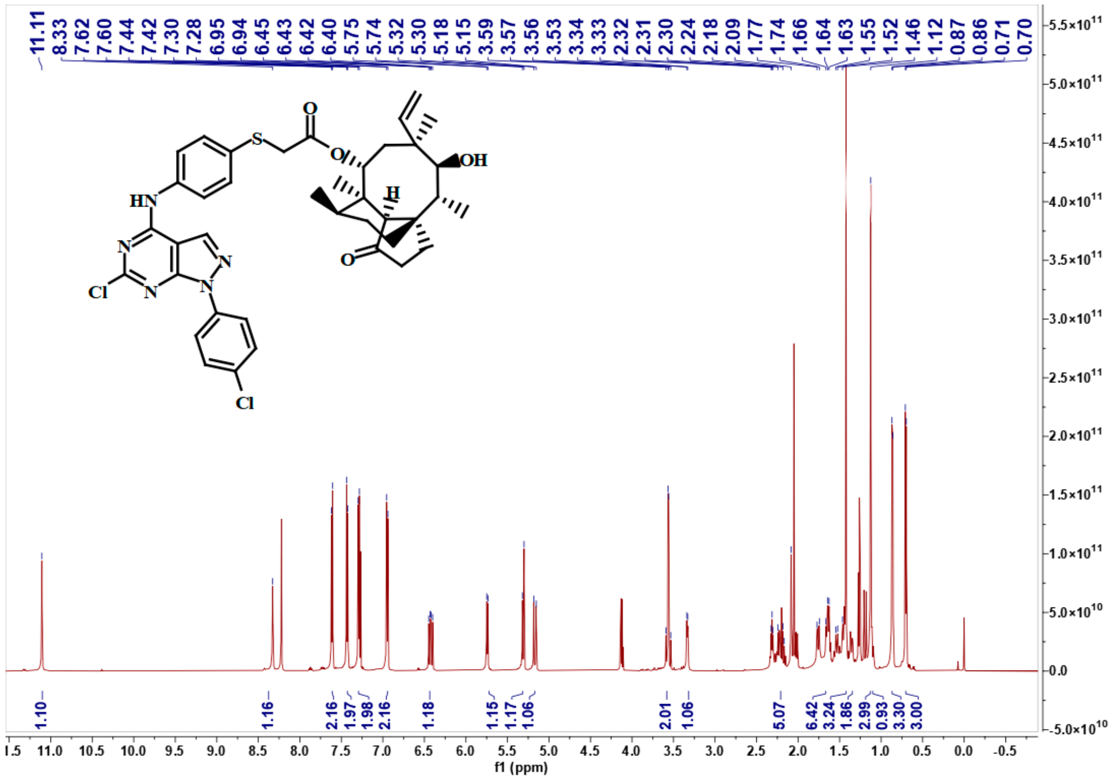

Figure S91.^{1}H-NMR spectrum (CDCl3, 600MHz) of compound **25c**.

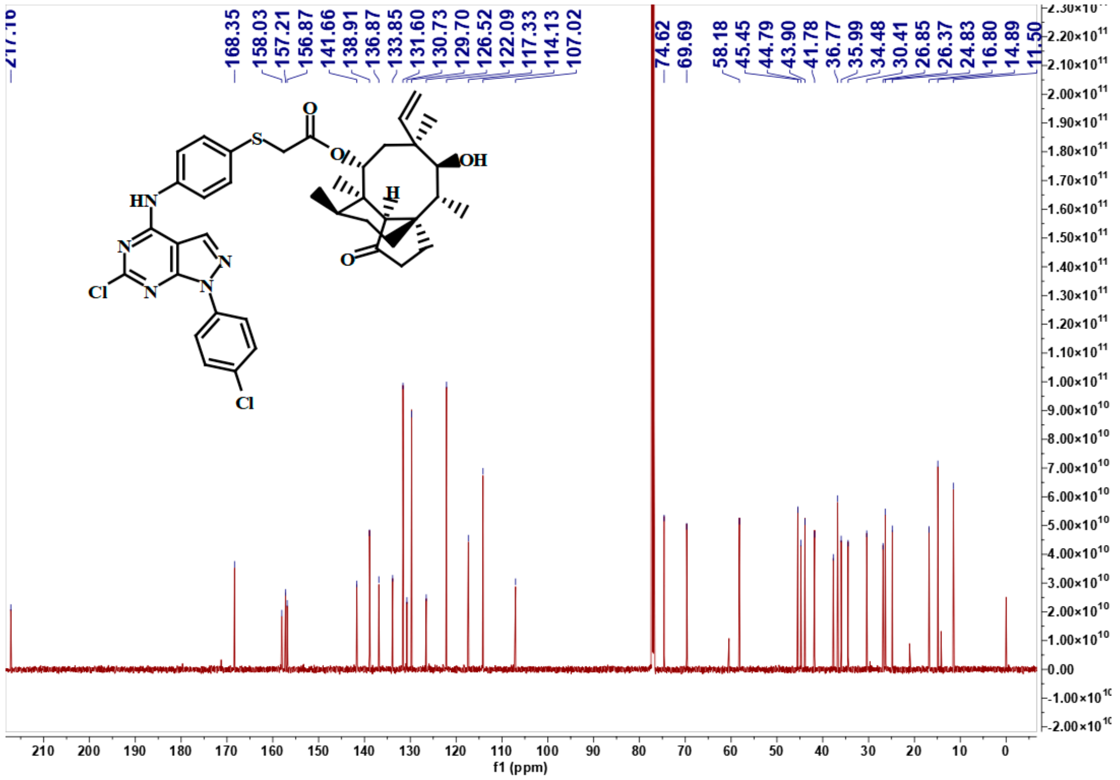

Figure S92.^{13}C-NMR spectrum (CDCl3, 151MHz) of compound **25c**.

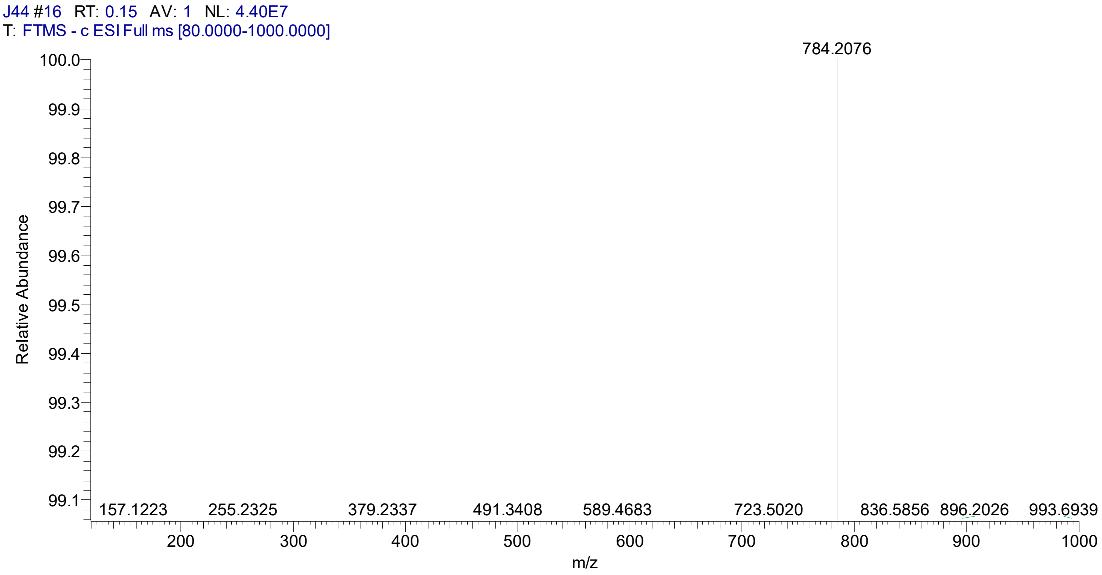

Figure S93.HR Mass spectrum (ESI) of compound **25c**.

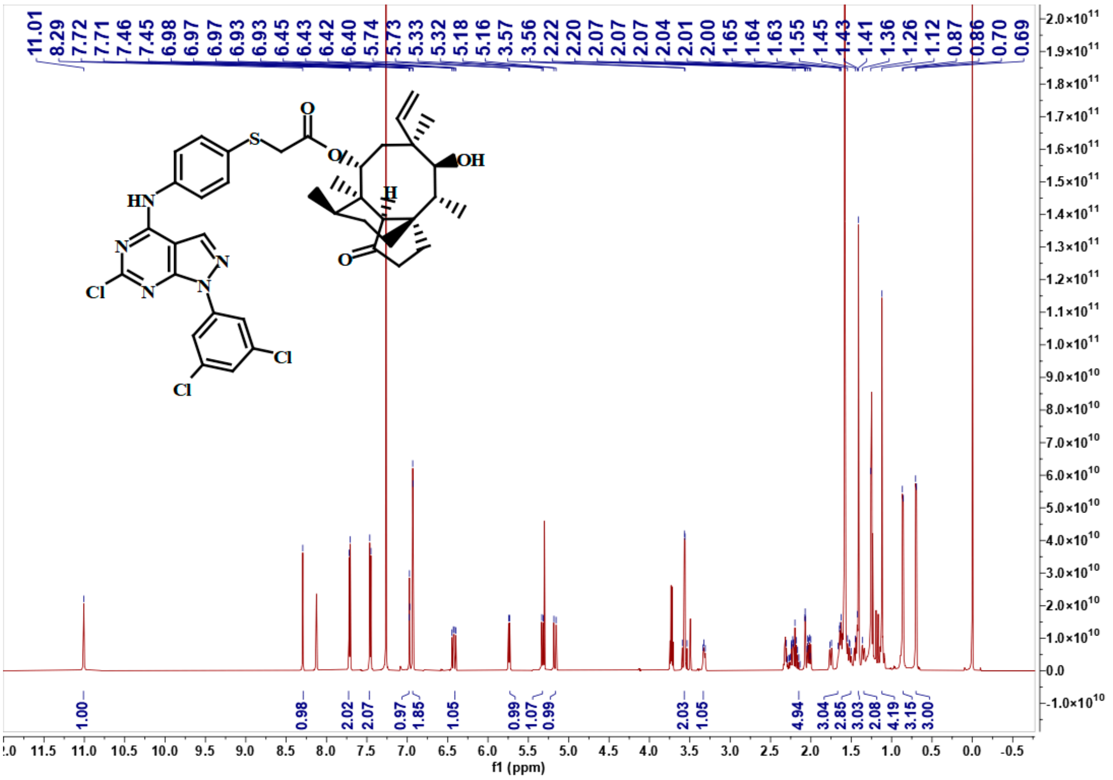

Figure S94.^{1}H-NMR spectrum ($CDCl_3$, 600MHz) of compound **26c**.

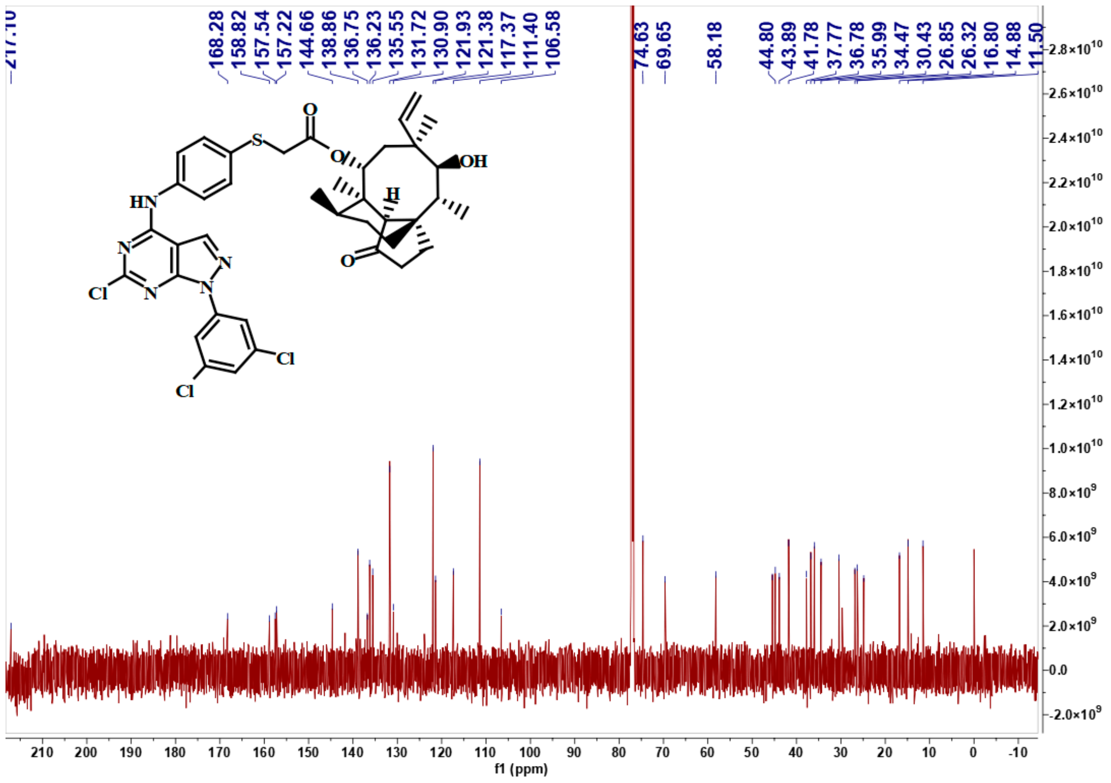

Figure S95.^{13}C-NMR spectrum ($CDCl_3$, 151MHz) of compound **26c**.

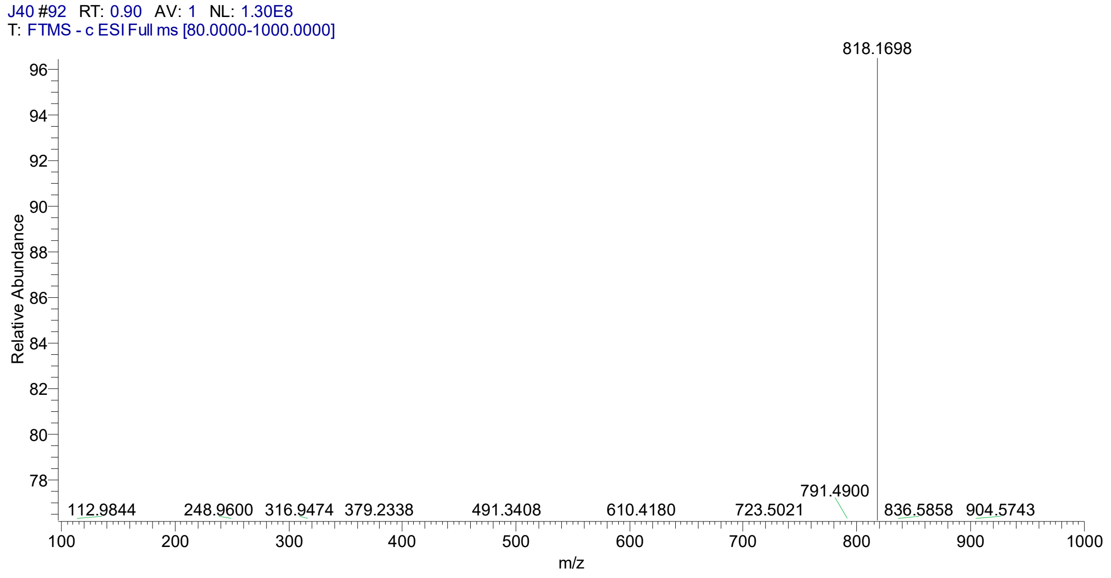

Figure S96.HR Mass spectrum (ESI) of compound **26c**.

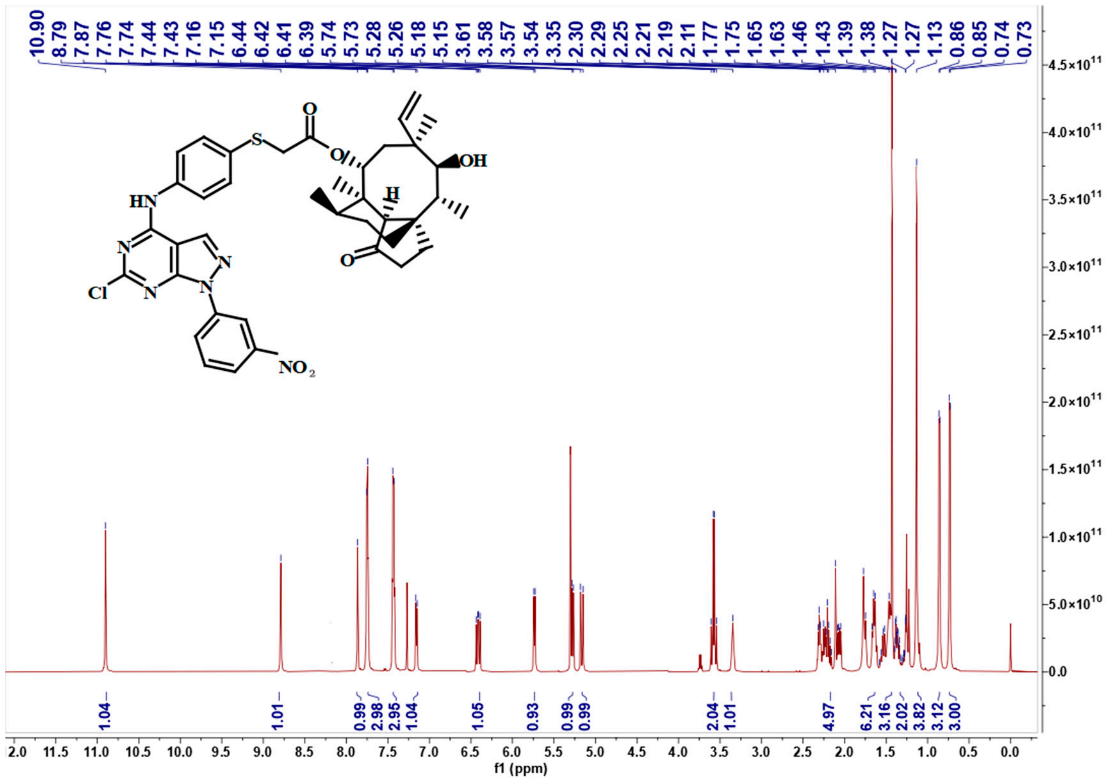

Figure S97.^{1}H-NMR spectrum ($CDCl_3$, 600MHz) of compound **27c**.

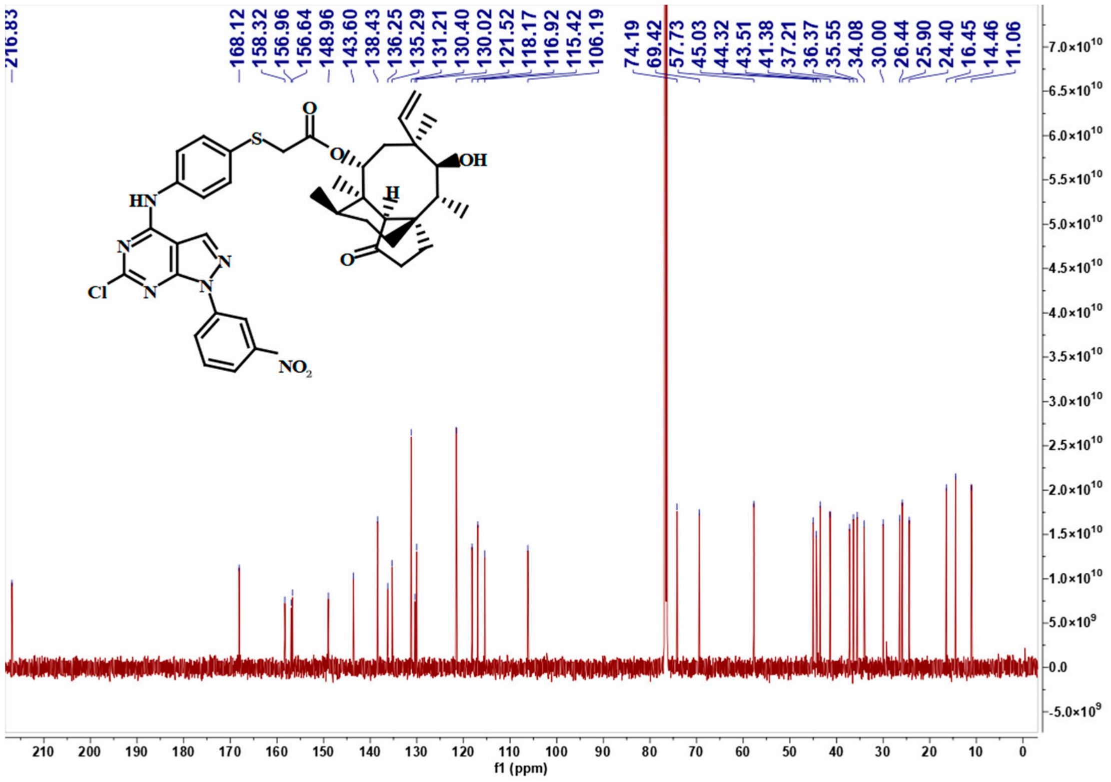

Figure S98.^{13}C-NMR spectrum (DMSO, 151MHz) of compound **27c**.

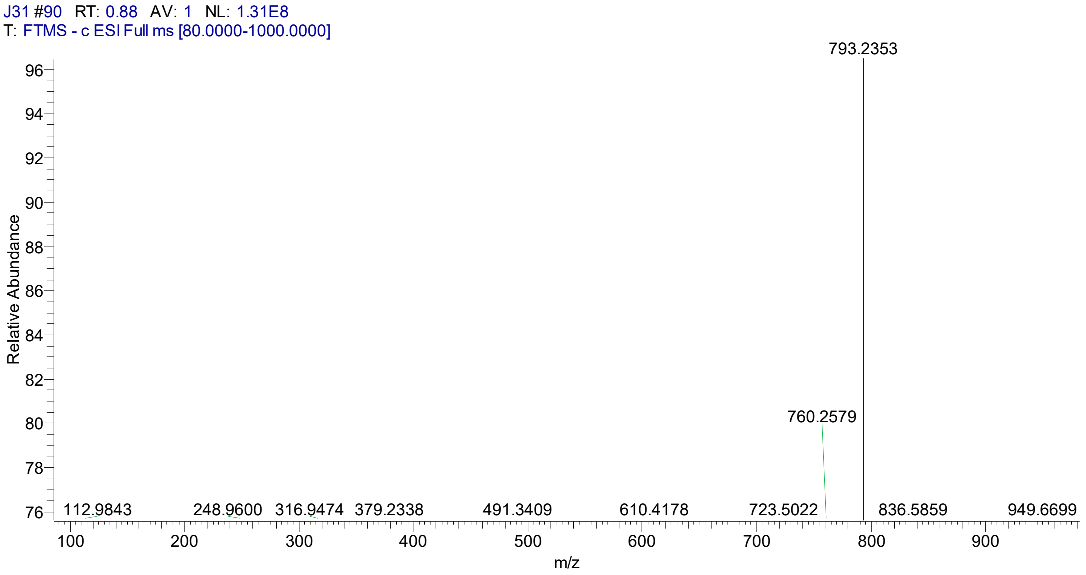

Figure S99.HR Mass spectrum (ESI) of compound **27c**.

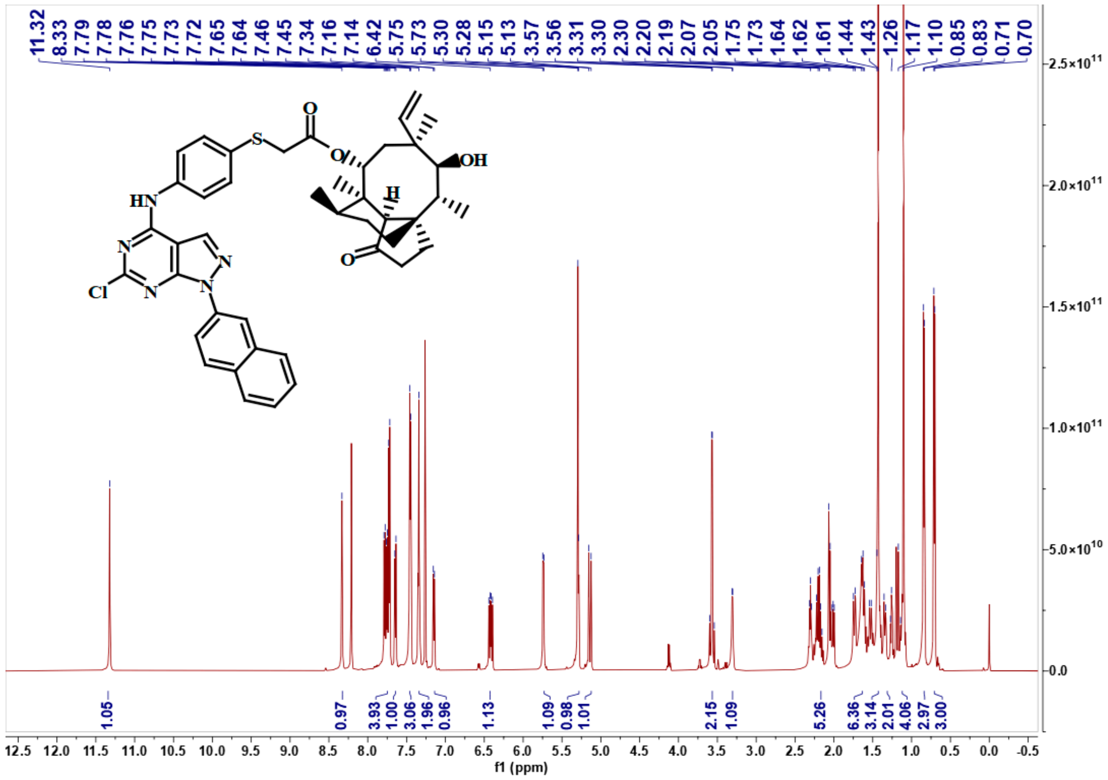

Figure S100.^{1}H-NMR spectrum ($CDCl_3$, 600MHz) of compound **28c**.

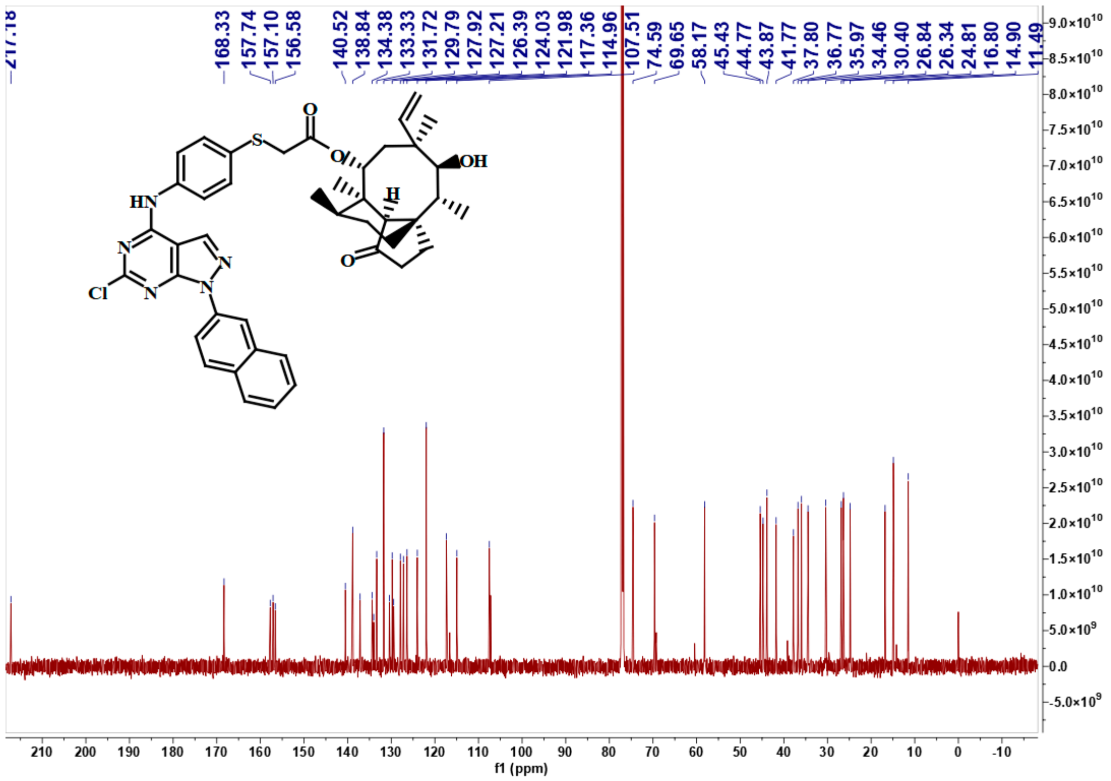

Figure S101.^{13}C-NMR spectrum ($CDCl_3$, 151MHz) of compound **28c**.

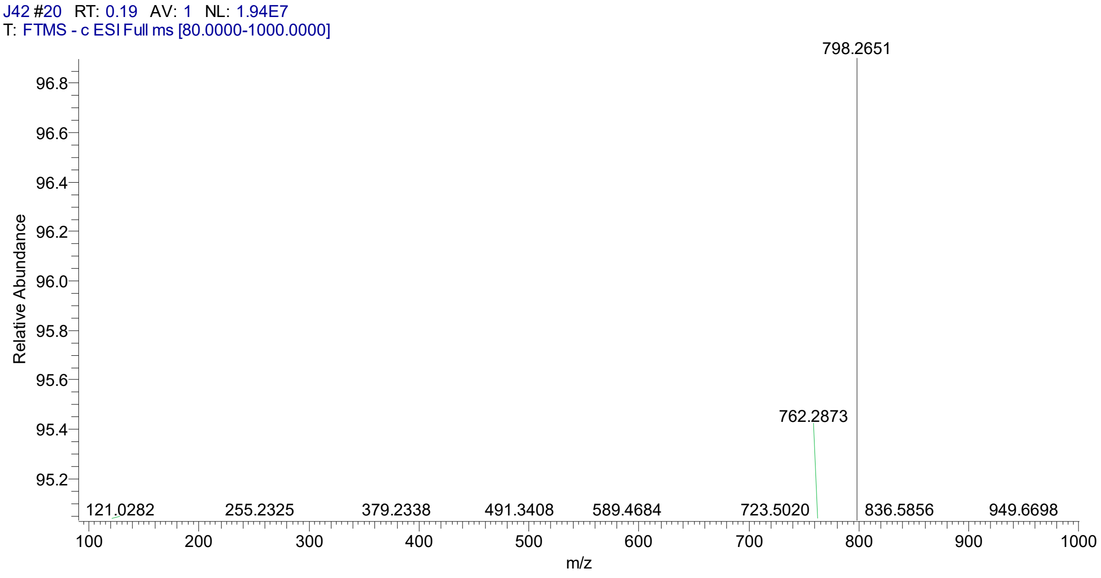

Figure S102.HR Mass spectrum (ESI) of compound **28c**.
